# BAI adhesion-GPCRs perform distinct functions in neural development differentially controlled by RTN4R and C1ql ligands

**DOI:** 10.1038/s41467-025-67453-6

**Published:** 2025-12-23

**Authors:** Jie Wang, Jinzhao Wang, Yi Miao, Yang Li, Shaoyuan Zhu, Yu Zhang, Ahmed Yousif, Min Huang, Marius Wernig, Thomas C. Südhof

**Affiliations:** 1 Department of Molecular and Cellular Physiology, Stanford University School of Medicine, Stanford, CA USA; 2 Howard Hughes Medical Institute, Stanford University School of Medicine, Stanford, CA USA; 3 Division of Life Science, The Hong Kong University of Science and Technology, Hong Kong, China; 4 Institute for Stem Cell Biology and Regenerative Medicine, Department of Pathology, Stanford University School of Medicine, Stanford, CA USA; 5 Department of Biomedical Engineering, Stanford University, Stanford, CA USA

**Keywords:** Cellular neuroscience, Molecular neuroscience

## Abstract

BAI1, BAI2, and BAI3 (for ‘Brain-specific Angiogenesis Inhibitor-1, -2, and -3’) are adhesion-GPCRs implicated in neuronal development. The precise roles of individual BAIs remain unclear. BAIs interact with two sets of ligands, secreted C1ql proteins and membrane-bound RTN4R proteins (a.k.a. NoGo receptors), but which of these ligands regulate specific functions of BAIs is incompletely understood. To address these key questions, we here systematically examine the functions of the three BAIs in neuronal development using hippocampal neuron-glia cultures, genetic knockouts, and rescue experiments. In a direct comparison, we demonstrate that deletions of BAI1 or BAI3, but not of BAI2, increase axonal and dendritic arborizations but decrease excitatory synapse formation, while inhibitory synapse formation remains unaffected. Since biochemical and cellular assays reveal that only BAI3 binds to both RTN4Rs and C1qls, we analyzed the role of these two ligands in controlling BAI3 functions using rescue experiments. We find that RTN4R-binding to BAI3 is essential for restricting axonal and dendritic arborizations and for enabling excitatory synapse formation, whereas C1ql-binding to BAI3 is only required for synapse organization as monitored in hippocampal neuron-glia cultures. Thus, BAI1 and BAI3 perform diverse functions that shape multiple facets of neuronal development and that require their interaction with RTN4Rs.

## Abstract (web summary)

This study shows that the deletions of BAI1 and BAI3, but not of BAI2, enhance axonal and dendritic growth while reducing excitatory synapse formation in murine mixed neuron-glia cultures. BAI3's interaction with specific ligands is crucial for these processes, highlighting its key role in neuronal development.

## Introduction

Brain-specific angiogenesis inhibitors (BAI1-3; gene symbols in mice *Adgrb1-3*) are adhesion-GPCRs that constitute the second largest GPCR family but remain poorly understood^1–3^. BAIs contain a large extracellular region comprising a distinct N-terminal domain (NTD), five (BAI1) or four (BAI2 and BAI3) thrombospondin type-1 repeats, a hormone-binding domain, and a GAIN domain^4^. Following their extracellular sequences, BAIs comprise a typical 7-transmembrane GPCR module and a large C-terminal cytoplasmic sequence. BAIs are almost exclusively expressed in brain (hence their name) where they were initially described as anti-angiogenesis and anti-oncogenic proteins^5–8^, but have since been associated with multiple diverse physiological and pathological roles^1–3^. BAI1 knockout (KO) mice exhibit deficits in hippocampus-dependent spatial learning and memory^9,10^, while BAI1 knockdown enhances dendritic arborization in hippocampal pyramidal cells^11^ and decrease excitatory synapse numbers^12,13^. In addition, BAI1 has been proposed to function as a phosphatidylserine receptor in macrophages and as a cell-fusion catalyst in myoblasts and oocytes^14–16^, although some uncertainty exists about these BAI1 functions because many phosphatidylserine receptors on macrophages are described, BAI1 is primarily expressed in brain^17^, and macrophages likely do not express BAI1^18^. BAI2-mutant mice, conversely, show antidepressant-like behaviors and exhibit behavioral abnormalities^19,20^. BAI3 finally was implicated in synapse formation since conditional deletion of postsynaptic BAI3 from Purkinje cells or olfactory bulb granule cells suppressed synapse numbers^21–23^. Moreover, BAI3 restricts dendritic growth both in cultured neurons and in vivo^24^. Despite accumulating evidence supporting the involvement of BAIs in various brain functions, a systematic direct comparison with the same experimental approach of the functions of individual BAIs is lacking. Indeed, the diverse phenotypes observed in individual BAI1, BAI2, and BAI3 KO brains do not reveal a common function and raise the question whether different BAIs are functionally related or distinct.

Two families of ligands were reported for BAI3: Secreted C1q-like proteins (C1ql1-4)^25^ and membrane-bound reticulon-4 receptors (RTN4Rs comprising RTN4R, RTN4RL1 and RTN4RL2, a.k.a. NoGo receptors)^26,27^. Multiple studies document a fundamental requirement for C1ql-binding to BAI3 in selected central synapses^21–23,28^. In these studies, deletion of either presynaptic C1qls or postsynaptic BAI3 severely impaired climbing fiber synapses in the cerebellum^21^ and synaptic connections between the accessory olfactory nucleus and olfactory bulb granule cells^22^ or between the basolateral amygdala (BLA) and the medial prefrontal cortex (mPFC)^28^. In addition to binding to BAIs, C1qls were shown to bind to the kainate-type glutamate receptor GluK2 and GluK4 and to neurexin-3^29^. Thus, extensive available information demonstrates the importance of C1ql-binding to BAI3 in synapse organization. However, these data do not reveal whether C1ql-binding to other BAIs might be functionally important and whether C1ql-binding has a role in the axonal and dendritic arborization phenotype observed in BAI1- and BAI3-deficient neurons.

Different from C1ql-binding to BAI3, the significance of RTN4R-binding to BAIs has not been explored. RTN4Rs were initially characterized as “NoGo” receptors that inhibit axonal growth by interacting with the surface-exposed endoplasmic reticulum protein RTN4^30–32^. Various other RTN4R *trans*-ligands (for example, myelin-associated glycoprotein and oligodendrocyte-myelin glycoprotein) and *cis*-signaling receptors (for example, p75 and LINGO-1) have also been reported^33–36^. Deletions or mutations of RTN4Rs in mice lead to alterations in cognitive behaviors, dendritic morphology, synapse formation and synaptic plasticity^37–41^. Moreover, in human neurons, deletion of RTN4RL1 and RTN4RL2 produced a large increase in axonal and dendritic arborizations and a decrease in synapse numbers^27^. Furthermore, the crystal structure of the BAI1-RTN4R complex revealed an unusual binding interface in which unique carbohydrate modifications of the 3^rd^ TSR of BAI1 were required for the interaction^27^, while biophysical experiments demonstrated that in addition to BAI1, BAI3 also binds to RTN4Rs whereas BAI2 fails to bind^27^. Viewed together, these results are consistent with the hypothesis that binding of RTN4Rs to BAIs mediates the functions of BAIs in neurite growth and in synapse formation that were observed in BAI1-deficient neurons^11–13^. However, this hypothesis has not been directly tested. Moreover, these results do not address the role of RTN4R-binding to BAI3 as opposed to BAI1 and the relative importance of C1ql- vs. RTN4R-binding to various BAIs.

Here, we set out to address two major questions resulting from previous studies on BAIs: What are the relative functions of different BAIs in neuronal development, and what are the specific roles of the two BAI ligands, C1qls and RTN4Rs in these functions of BAIs? To answer these two questions, we systematically analyzed the essential contributions of BAI1, BAI2, and BAI3 to neuronal development in dissociated hippocampal cultures using genetic knockouts (KOs) and investigated the mechanism of action of BAI3 as the only BAI isoform that binds to both RTN4Rs and C1qls. Using primary neuron-glia cultures from the hippocampus of newborn mice as a reduced system, we found that BAI1 and BAI3, but not BAI2, promote excitatory synapse formation and synaptic transmission, and that BAI1 and BAI3, but again not BAI2, also restrict axonal and dendritic growth. Notably, BAI3 mutants unable to bind to RTN4Rs could not rescue the phenotypes of BAI3-deficient hippocampal cultures, whereas a BAI3 mutant unable to bind to C1qls was able to rescue all phenotypes except for synaptic transmission. Our data thus suggest that BAI1 and BAI3 regulate axonal and dendritic arborizations and excitatory synapse formation primarily via interactions with RTN4Rs, whereas the binding of C1qls to BAI3 but not BAI1 provides an additional function by mediating synapse organization.

## Results

### Generation of precisely matching neuron-cultures containing or lacking BAI1, BAI2, or BAI3

Our previous crystal structure of the RTN4R-BAI1 complex as well as binding measurements and mutagenesis experiments demonstrated that RTN4Rs bind to BAI1 and BAI3, but not BAI2^26,27^. Moreover, our recent cryo-EM structure of the C1ql3-BAI3 complex showed that BAI2 and BAI3 share identical interface residues for binding to C1qls whereas BAI1 lacks these residues, suggesting that BAI1 does not bind to C1qls^42^. To validate this structural prediction, we purified the C1q-like domain of C1ql3 and extracellular fragments of BAI1, BAI2, and BAI3. The extracellular fragments included the N-terminal region that contains the C1ql-binding site in BAI3^21^. We then performed size-exclusion chromatography of various proteins alone and in combination (Supplementary Fig. 1a–c). Consistent with the structural predictions, the BAI1 extracellular domains (ECDs) did not form a stable complex with the C1q-like domain of C1ql3. Conversely, both the BAI3 ECD and the BAI2 N-terminal fragment that includes its N-terminal domain (NTD) and thrombospondin repeats (TSRs) formed complexes with the C1q-like domain of C1ql3, with the BAI2-C1ql3 complex exhibiting less stability than the BAI3-C1ql3 complex (Supplementary Fig. 1a–c).

Next, we systematically assessed the binding of all C1qls to the BAIs using a cell-surface labeling assay. All C1qls (C1ql1-C1ql4), when added at 100 nM, strongly bound to HEK293T cells expressing BAI3, whereas they bound only marginally to HEK293T cells expressing BAI2 and not detectably to HEK293T cells expressing BAI1 (Supplementary Fig. 2). Together with previous data^21,26,27,42^, these results suggest that BAI1 binds tightly to RTN4Rs but not to C1qls, BAI2 binds weakly to C1qls but not to RTN4Rs, and BAI3 binds tightly to both RTN4Rs and C1qls.

Given the distinct ligand-binding properties of BAI1, BAI2 and BAI3, two questions arise: Do BAI1, BAI2, and BAI3 have similar or distinct functions, and how do these functions correlate with their respective ligand binding profiles? To systematically address these questions, we analyzed the phenotypes of BAI1, BAI2, and BAI3 deletions in primary neuron-glia cultures obtained from newborn mice as a reduced system.

For BAI1 deletions, we used BAI1 mutant mice that we designed as floxed conditional knockout (KO) mice^27^ but that for unknown reasons failed to express BAI1 even without Cre recombinase, effectively rendering them constitutive KOs. To generate ‘clean’ constitutive BAI1 KO mice, we crossed the BAI1 mutant mice with CMV-Cre mice that express Cre-recombinase in the germline^43^, thereby removing the exon 2 that contains the initiator methionine and signal peptide of BAI1 (Supplementary Fig. 3a). We observed no significant differences in genotype distributions between male and female BAI1 wild-type, heterozygous and homozygous KO mice assuming Mendelian inheritance, consistent with previous studies suggesting that the BAI1 deletion does not impair survival (Supplementary Fig. 3e, 3f)^9,10^. Moreover, we detected no significant differences in body weight between the three genotypes for male and female mice at P21 (Supplementary Fig. 3g). Based on these findings, we felt confident to analyze mixed neuron-glia cultures produced from the hippocampi of littermate newborn wild-type and BAI KO mice that were generated by heterozygote crossings, and we confirmed in these cultures using quantitative RT-PCR that the BAI1 KO completely deleted BAI1 expression (Supplementary Fig. 3d).

For BAI2 and BAI3 deletions, we utilized previously described BAI2 and BAI3 conditional KO (cKO) mice targeting exons 5-8 and exons 8-9, respectively (Supplementary Fig. 3b, c)^21^. We infected mixed neuron-glia cultures produced from the hippocampi of newborn BAI2 or BAI3 cKO mice at DIV4 with lentiviruses expressing active wild-type (Cre) or inactive mutant Cre-recombinase (ΔCre; control) under control of the synapsin-1 promoter. Both ΔCre and Cre are expressed as EGFP-fusion proteins containing a nuclear localization sequence. We confirmed nearly complete Cre recombination in both BAI3 and BAI2 cKO cultures by quantitative RT-PCR (Supplementary Fig. 3d). Although the BAI3 cKO mice were designed to delete exons 8 and 9 (Supplementary Fig. 3c) and thus could potentially produce a truncated protein, the exon 8 and 9 deletions cause a premature stop codon that disrupts the Hormone-Binding Domain of BAI3 (Supplementary Fig. 3h, i). Presumably as a result of the misfolded partial hormone-binding domain, the KO suppresses 5’ BAI3 mRNA levels, suggesting nonsense-mediated decay (Supplementary Fig. 3j). Together with previous data showing that the BAI3 KO abolishes the detection of the protein by immunogold electron microscopy^21^, these data suggest that the BAI3 KO does not produce a stable secreted truncated protein.

Since many of our experiments depend on efficient expression of Cre recombinase, we next aimed to evaluate the transduction efficiency of lentiviruses in neurons and glial cells in our experiments. We stained the lentivirally infected hippocampal cultures from BAI3 cKO mice with DAPI and NeuN antibodies. Nearly all neurons identified as NeuN- and DAPI-positive cells (>97%) and most glial cells identified as NeuN-negative but DAPI-positive cells (~80%) were successfully infected and expressed the EGFP-Cre fusion protein that is driven by the synapsin promoter (Supplementary Fig. 4a, b). In addition, we validated Cre-recombination of the BAI3 gene using genomic PCR (Supplementary Fig. 4c, d), which confirmed nearly complete Cre recombination. Overall, these results indicate that the human synapsin promoter-driven Cre is also expressed in glial cells in the hippocampal cultures, with the lower overall expression efficiency in glia at least in part due to poor infection of microglia by lentiviruses. In this manner we thus produced here precisely matching pairs of control and BAI2-deficient or BAI3-deficient neuron-glia cultures, as well as precisely matching control and BAI1-deficient cultures, suitable for a systematic analysis of phenotypes.

### Systematic analysis of BAI1-BAI3 functions in neuronal development

**Fig. 1:**
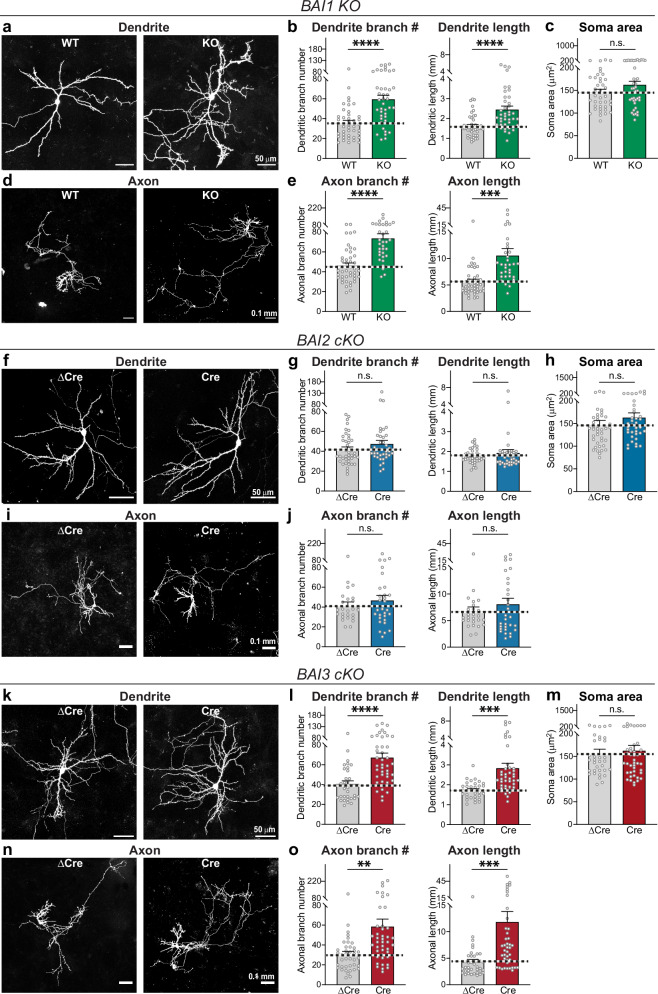
Deletions of BAI1 and BAI3, but not of BAI2, cause exuberant arborization of axons and dendrites in cultured hippocampal neurons. **a–c** Representative images (**a**), summary graphs of dendritic branch numbers and dendrite length (**b**), and summary graph of the soma size (**c**) of hippocampal neurons that were cultured together with glia from littermate wild-type (WT) and BAI1 knockout (KO) mice, sparsely transfected with tdTomato-expressing plasmid at DIV 9, and imaged at DIV 14 (n [cells/experiments] for all graphs = 41/5, p = 3.44X10^-6^, 4.63X10^-5^ and 0.11 from left to right). **d**,**e** Same as (**a**,**b**), but for axons (n [cells/experiments] = 44/4 for WT; 35/4 for KO; p = 5.61X10^-7^ and 2.31X10^-4^). **f–j** Same as (**a–e**), except that the neurons and glia were cultured from BAI2 conditional knockout (cKO) mice that were infected with lentiviruses expressing mutant Cre-recombinase (control [ΔCre]) or active Cre-recombinase (test [Cre]) at DIV4 before being transfected and imaged as described for **a–e** (n [cells/experiments for ΔCre & Cre conditions, respectively] for **g** = 41/4 & 38/4; **h** =41/4 & 38/4; **j** = 26/4 & 30/4). p = 0.21 & 0.34 for **g**, 0.27 for **h**, and 0.42 & 0.34 for **j**. (**k–o**) Same as **f–j**, except that the neurons analyzed were cultured from BAI3 cKO mice (n [cells/experiments for ΔCre & Cre conditions, respectively] for **l** = 34/4 & 42/4; **m** = 34/4 & 42/4; **o** = 40/4 & 42/4). p = 1.59X10^-5^ & 0.0002 for **l**, 0.69 for **m**, and 0.0012 & 0.0005 for **o**. All numerical data are means ± SEM. Dots indicate individual cells that were analyzed. All statistical analyses were performed by unpaired two-tailed Student’s t-tests (n.s. not significant, ** *p*<0.01, *** *p*<0.001, **** *p*<0.0001).

To assess the effect of the BAI1-3 deletions on neuronal development, we sparsely labeled neurons in matching control and BAI1-, BAI2- or BAI3-deficient neuron-glia cultures by transfection with plasmids expressing tdTomato at DIV9. We then analyzed the transfected neurons by quantifying axons, dendrites, and soma sizes at DIV14 (Fig. 1). As always, all analyses were performed by experimenters on anonymized samples.

Strikingly, both the BAI1 and the BAI3 deletions caused an approximately 0.5- to 1.7-fold increase in the length of dendrites and axons, whereas the BAI2 deletion had no effect (Fig. 1). Dendritic and axonal branch numbers were consistently increased by the BAI1 and BAI3 deletions, as were dendrite and axon lengths, with the biggest effects observed on axons of BAI3-deficient neurons that exhibited a large (~1.7-fold) increase in length (Fig. 1). None of the deletions altered the neuronal soma size (Fig. 1). These findings indicate that BAI1 and BAI3 control dendritic and axonal growth whereas BAI2 does not play an essential role in these processes.

**Fig. 2:**
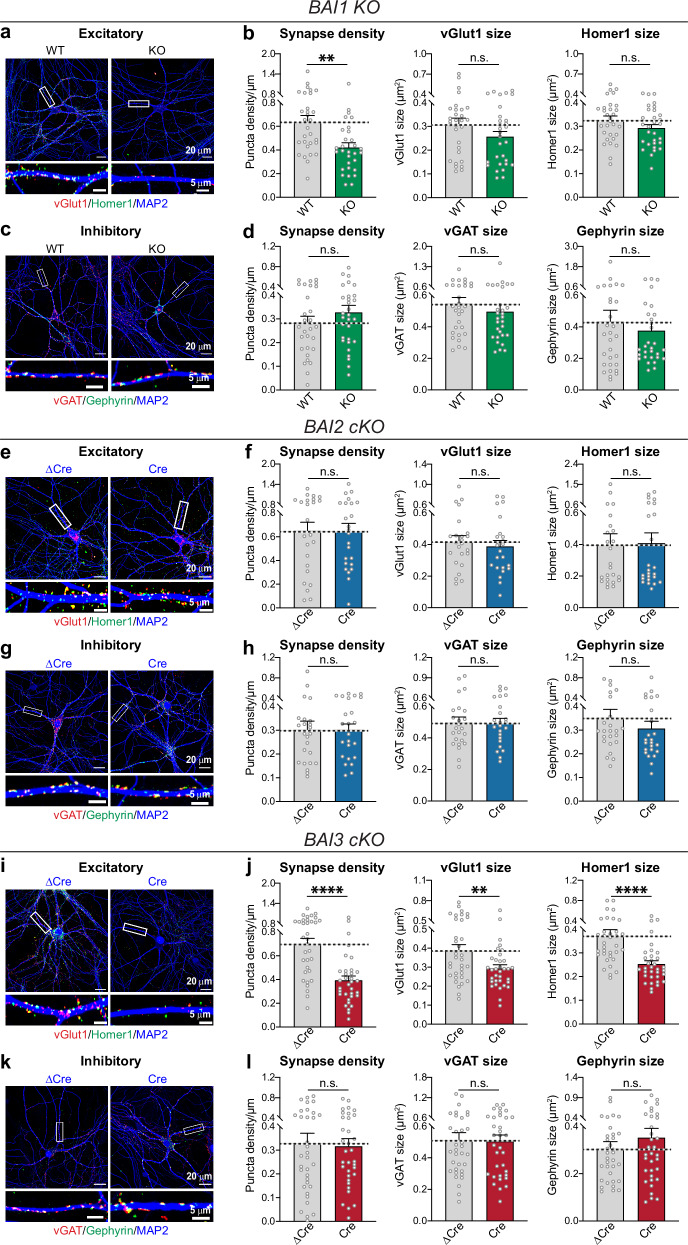
Deletions of BAI1 and BAI3, but not of BAI2, dramatically decrease the density of excitatory but not inhibitory synapses. **a** Representative images of hippocampal neurons in mixed neuron-glia cultures obtained from littermate wild-type (WT) and BAI1 knockout (KO) mice. Neurons were stained at DIV 14 for MAP2 (blue), vGlut1 (red), and Homer1 (green) as excitatory synapse markers. **b** Summary graphs of the excitatory synapse density (left, defined as puncta positive for both vGlut1 and Homer1 signals), vGlut1 puncta size (middle), and Homer1 puncta size (right) for experiments illustrated in **a** (n’s [cells/experiments]: 30/3 and 30/3 for WT and KO). p = 0.0014, 0.1342 and 0.1326 from left to right. **c** Representative images of hippocampal neurons in mixed neuron-glia cultures obtained from littermate wild-type (WT) and BAI1 KO mice. Neurons were stained at DIV 14 for MAP2 (blue), vGAT (red), and gephyrin (green) as inhibitory synapse markers. **d** Summary graphs of the inhibitory synapse density (left, defined as puncta positive for both vGAT and gephyrin signals), vGAT puncta size (middle), and gephyrin puncta size (right) for experiments illustrated in **c** (n’s [cells/experiments]: 30/3 and 30/3 for WT and KO). p = 0.2894, 0.3902 and 0.5274 from left to right. **e**,**f** Same as (**a**,**b**), except that neuron-glia cultures were obtained from BAI2 conditional knockout (cKO) mice and were additionally infected at DIV 4 with lentiviruses expressing mutant Cre-recombinase (control [ΔCre]) or active Cre-recombinase (test [Cre]) (in **f**, n’s [cells/experiments]: 24/2 and 24/2 for ΔCre and Cre). p = 0.9642, 0.5688 and 0.9252 from left to right. **g**,**h** Same as (**c**,**d**), except that neuron-glia cultures were obtained from BAI2 cKO mice and were additionally infected at DIV 4 with lentiviruses expressing mutant Cre-recombinase (control [ΔCre]) or active Cre-recombinase (test [Cre]) (in **h**, n’s [cells/experiments]: 24/2 and 24/2 for ΔCre and Cre). p = 0.9957, 0.9524 and 0.3372 from left to right. (**i**, **j**) Same as (**e**, **f**), except that neuron-glia cultures were obtained from BAI3 cKO mice (in **j**, n’s [cells/experiments]: 34/3 and 34/3 for ΔCre and Cre). p = 1.88X10^-6^, 0.007 and 8.43X10^-5^ from left to right. **k**,**l** Same as (**g**,**h**), except that neuron-glia cultures were obtained from BAI3 cKO mice (in **l**, n’s [cells/experiments]: 34/3 and 34/3 for ΔCre and Cre). p = 0.794, 0.8989 and 0.3408 from left to right. All numerical data are means ± SEM. Dots indicate individual cells that were analyzed. All statistical analyses were performed by unpaired two-tailed Student’s t-tests (n.s. not significant, ** *p*<0.01, **** *p*<0.0001).

Next, we analyzed the density of synapses in BAI1-, BAI2- and BAI3-deficient hippocampal cultures at DIV14 using immunocytochemistry. We stained control and BAI1-, BAI2- and BAI3-deficient hippocampal cultures obtained as described above for a variety of synaptic markers, using presynaptic vGluT1 and postsynaptic Homer1 as excitatory synapse markers and presynaptic vGAT and postsynaptic gephyrin as inhibitory synapse markers (Fig. 2). We then quantified excitatory and inhibitory synapse densities as ‘puncta’ containing co-localized vGlut1 and Homer1 or co-localized vGAT and gephyrin, respectively, which is the most rigorous morphological approach to quantifying synapse numbers.

Deletion of both BAI1 and BAI3 resulted in a significant reduction (~35-45%) in excitatory but not in inhibitory synapse density, whereas deletion of BAI2 again had no effect (Fig. 2). The BAI1 and BAI2 deletions did not significantly alter the apparent sizes of vGlut1 or Homer1 puncta, whereas the BAI3 deletion decreased the sizes of both vGlut1 and Homer1 puncta by approximately 25% (Fig. 2). Thus, both BAI1 and BAI3 selectively support excitatory but not inhibitory synapse formation, whereas BAI2 does not appear to be essential. It is possible that the constitutive nature of the BAI1 deletion vs. the conditional, more acute nature of the BAI3 deletion accounts for the observed differences in puncta sizes or that these differences are a consequence of their distinct ligand interactions.

**Fig. 3:**
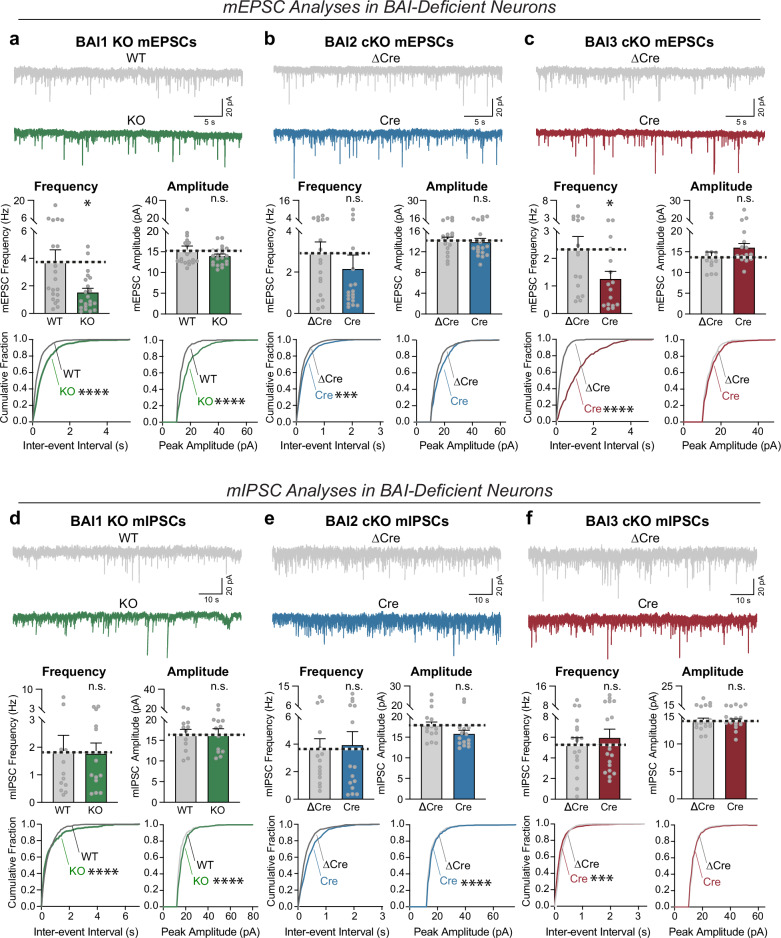
Deletions of BAI1 and BAI3, but not of BAI2, significantly suppress the frequency of miniature excitatory postsynaptic currents (mEPSCs) without affecting the mEPSC amplitude or miniature inhibitory postsynaptic currents (mIPSCs). **a** Representative traces (top), summary graphs of the frequency and amplitude (middle) and cumulative fraction plots of the inter-event interval and peak amplitude (bottom) of mEPSCs recorded from hippocampal neurons in mixed neuron-glia cultures obtained from littermate wild-type (WT) and BAI1 knockout (KO) mice (n [cells/experiments] = 20/4 for WT; 21/4 for KO). p = 0.0179, 0.2053, <0.0001 and <0.0001 from left to right then top to bottom. **b**,**c** Same as (**a**), except that mixed neuron-glia cultures were obtained from BAI2 (**b**) or BAI3 conditional knockout (cKO) mice (**c**) and were additionally infected at DIV 4 with lentiviruses expressing mutant Cre-recombinase (control [ΔCre]) or active Cre-recombinase (test [Cre]) (in **b**, n [cells/experiments] = 18/4 for both ΔCre and Cre; in **c**, n = 16/3 for both ΔCre and Cre). For BAI2 (**b**), p = 0.3573, 0.7034, 0.002 and 0.0763 from left to right then top to bottom. For BAI3 (**c**), p = 0.0394, 0.1495, <0.0001 and 0.3433 from left to right then top to bottom. **d–f** Same as (**a–c**), except that mIPSCs were monitored (in **d**, n [cells/experiments] = 12/3 for ΔCre and 13/3 for Cre conditions; in **e**, n = 15/3 for both ΔCre and Cre conditions; in **f**, n = 18/3 for both ΔCre and Cre conditions). For BAI (**d**), *p* = 0.9438, 0.9707, <0.0001 and <0.0001 from left to right then top to bottom. For BAI 2 (**e**), p = 0.8159, 0.1249, 0.3876 and <0.0001 from left to right then top to bottom. For BAI 3 (**f**), *p* = 0.5521, 0.9656, 0.0001 and 0.2213 from left to right then top to bottom. All numerical data are means ± SEM. Dots indicate individual datapoints. Statistical significance was determined by unpaired two-tailed Student’s t-tests (bar graphs) or the two-tailed Kolmogorov–Smirnov test at a 95% confidence level (cumulative distributions) (n.s., not significant, * *p*<0.05, *** *p*<0.001, **** *p*<0.0001).

To assess the effects of the BAI deletions on synaptic transmission, we performed whole-cell patch-clamp recordings of miniature excitatory postsynaptic currents (mEPSCs) and miniature inhibitory postsynaptic currents (mIPSCs) in BAI1-, BAI2-, and BAI3-deficient neurons. The BAI1 or BAI3 deletions decreased the mEPSC frequency (~60% and ~50%, respectively) without causing a major change in mEPSC amplitude (Fig. 3a, c). Cumulative probability plots of inter-event intervals confirmed the decrease in mEPSC frequency and revealed a slight change in mEPSC amplitude (Fig. 3a, c). The BAI2 deletion had no impact on mEPSC frequency or amplitude (Fig. 3b), although cumulative probability plots indicated minor changes in mEPSC frequency (Fig. 3b). None of the BAI deletions altered the mean mIPSC frequency or amplitude, suggesting no involvement in inhibitory synaptic transmission correlating with the lack of effect on inhibitory synapse numbers (Fig. 3d–f). Cumulative probability plots of inter-event intervals showed a small change in mIPSC frequency for BAI1 and BAI3 deletions that, owing to the fact that individual events are used as statistical n’s, were statistically significant (Fig. 3d, f). Note, however, that cumulative probability plots of mEPSC and mIPSC inter-event intervals and amplitudes are employed here to detect potential effects of a manipulation on a subset of synapses. The high ‘n’s’ used for statistics in such analyses of mEPSCs and mIPSCs provides a misleading impression of significance. These cumulative probability analyses are generally not suitable for conclusions about the mEPSC frequency and amplitude, for which averages shown in the bar graphs are more appropriate. In our case, the cumulative plots for both the mEPSCs and mIPSCs do not provide tangible evidence for synaptic heterogeneity, suggesting that the BAI deletions uniformly impair excitatory synapses and did not alter a subset of inhibitory synapses that might have been missed in the analysis of averages. Measurements of the membrane capacitance and input resistance, finally, revealed no significant difference between BAI1-, BAI2-, or BAI3-deficient neurons (Supplementary Fig. 5a, b), indicating that BAIs do not regulate non-synaptic electrical properties of neurons.

In summary, these results demonstrate that both BAI1 and BAI3 are essential for regulating three distinct neuronal processes, namely dendritic growth and arborization, axonal growth and arborization, and synapse formation. In contrast, BAI2 does not appear to be required for any of these three processes. Unexpectedly, both BAI1 and BAI3 thus seem to restrict dendritic and axonal growth but to support synapse formation, suggesting non-redundancy. The effect of the BAI1 and BAI3 deletions on excitatory synapse density is not simply a consequence of the increase in dendrite growth. If that had been the case, the inhibitory synapse density should have also been decreased since it was also measured on dendrites. Moreover, in this case the mEPSC frequency should not have been decreased. Thus, BAI1 and BAI3 appear to be truly required for the formation and/or maintenance of excitatory but not inhibitory synapses.

### RTN4R- and C1ql-binding to BAI3 differentially control axonal and dendritic growth and synapse organization

It is intriguing that the deletions of BAI1 and BAI3 in mouse neurons produce the same phenotypes resembling those of RTN4RL1 and RTN4RL2 deletions in human neurons (a loss of excitatory synapses and an increase in axonal and dendritic arborizations)^27^. Since only BAI3 but not BAI1 binds to C1qls that have been shown to be essential for synapse formation in selected connections (Supplementary Fig. 1, 2), the finding that the BAI1 and BAI3 deletions have the same phenotypes seems to suggest that C1ql-binding is not essential for the function of BAIs, a suggestion that is at odds with multiple studies demonstrating that C1qls and BAI3 are central to the formation of at least some synapses^21–23^. To address this conundrum and to determine the relative importance of RTN4R- vs. C1ql-binding to BAIs, we embarked on BAI3 rescue experiments in BAI3-deficient neurons. In these experiments, we focused on BAI3 because it is the only BAI isoform that binds to both RTN4Rs and C1qls and is also the isoform that has been studied most extensively.

**Fig. 4:**
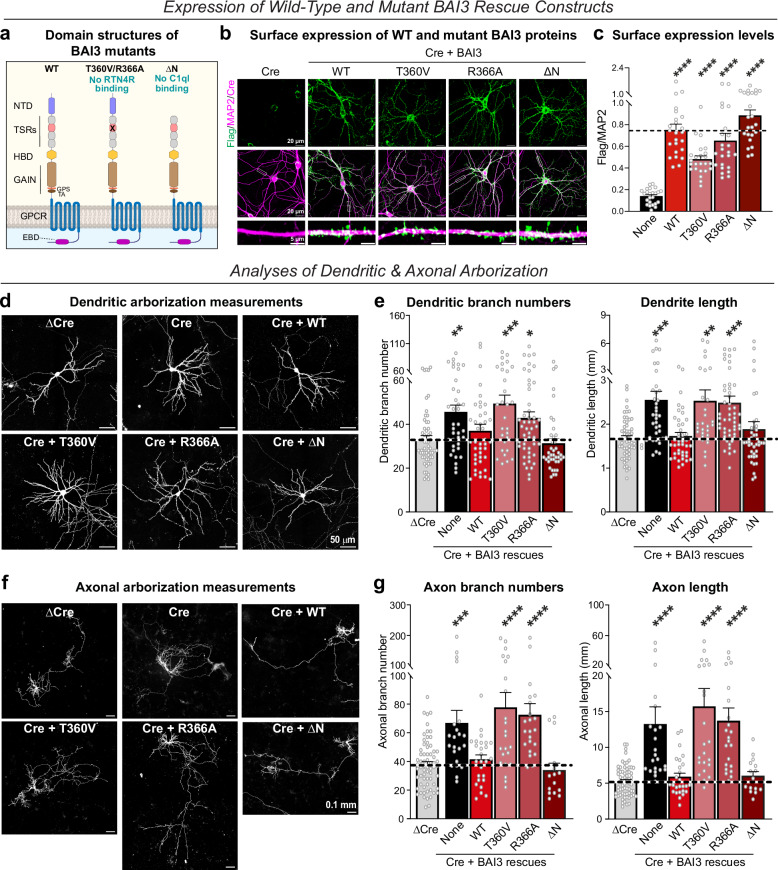
A point mutation blocking RTN4R-binding abolishes the ability of BAI3 to reverse the excessive axonal and dendritic arborizations of BAI3-deficient neurons. **a** Domain structures of wild-type (WT) and mutant BAI3 that lacks binding to RTN4Rs (T360V or R366A) or C1qls (ΔN). Abbreviations used: NTD, N-terminal domain; TSRs, thrombospondin type-1 repeats; HBD, hormone-binding domain; GAIN, GPCR autoproteolysis inducing domain; GPS, GPCR proteolytic site; TA, tethered agonist; EBD, ELMO binding domain. Created in BioRender. Miao, Y. (2025) https://BioRender.com/vi7q8lx. **b** Representative immunocytochemistry images of MAP2 (magenta) and N terminal Flag-tagged wild-type (WT) or mutant BAI3 (Flag, green) in dendritic segments of hippocampal neurons in mixed neuron-glia cultures that were obtained from BAI3 conditional knockout (cKO) mice, infected with lentiviruses expressing Cre at DIV 4 and with lentiviruses encoding the indicated BAI3 constructs at DIV 6, and analyzed at DIV 14. **c** Quantification of the surface expression levels of WT and mutant BAI3 expressed in BAI3-deficient neurons as described in **b** (n’s [cells/experiments] for each column [left to right] = 24/3, 24/3, 24/2, 24/3, and 24/3). (**d** & **e**) Representative images (**d)** and summary graphs of dendritic branch numbers and dendritic length (**e**) of hippocampal neurons that were cultured together with glia from BAI3 cKO mice, infected at DIV 4 with lentiviruses expressing ΔCre or Cre and at DIV 6 with lentiviruses encoding the indicated BAI3 rescue constructs, and that were additionally sparsely transfected with tdTomato at DIV 9 for visualization of individual neurons before analysis at DIV 14 (n’s [cells/experiments] for each column [left to right] = 56/7, 37/7, 42/7, 29/6, 49/7, and 40/6). **f**,**g** Same as (**d**,**e**), except that axons were analyzed (n’s [cells/experiments] for each column [left to right] = 66/16, 22/11, 26/8, 23/10, 22/7, and 15/4). All numerical data are means ± SEM. Dots indicate individual datapoints. Statistical significance was determined for all numerical data using one-way ANOVA followed by Dunnett’s multiple comparison tests (* *p*<0.05, ** *p*<0.01, *** *p*<0.001, **** *p*<0.0001). In (**c**), p = 3.94X10^-19^ for ANOVA; for multiple comparisons, p<0.0001 for None vs. WT, T360V, R366A or ΔN. In (**e**), dendritic branch numbers: for ANOVA, p = 3.18X10^-5^; for multiple comparisons, p = 0.0070 (ΔCre vs. Cre), 0.7994 (ΔCre vs. Cre + BAI3 WT), 0.0007 (ΔCre vs. Cre + BAI3 T360V), 0.0342 (ΔCre vs. Cre + BAI3 R366A) and 0.9849 (ΔCre vs. Cre + BAI3 ΔN). Dendrite length: for ANOVA, p = 8.85X10^-7^; for multiple comparisons, p = 0.0002 (ΔCre vs. Cre), 0.9996 (ΔCre vs. Cre + BAI3 WT), 0.0010 (ΔCre vs. Cre + BAI3 T360V), 0.0002 (ΔCre vs. Cre + BAI3 R366A) and 0.7978 (ΔCre vs. Cre + BAI3 ΔN). In (**g**), axon branch numbers: for ANOVA, p = 4.50X10^-9^; for multiple comparisons, p = 0.0008 (ΔCre vs. Cre), 0.9892 (ΔCre vs. Cre + BAI3 WT), <0.0001 (ΔCre vs. Cre + BAI3 T360V), <0.0001 (ΔCre vs. Cre + BAI3 R366A) and 0.9930 (ΔCre vs. Cre + BAI3 ΔN). Axon length: for ANOVA, p = 6.50X10^-11^; for multiple comparisons, p <0.0001 (ΔCre vs. Cre), = 0.9971 (ΔCre vs. Cre + BAI3 WT), <0.0001 (ΔCre vs. Cre + BAI3 T360V), <0.0001 (ΔCre vs. Cre + BAI3 R366A) and = 0.9968 (ΔCre vs. Cre + BAI3 ΔN).

We generated constructs that encode Flag-tagged wild-type and mutant BAI3 containing either point mutations designed to block RTN4R binding (T360V or R366A)^27^ or a deletion mutation that removes the NTD and was designed to abolish C1ql binding (ΔN)^21^ (Fig. 4a). Cell-aggregation assays confirmed that the BAI3 T360V and R366A mutations ablate RTN4R binding (Supplementary Fig. 6a, b). Moreover, the ΔN mutation abolished C1ql1-4 binding, whereas the BAI3 T360V and R366A mutations had no effect on C1ql1-4 binding as assessed in cell-surface staining assays (Supplementary Fig. 6c–h). Lentiviral expression of the BAI constructs encoding wild-type and mutant BAI3 with extracellular Flag- and intracellular HA-tags in hippocampal cultures showed that all BAI3 proteins were efficiently expressed on the cell surface, with only the BAI3 T360V protein exhibiting slightly lower expression levels than BAI3 WT (Figs. 4b, c). Direct visualization of exogenous BAI3 proteins in astrocytes confirmed, as shown above for Cre, that the lentiviral delivery mediates expression of the BAI3 constructs not only in neurons but also in astrocytes in the mixed neuron-glia cultures (Supplementary Fig. 7a).

To test the role of ligand binding to BAI3, we first explored whether BAI3-dependent regulation of axonal and dendritic growth requires its interaction with RTN4Rs and/or C1qls. We infected hippocampal cultures from BAI3 cKO mice with lentiviruses expressing ΔCre (control) or Cre at DIV4 and with lentiviruses expressing BAI3 rescue proteins at DIV6, and then sparsely transfected the neurons with tdTomato at DIV9 (Supplementary Fig. 7b). Measurements of dendritic and axonal arborizations at DIV14 revealed that both wild-type and ΔN-mutant BAI3 fully reversed the enhanced dendritic and axonal growth of BAI3 KO neurons, whereas T360V- and R366A-mutant BAI3 did not (Fig. 4d–g). None of these manipulations altered the soma area (Supplementary Fig. 7c). Thus, RTN4R binding to BAI3 is indispensable for its function in restricting dendritic and axonal growth in hippocampal neurons, whereas C1ql binding is not required.

**Fig. 5:**
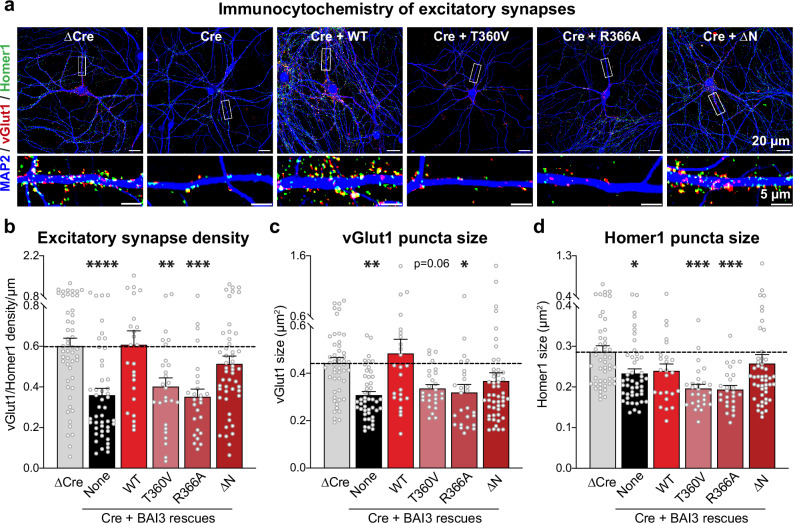
Mutations blocking RTN4R-binding to BAI3 abolish rescue of decreased synapse number in BAI3-deficient neurons whereas the mutation abrogating C1ql3-binding has no effect. **a** Representative immunocytochemistry images of neurons and dendritic segments in mixed neuron-glia cultures from BAI3 conditional knockout (cKO) mice that were infected with lentiviruses expressing ΔCre or Cre at DIV 4 and additionally with lentiviruses encoding the indicated BAI3 constructs at DIV 6, and analyzed by immunocytochemistry at DIV 14 (MAP2 is blue, vGlut1 is red, and Homer1 is green; top images, overview of entire neurons; bottom images, higher magnification of the area marked by a white rectangle in the upper images). **b–d** Quantifications of excitatory synapse density (**b**), vGlut1 puncta size (**c**), and Homer1 puncta size (**d**) for experiments in (**a**). Puncta positive for both vGlut1 (red) and Homer1 (green) are defined as validated synapses. Data are means ± SEM (n’s [cells/experiments] for each column [left to right] = 50/6, 47/6, 24/3, 27/3, 26/3, and 49/6). Statistical significance was determined with one-way ANOVA followed by Dunnett’s multiple comparison tests (* p<0.05, ** p<0.01, *** p<0.001, **** p<0.0001). In (**b**), p = 1.39X10^-6^ for ANOVA; for multiple comparisons, p<0.0001 (ΔCre vs. Cre), >0.9999 (ΔCre vs. Cre + BAI3 WT), = 0.0058 (ΔCre vs. Cre + BAI3 T360V), = 0.0003 (ΔCre vs. Cre + BAI3 R366A) and = 0.3224 (ΔCre vs. Cre + BAI3 ΔN). In (**c**), p = 1.72X10^-4^ for ANOVA; for multiple comparisons, p = 0.0015 (ΔCre vs. Cre), 0.8669 (ΔCre vs. Cre + BAI3 WT), 0.0613 (ΔCre vs. Cre + BAI3 T360V), 0.0233 (ΔCre vs. Cre + BAI3 R366A) and 0.1580 (ΔCre vs. Cre + BAI3 ΔN). In (**d**), p = 4.94X10^-4^ for ANOVA; for multiple comparisons, p = 0.0405 (ΔCre vs. Cre), 0.2303 (ΔCre vs. Cre + BAI3 WT), 0.0010 (ΔCre vs. Cre + BAI3 T360V), 0.0008 (ΔCre vs. Cre + BAI3 R366A) and 0.4866 (ΔCre vs. Cre + BAI3 ΔN).

To determine which BAI3-mediated interactions are essential for its role in synapse formation, we employed the same rescue strategy as in the analysis of neuronal development, except that instead of sparsely transfecting neurons with tdTomato at DIV9, we immunolabeled the neurons at DIV14 for the synaptic markers vGluT1 and Homer1 (to visualize excitatory synapses) (Supplementary Fig. 7d). We then tested the ability of these rescue constructs to reverse the decrease in synapse density caused by the BAI3 deletion. Quantifications of excitatory synapses, measured as puncta with both a presynaptic vGluT1 and a postsynaptic Homer1 signal, revealed that wild-type BAI3 fully rescued the ~40% decrease in synapse density produced by the BAI3 deletion, whereas T360V- and R366A-mutant BAI3 were unable to reverse the decrease in synapse density (Fig. 5a, b). Moreover, the ΔN-mutant BAI3 that is unable to bind to C1qls also rescued the decrease in synapse density (Fig. 5a, b). Synaptic puncta size measurements produced the same results. T360V- and R366A-mutant BAI3 failed to rescue the decrease in apparent synapse size in contrast to wild-type and ΔN-mutant BAI3, which rescued this decrease (Fig. 5c, d).

**Fig. 6:**
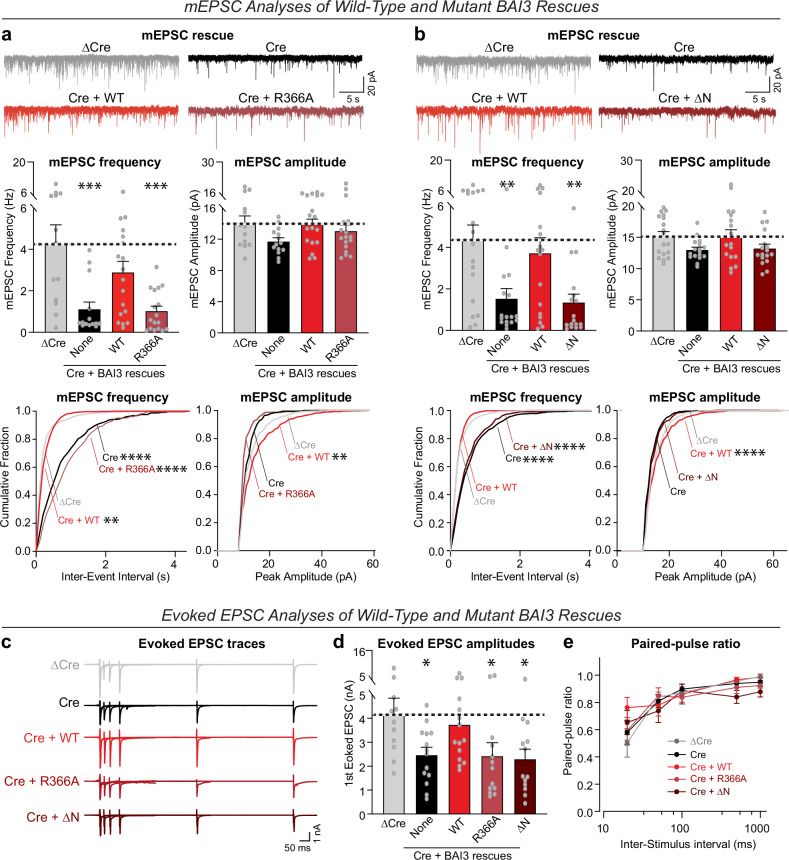
Mutations blocking RTN4R-binding or C1ql3-binding by BAI3 similarly prevent rescue of impaired synaptic transmission in BAI3-deficient neurons. **a** Representative traces (top), summary graphs of the frequency and amplitude (middle), and cumulative fraction plots of the inter-event interval and peak amplitude (bottom) of miniature excitatory postsynaptic currents (mEPSCs) recorded from hippocampal neurons in mixed neuron-glia cultures obtained from BAI3 conditional knockout (cKO) mice. Cultures were infected with lentiviruses expressing ΔCre or Cre at DIV4 and additionally with lentiviruses encoding the wild-type (WT) or R366A-mutant BAI3 constructs at DIV6, and analyzed by patch-clamp recordings at DIV14 (n [cells/experiments] = 13/4, 14/4, 18/4, and 17/4 for the ΔCre, Cre only, Cre + WT rescue, and Cre + R366A-mutant rescue, respectively). For mEPSC frequency bar graph, p = 0.0004, 0.1453, 0.0001 from left to right; for mEPSC amplitude bar graph, p = 0.0849, 0.9979, 0.6337 from left to right. For mEPSC frequency cumulative fraction, ΔCre vs. Cre, p<0.0001; ΔCre vs. Cre + BAI3 WT, p = 0.0068; ΔCre vs. Cre + BAI3 R366A, p<0.0001. For mEPSC amplitude cumulative fraction, ΔCre vs. Cre, p = 0.2567; ΔCre vs. Cre + BAI3 WT, p=0.0082; ΔCre vs. Cre + BAI3 R366A, p = 0.1741. **b** Same as (**a**), except that cultures were rescued with the C1ql-binding mutant ΔN instead of the RTN4R-binding mutant R366A (n [cells/experiments] = 18/5, 17/5, 17/5, and 17/5 for the ΔCre, Cre only, Cre + WT rescue, and Cre + ΔN-mutant rescue, respectively). For mEPSC frequency bar graph, p = 0.0040, 0.7862, 0.0020 from left to right; for mEPSC amplitude bar graph, p = 0.1093, >0.9999, = 0.1751 from left to right. For mEPSC frequency cumulative fraction, ΔCre vs. Cre, p<0.0001; ΔCre vs. Cre + BAI3 WT, p = 0.2700; ΔCre vs. Cre+ BAI3 ΔN, p<0.0001. For mEPSC amplitude cumulative fraction, ΔCre vs. Cre, p = 0.0891; ΔCre vs. Cre + BAI3 WT, p<0.0001; ΔCre vs. Cre+ BAI3 ΔN, p=0.0562. **c** Representative traces of evoked excitatory postsynaptic currents (EPSCs) elicited by closely spaced pairs of action potentials (intervals: 20, 50, 100, 500 and 1000 ms) from BAI3 cKO mouse neurons infected with the indicated lentiviruses as described for (**a**) and (**b**). **d** Quantification of the amplitude of the first evoked EPSC during paired-pulse measurements (n [cells/experiments] = 11/4, 14/4, 15/4, 13/4, and 13/4 for the ΔCre, Cre only, Cre + WT rescue, Cre + R366A-mutant, and Cre + ΔN-mutant rescue, respectively). P = 0.0473, 0.8929, 0.0442 and 0.0287 from left to right. **e** Summary plot of the paired-pulse ratios (PPRs) measured as the amplitude ratio of the second over the first evoked EPSC and plotted as a function of the inter-stimulus interval. n [cells/experiments] = 11/4, 14/4, 15/4, 13/4, and 13/4 (interval time, 20 ms); 11/4, 14/4, 15/4, 13/4, and 13/4 (interval time, 50 ms); 10/4, 12/4, 13/4, 12/4, and 13/4 (interval time, 100 ms); 10/4, 10/4, 12/4, 9/4, and 12/4 (interval time, 500 ms); 10/4, 9/4, 12/4, 9/4, and 9/4 (interval time 1000 ms) for ΔCre, Cre only, Cre + WT rescue, Cre + R366A-mutant, and Cre + ΔN-mutant rescue, respectively. p = 0.9591, 0.8285, 0.8957 and 0.4850 from top to bottom. All numerical data are means ± SEM. Dots indicate individual datapoints. Statistical significance was determined by one-way ANOVA followed by Dunnett’s multiple comparison tests (bar graphs in **a**, **b** and **d**), the two-tailed Kolmogorov–Smirnov test at a 95% confidence level (cumulative distributions in (**a**,**b**), or two-way ANOVA (summary plot in **e**) (n.s., not significant, * p<0.05, ** p<0.01, *** p<0.001, **** p<0.0001).

Given that extensive evidence documents an essential role for C1ql-binding to BAI3 in synapse organization^21–23^, it was puzzling to us that the C1ql-binding deficient ΔN-mutant BAI3 appeared to reverse not only the exuberant dendritic and axonal arborization caused by the BAI3 deletion (Fig. 4), but also the decrease in synapse density produced by the BAI3 deletion (Fig. 5a, b). To determine whether C1ql-binding by BAI3 may be selectively essential for synapse function as opposed to the development of neurons, we analyzed the ability of various BAI3 mutants to rescue the electrophysiological phenotype of BAI3-deficient neurons. Whole-cell patch-clamp recordings of mEPSCs revealed that, as expected from the morphological measurements, the R366A mutation of BAI3 that blocks RTN4R binding failed to rescue the large decline in mEPSC frequency (>70% decrease) caused by the BAI3 deletion (Fig. 6a). Gratifyingly in view of previous studies^21–23,28^, the ΔN-mutant BAI3 that blocks C1ql3 binding also failed to rescue this decrease in mEPSC frequency (Fig. 6b). Cumulative probability plots of the inter-event intervals confirmed the reduction in mEPSC frequency and indicated that the R366A and ΔN mutants did not rescue this phenotype (Fig. 6a, b). Moreover, measurements of evoked EPSCs, performed using paired-pulse stimulations to also monitor the release probability, demonstrated that the BAI3 deletion caused a decrease in synaptic strength that was reversed by wild-type BAI3 expression but was also not rescued by either the RTN4R- or the C1ql-binding-deficient BAI3 mutants (Fig. 6c, d). No change in paired-pulse ratios was detected (Fig. 6e), suggesting that the BAI3 deletion and the various rescue manipulations did not alter the release probability. In addition, no changes in the passive electrical properties of neurons were observed as a function of the BAI3 deletion and rescue manipulations (Supplementary Fig. 8). Thus, both RTN4R- and C1ql-binding to BAI3 are required for synapse organization, although only the former appears to be essential for neuronal development and synapse formation as such.

**Fig. 7:**
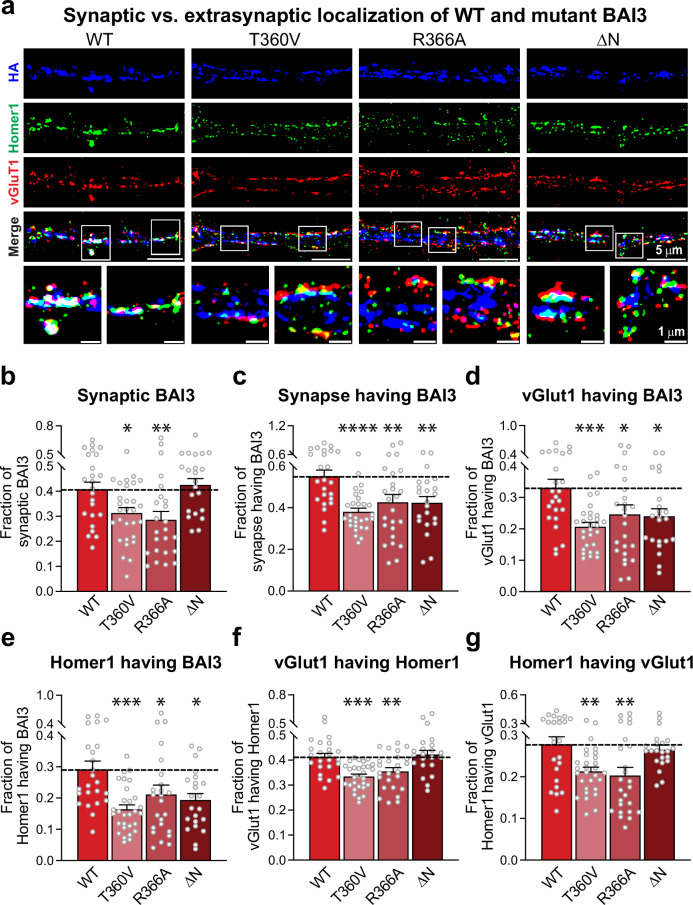
Both wild-type and all mutant BAI3 proteins are partly localized to synapses. **a** Representative STED microscopy images of immunocytochemically localized HA-tagged BAI3 (blue), Homer1 (green) and vGlut1 (red) in BAI3-deficient hippocampal neuron-glia cultures that lentivirally express the indicated proteins. The bottom panel displays magnified views of the white rectangles in the merged images. WT: wild-type. **b** Quantification of the fraction of synaptic BAI3 proteins, defined as HA-signals co-localized with synapses (co-localized vGlut1 and Homer1) for experiments in (**a**). **c–e** Quantification of the fraction of synapses (**c**, synapses are defined as co-localized vGlut1 and Homer1), vGlut1 (**d**) or Homer1 (**e**) having BAI3 proteins for experiments in (**a**). **f**, **g** Quantification of the fraction of vGlut1 having Homer1 (**f**) and Homer1 having vGlut1 (**g**) for experiments in (**a**). All numerical data are means ± SEM. Dots indicate individual datapoints (n’s [cells/experiments] for each column [left to right] = 24/3, 29/3, 24/3, and 22/3). Statistical significance was determined for all numerical data using one-way ANOVA followed by Dunnett’s multiple comparison tests (* p<0.05, ** p<0.01, *** p<0.001, **** p<0.0001). In (**b**), p = 0.0005 for ANOVA; for multiple comparisons, p = 0.0287 (WT vs. T360V), 0.0053 (WT vs. R366A) and 0.9454 (WT vs. ΔN). In (**c**), p = 0.0003 for ANOVA; for multiple comparisons, p<0.0001 (WT vs. T360V), = 0.0072 (WT vs. R366A) and 0.0085 (WT vs. ΔN). In (**d**), p = 0.0028 for ANOVA; for multiple comparisons, p = 0.0008 (WT vs. T360V), 0.0376 (WT vs. R366A) and 0.0302 (WT vs. ΔN). In (**e**), p = 0.0010 for ANOVA; for multiple comparisons, p = 0.0003 (WT vs. T360V), 0.0417 (WT vs. R366A) and 0.0116 (WT vs. ΔN). In (**f**), p = 5.72X10^-6^ for ANOVA; for multiple comparisons, p = 0.0002 (WT vs. T360V), 0.0088 (WT vs. R366A) and 0.9411 (WT vs. ΔN). In (**g**), p = 0.0007 for ANOVA; for multiple comparisons, p = 0.0058 (WT vs. T360V), 0.0019 (WT vs. R366A) and 0.8899 (WT vs. ΔN).

A key question emerging from these functional analyses of RTN4R- and C1ql-binding to BAI3 is whether the various BAI3 mutants localize to synapses and whether they might affect the structure of synapses. To address this question, we examined excitatory synapses by STED super-resolution microscopy in BAI3-deficient hippocampal neurons expressing wild-type or mutant BAI3 (Fig. 7; Supplementary Fig. 9). More than 50% of synapses contained BAI3, indicating that a significant fraction of BAI3, when exogenously expressed in neurons, localizes to synapses (Fig. 7a–c). The three mutations of BAI3 modestly but significantly decreased the synaptic localization of BAI3 independent of whether the synaptic localization was measured via Homer1 or vGluT1 co-localization, suggesting that the mutations slightly impaired the efficiency of synaptic targeting (Fig. 7c–e). The co-localization of vGluT1 and Homer1 signals in synapses was decreased by the RTN4R-binding mutations but not by the C1ql-binding mutation (Fig. 7f, g), as was the fraction of BAI3 that localized to synapses (Fig. 7b). These results suggest that although all BAI3 mutants, like wild-type BAI3, are still targeted to synapses, only the RTN4R-binding mutants likely affect the architecture of synapses, a conclusion that is consistent with the observation that only the loss of RTN4R-binding has a major effect on synapse density (Fig. 5), whereas the loss of C1ql-binding appears to primarily impair synapse function (Fig. 6).

### The cell-type specificity of the expression of BAIs, RTN4Rs, and C1qls corresponds to the synaptic knockout phenotypes

**Fig. 8:**
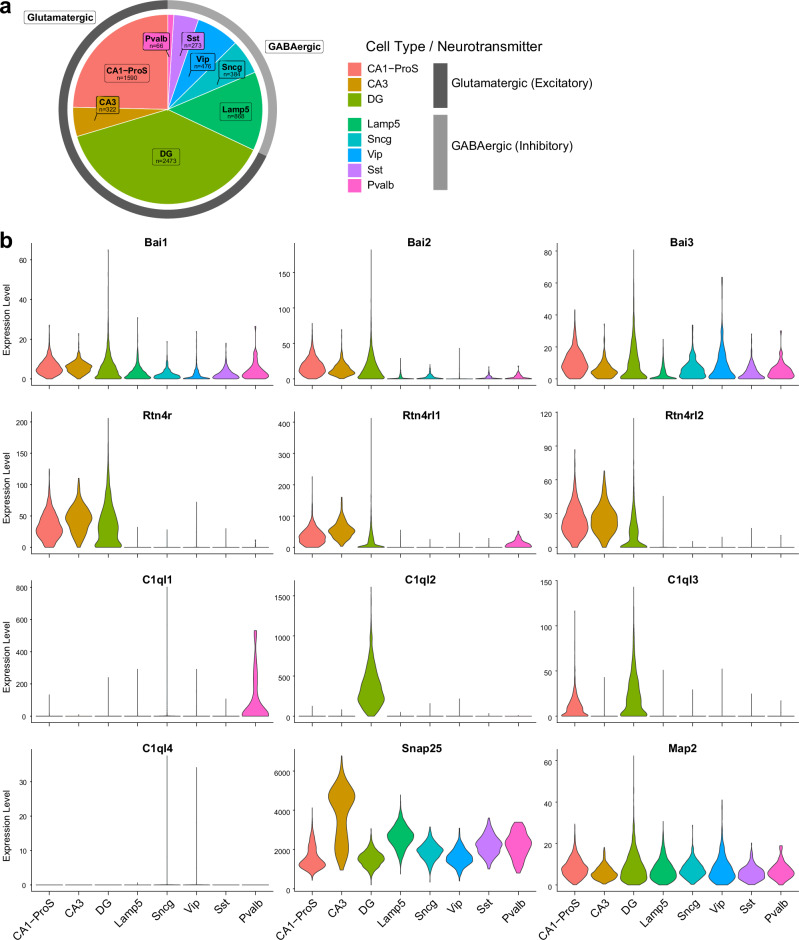
Expression profiles of *Bai1-3* (*Adgrb1-3*), *Rtn4rs* (*Rtn4r, Rtn4rl1, Rtn4rl2*), *C1qls* (*C1ql1–4*) genes across hippocampal cell types. **a** Pie chart illustrating the abundance of different cell types within the hippocampal dataset analyzed. Data were from the Allen Brain Institute^44^. **b** Violin plots showing the expression levels of *Bai1 (Adgrb1)*, *Bai2 (Adgrb2)*, *Bai3 (Adgrb3)*, *Rtn4r*, *Rtn4rl1*, *Rtn4rl2*, *C1ql1–4*, *Snap25*, and *Map2* in distinct hippocampal cell types.

The results described above raise at least two major questions. First, both the systematic comparison of the BAI1-3 deletions and the BAI3 rescue experiments indicate that in hippocampal neuron-glia cultures, the three major phenotypes observed in BAI1 and BAI3 KO neurons reflect an RTN4R-dependent function. As mentioned above, however, numerous studies have confirmed a key role for C1ql-binding to BAI3 in synapse formation^21–23,28^, raising the question why such a major role for C1ql-binding to BAI3 was not detected in our rescue experiments. Second, the BAI1 and BAI3 deletions selectively impair only excitatory synapses and not inhibitory synapses but the experiments we performed provide no hypothesis for explaining this specificity. As a first step to addressing these questions, we analyzed deep single-cell transcriptomics data from the mouse hippocampus that were generated by the Allen Brain Institute^44^ (Fig. 8).

We found that *Bai1* and *Bai3* were broadly expressed in all neurons at approximately half the abundance of *Map2* and are thus abundant (Fig. 8a, b). *Bai2*, conversely, was expressed at even higher levels in excitatory neurons at approximately twice the abundance of *Map2*, but at much lower levels in inhibitory neurons (Fig. 8b). Thus, most excitatory neurons co-express all three BAI isoforms, whereas inhibitory neurons generally co-express *Bai1* and *Bai3*.

In contrast to *Bais*, *Rtn4rs* were almost exclusively expressed only in excitatory neurons, where high levels were observed, but not in inhibitory neurons, where *Rtn4rs* were undetectable (Fig. 8b). The only exception was *Rtn4rl1* that exhibited low-level expression in parvalbumin-positive interneurons in addition to high-level expression in excitatory neurons. Notably, expression levels of *Rtn4rs* were 2-4 fold higher than those of *Map2* and Rtn4rs are thus abundant. The expression pattern of presynaptic *Rtn4rs* correlates with the specificity of BAI function for excitatory synapse formation, suggesting that it accounts for this specificity. The low levels of Rtn4rl1 in parvalbumin-positive interneurons are likely negligible for our experiments because these interneurons represent only a minor component of all inhibitory neurons (Fig. 8a).

Different from both *Bais* and *Rtn4rs*, *C1qls* exhibited a restricted expression pattern (Fig. 8b). *C1ql1* was only detectable in parvalbumin-positive interneurons. *C1ql2* was selectively observed in dentate gyrus granule cells as described previously^29^, where it was expressed at very high levels (>20-fold higher than *Map2*) (Fig. 8b). *C1ql3* was present at lower levels in both dentate gyrus granule cells and in CA1 pyramidal neurons, and *C1ql4* was undetectable (Fig. 8b). The *C1ql* expression pattern fits well with previous results revealing a selective role for C1ql2 and C1ql3 in dentate gyrus→CA3 synapses^28,29^. However, the data also demonstrate that the majority of excitatory neurons in the hippocampus express a C1ql isoform since only CA3 neurons do not. Even if C1qls were not secreted, most synapses in our primary cultures would thus be exposed to a C1ql protein, with the most populous dentate gyrus granule cells producing much higher levels than those of RTN4Rs.

## Discussion

**Fig. 9:**
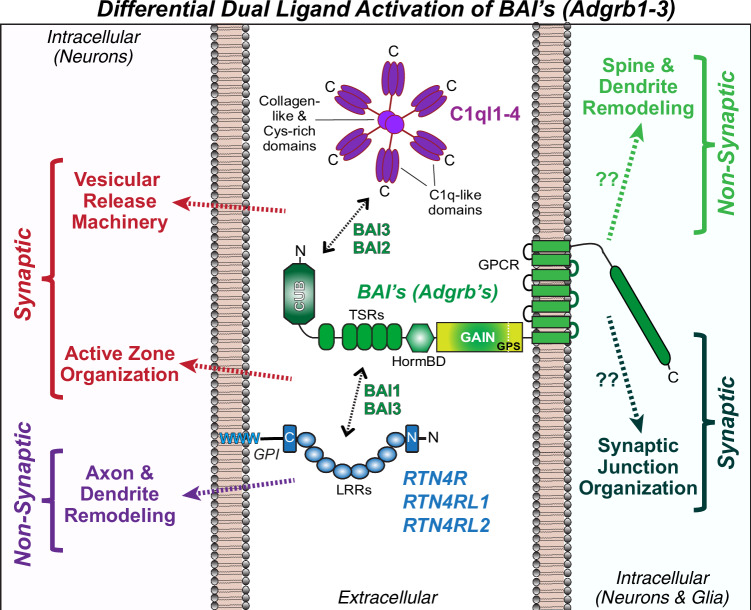
Summary cartoon of the control of BAI functions by binding of RTN4R’s and C1ql’s. BAI’s exhibit distinct ligand-binding patterns such that BAI3 binds to both RTN4R’s and C1ql’s, BAI1 only binds to RTN4R’s, and BAI2 only to C1ql’s. BAI1 and BAI3 both act to restrict axonal and dendritic growth and to enhance synapse formation by binding to RTN4R’s as shown here in mouse neurons and suggested by previous studies^2,11–13,21–24,27^. BAI3 additionally regulates synapse organization by binding to C1ql’s that form an 18-meric assembly which can bind to, and potentially cluster, up to 18 BAI3 molecules. We found BAI2 has no essential functions in axonal or dendritic arborization or in synapse organization but likely performs other important roles^19,20^ (abbreviations used: TSRs, thrombospondin repeats; LRRs, leucine-rich repeats; GPI, glycosylphosphatidylinositol anchor; HormBD: hormone-binding domain; GAIN, GPCR-autoproteolysis inducing domain; GPS, GPCR proteolysis sequence; CUB, complement C1r/C1s, Uegf, Bmp1).

Extensive studies document an important role for BAI proteins in brain^1–3^ but their relative functions and mechanisms of action remain unclear (see Introduction). To gain a comprehensive understanding of the relative functions of BAIs in neural development, we here systematically compared the functional properties of BAI1-3. We show that BAI1 and BAI3 are multifaceted signaling molecules that are individually essential for at least three basic processes in neural development, namely the control of axonal and dendritic growth and the formation of synapses. BAI2, in contrast, is dispensable for these three processes, suggesting a different biological role. We then focused on BAI3 because we found that it is the only BAI isoform that binds to both RTN4Rs and C1qls, leading us to analyze the relation of its biochemical binding properties to its mechanism of action in neuron-glia cultures as a reduced system. Our results reveal that the BAI3-mediated regulation of axonal and dendritic arborizations and of synapse formation require RTN4R-binding, whereas only its action in enabling normal synaptic transmission additionally requires C1ql-binding (Fig. 9). Our data position BAI1 and BAI3 into a central role in neuronal development, a role that requires RTN4R binding and is likely active throughout life given the continuous remodeling of neural circuits and the sustained expression of BAIs in adult mice, while BAI3 additionally tunes the performance of synapses by binding to C1qls.

### Specifically, our data enable five conclusions supported by the following evidence

First, BAI2 and BAI3 but not BAI1 bind to C1qls as evidenced by size exclusion chromatography of their complexes (Supplementary Fig. 1), which confirms prior structural data^42^. In contrast, as previously shown^27^, BAI1 and BAI3 but not BAI2 bind to RTN4Rs. Thus, each of the three BAI isoforms has a distinct ligand-binding profile with BAI1 binding only RTN4Rs, BAI2 binding only C1qls (albeit weakly), and BAI3 binding both.

Second, our analyses of BAI deletions in mixed mouse neuron-glia cultures demonstrate nearly identical phenotypes for the BAI1 and BAI3 deletions that consists of a large increase in dendritic and axonal growth (Fig. 1), a decrease in excitatory synapse numbers (Fig. 2), and a suppression of spontaneous excitatory synaptic events (Fig. 3). Inhibitory synapses were unaffected, consistent with the lack of expression of *RTN4Rs* in hippocampal inhibitory neurons (Fig. 8). Moreover, only the BAI3 deletion additionally caused a significant decrease in synapse size (Fig. 2). Different from the BAI1 and BAI3 deletions, the BAI2 deletion exhibited no impairment in any of these parameters, suggesting that BAI2 is functionally distinct from BAI1 and BAI3.

Third, in agreement with a synaptic function for BAI3, a significant portion of excitatory synapses (>50%) contain exogenously expressed BAI3 as monitored by STED super-resolution microscopy, with a large fraction of the exogenously expressed BAI3 (~40%) recruited to synapses (Fig. 7).

Fourth, rescue experiments demonstrated that BAI3 controls axonal and dendritic growth and supports synapse formation by binding to RTN4Rs, consistent with the fact that deletions of BAI1 and BAI3 (which both bind to RTN4Rs) produce nearly identical phenotypes in these parameters (Figs. 1–6).

Fifth, C1ql-binding to BAI3 in hippocampal neuron-glia cultures is required for the normal organization of synapses even though it is not essential for the dendritic and axonal development of neurons (Figs. 4–6). The importance of this role of C1ql-binding to BAI3 is highlighted by the previously characterized in vivo phenotypes of C1ql deletions^21,28^. However, the impact of C1ql-binding to BAI3 seems to be relatively lower than that of RTN4Rs, a finding that cannot be explained by the selective expression patterns of *C1qls* since most excitatory hippocampal neurons express a *C1ql* isoform, with the most populous dentate gyrus granule cells transcribing very high levels of the *C1ql2* gene (Fig. 8).

Viewed together, these findings characterize BAI1 and BAI3 as key agents in regulating neuronal development in a manner that is primarily controlled by binding of RTN4Rs. A plausible hypothesis is that the BAI1 and BAI3 GPCR activity regulates signaling cascades controlling axon and dendrite growth and synapse formation, such that the BAI1 and BAI3 activity serves as a ‘stop-growth-and-induce-synapse-formation’ message. It is gratifying in this regard that the phenotypes of the deletions of RTN4Rs^27,40^ and of BAI1 and BAI3 (Figs. 1–3) are similar, suggesting an active contribution of the RTN4R-BAI complex to regulating axonal and dendritic arborizations and synapse formation. In contrast to these conclusions about RTN4R-binding to BAIs that are enabled by our experiments, our experiments using a reduced culture system allow only a limited assessment of the role of C1ql-binding to BAI3. It is likely that the function of C1ql-binding to BAI3 is superseded by that of RTN4R binding.

Several limitations of our study should be noted, starting with the fact that we performed our functional analyses only in hippocampal cultures, a reduced system distinct from an intact brain, and examined only axonal and dendritic arborizations and synapse formation. Our results therefore do not exclude the possibility of other functions for BAI1 and BAI3 besides those described here in the context of the cytoarchitecture of an intact hippocampus, nor do our results mean that BAI2 has no function as discussed below. This limitation in particular applies to C1ql-binding to BAI3. Although we found little functional relevance of C1ql-binding to BAI3 in culture, it is entirely possible, maybe even likely, that in the context of a physiological neural circuit the importance of this binding is much more impactful.

Another limitation of our study is that we did not distinguish between glial (presumably astrocytic) and neuronal BAIs. Although we used the human synapsin promoter in our experiments to drive Cre recombinase expression, this promoter mediates expression not only in neurons but also in glia, albeit at lower levels (Supplementary Fig. 4a, 4b). Moreover, our experiments did not probe the mechanisms of BAI1 and BAI3 function beyond determining the relative importance of their RTN4R- and C1ql-binding activities. Since RTN4Rs are GPI-anchored proteins, it seems likely that they interact with additional ligands to regulate axonal and dendritic arborizations and synapse formation. Similarly, it is probable that C1qls as secreted proteins bind to additional receptors, analogous to the simultaneous interaction of cerebellins with neurexins and GluDs (reviewed in ref. ^45^).

Our study also raises new questions, among which three may be particularly important. First, why are BAI1 and BAI3 not functionally redundant if they operate by the same RTN4R-mediated mechanism? It is possible that BAI1 and BAI3 protein levels are rate-limiting, which might account for this lack of redundancy, or that BAI1 and BAI3 operate in different neurons or perform distinct functions in the same neurons. Notably, we observed the same non-redundancy for the BAI1/3 ligands RTN4RL1 and RTN4RL2 in human neurons^27^, suggesting that BAI1 and BAI3 as well as RTN4RL1 and RTN4RL2 protein levels may indeed be rate-limiting.

Second, what is the function of BAI2 since previous studies of BAI2-mutant mice uncovered major brain-related phenotypes^19,20^? Our experimental approach was purposely limited to a few study parameters in cultured hippocampal neurons in order to be able to systematically examine all three BAI isoforms and study the molecular mechanisms. If BAI2 performs functions in brain that differ from the functions analyzed here, such functions may rely on C1ql-binding or involve another ligand for the BAI2 NTD or TSRs that remains to be identified.

Third, we find that C1ql binding to BAI3 is essential for organizing synapses to enable full synaptic transmission but that BAI1 is also essential for synapse organization without binding to C1qls. How does BAI1 work in synapse organization without binding to C1qls? Possibly binding of RTN4R and another unknown effector to the BAI1 NTD is involved, or a baseline level of synaptic transmission is achieved without C1ql-binding.

Fourth, we detected only modest impairments in BAI3 function upon deletion of the C1ql-binding domain in the BAI3 ΔN mutant. However, compelling studies using in vivo manipulations demonstrated that this binding is essential for BAI3’s function in synapse formation^21–24^. At least two hypotheses may explain this discrepancy. It is possible that the functional effect of the BAI3 ΔN mutation we observed could lead to a secondary synapse destabilization in vivo given the much longer timeframe with which the BAI3 and C1ql deletions operate in vivo, and thereby cause synapse loss. Alternatively, it is conceivable that in vivo additional molecules that are absent from dissociated cultures are modulating synapse formation and render it C1ql-dependent.

In conclusion, our research reveals that BAI1 and BAI3 both promote synapse formation and restrict axonal and dendritic arborizations during neuronal development by binding to RTN4Rs, and that in addition, BAI3, but not BAI1, binds to C1qls, which serves as an essential enhancer of synaptic transmission. Our study thus exemplifies how different ligands of an adhesion-GPCR can contribute to various physiological functions, offering insights into the broader biological significance of adhesion-GPCRs.

***Note added at Proof***: Further references to papers published after the final version of this paper was submitted^46–51^.

## Methods

### Vertebrate animals

The research complies with all relevant ethical regulations. All animal usage was approved by Stanford Institutional Animal Care and Use Committee (IACUC), Administrative Panel on Laboratory Animal Care (APLAC) Research Compliance Office, Stanford University and followed NIH Guidelines for the Care and Use of Laboratory Animals. The animal protocol number is 18846. Euthanasia was performed using carbon dioxide inhalation, followed by double confirmation of death through cervical dislocation. B6.C-Tg(CMV-cre)1Cgn/J (006054) and C57BL/6J (000664) mice were purchased from the Jackson Laboratory. CD1 IGS mice were purchased from Charles River Laboratories. BAI2 and BAI3 conditional knockout (cKO) mice were generously provided by Dr. Michisuke Yuzaki at Keio University^21^. BAI1 knockin and cKO mice were generated at Janelia Research Campus^27^.

### Cell lines

Expi293F^TM^ cells (Thermo Fisher Scientific) were cultured using Expi293 Expression Medium (Thermo Fisher Scientific). FreeStyle 293-F cells (Thermo Fisher Scientific) were grown in FreeStyle 293 Expression Medium (Thermo Fisher Scientific). Cells were cultured at 37 °C, with humidified atmosphere of 8% CO_2_ on a shaker at 130 rpm. Lenti-X 293T cells (Takara) and HEK 293T cells (ATCC) were cultured in Dulbecco’s Modified Eagle Medium (Thermo Fisher Scientific) supplemented with 10% FBS, at 37°C, with humidified atmosphere of 5% CO_2_. These cells were thawed from a fresh vial every month and therefore not checked for mycoplasma contamination.

### BAI1 KO, BAI2 cKO and BAI3 cKO mice

To generate BAI1 constitutive KO mice, BAI1 knockin and cKO male mice^27^ were crossed with CMV-Cre (B6.C-Tg(CMV-cre)1Cgn/J, 006054) female mice to delete BAI1 in all tissues^43^. The Cre was further removed by crossing the offspring with C57BL/6J wild-type mice, followed by genotyping validation. Below are the genotyping primers for BAI mice. BAI2 and BAI3 cKO mice were generously provided by Dr. Michisuke Yuzaki at Keio University^21^, and further crossed with CD1 IGS wild-type mice for better breeding purpose.

BAI1 set 1 forward: GTGGAATCCAGAGAACCTTG

BAI1 set 1 reverse: GAAACTGGCTACGGATCCTT

BAI1 set 2 forward: TACTGTGCAAAGCCTCATGCCTCAC

BAI1 set 2 reverse: GAGACAGTGGAAGCAGCGATAACTCC

BAI1 set 3 forward: TACTGTGCAAAGCCTCATGCCTCAC

BAI1 set 3 reverse: GAAACTGGCTACGGATCCTT

Cre set 1 forward: GAACC TGATG GACAT GTTCA GG

Cre set 1 reverse: AGTGC GTTCG AACGC TAGAG CCTGT

Cre set 2 forward: GCCAGCTAAACATGCTTCATC

Cre set 2 reverse: ATTGCCCCTGTTTCACTATCC

BAI2 set 1 forward: GTATAGCTGCCAGCAGTCAATGG

BAI2 set 1 reverse: CATTCTAGCCACTGGCCTTCCAC

BAI2 set 2 forward: AGATGTGCAGGGATGAGTACG

BAI2 set 2 reverse: GGGAACTAGGGACTCGAAGATAAC

BAI3 set 1 forward: TCTCAGTCTTCATGGAGTGG

BAI3 set 1 reverse: TCTGTGGTGCAAGGAACCAT

BAI3 set 2 forward: AAAGCAAGGAGGCGTATCCAG

BAI3 set 2 reverse: GGCAGGATGTGCTAAGCAAATG

### DNA constructs

Lentiviral plasmids FSW-hSyn-NLS-EGFP-Cre and FSW-hSyn-NLS-EGFP-ΔCre with nuclear localization signal (NLS) were the same as previously used^52^. On basis of these two constructs, FSW-hSyn-NLS-tdTomato-Cre and FSW-hSyn-NLS-tdTomato-ΔCre plasmids were generated by replacing the EGFP sequence with tdTomato sequence. For all the rescue experiments, wild-type BAI3 (NM_175642.4), BAI3 T360V, BAI3 R366A or BAI3 ΔN (1-290 aa deleted) cDNAs were inserted into the same FSW-hSyn vector, with the original signal peptide replaced by a preprotrypsin leader signal peptide. A Flag tag was inserted after the signal peptide, and an HA tag was inserted before the PDZ-binding motif (PLDVQEGDFQTEV) in the C terminus. Lentiviral helper plasmids (pRSV-REV, pMDLg/pRRE and vesicular stomatitis virus G protein (VSV-G)) were the same as previously used^27^. The BAI extracellular domain constructs were cloned into pEGBacmam vector after signal peptide and C-terminal Avi (GLNDIFEAQKIEWHE) and His-tags^27^. C1ql1 (125-258 aa) (Uniprot ID: O88992), C1ql2 (154-287 aa) (Uniprot ID: Q8CFR0), C1ql3 (122-255 aa) (Uniprto ID: Q9ESN4) and C1ql4 (105-238 aa) (Uniprto ID: Q4ZJM9) cDNAs were cloned into pEGBacmam vector with IgK signal peptide and HA tag in the N terminus, and 6 X His tags in the N terminus. The pCAG empty vector, pCAG-BAI1, pCAG-BAI2, pCAG-BAI3, pCMV5-RTN4R, pCMV5 empty vector, pCMV5-GFP, and pCMV5-mCherry were used^27^. Expression plasmids for pCAG-BAI3 T360V, BAI3 R366A, and BAI3 ΔN were generated by replacing the BAI3 WT sequence with the respective cDNAs. The original signal peptide of all BAI3 WT and mutant sequences was replaced by a preprotrypsin leader signal peptide followed by a Flag tag. The pCAG-tdTomato plasmid was a gift from Angelique Bordey (Addgene #83029).

### Antibodies

These antibodies were used for confocal microscopy imaging at the indicated dilution ratios: vGlut1 (guinea pig, Millipore, AB5905, 1:1000), Homer1 (rabbit, Millipore, ABN37, 1:500) and MAP2 (chicken, Encor, #CPCA-MAP2, 1:1000), vGAT (guinea pig, Synaptic Systems, 131004, 1:1000), gephyrin (mouse, Synaptic Systems, 147111, 1:500), HA (mouse, Covance, MMS101R, 1:1000), GFAP (mouse, Millipore, MAB360, 1:1000), HA (rabbit, Cell Signaling Technologies, 3724, 1:1000), FLAG (mouse, Sigma, F3165, 1:500), FLAG (rabbit, Sigma, F7425, 1:500), NeuN (rabbit, Millipore, ABN78, 1:1000), and GFP (chicken, Aves Labs, GFP-1020, 1:1000). Goat anti-Guinea Pig IgG (H+L) Secondary Antibody, Alexa Fluor™ 546 (Thermo Fisher Scientific, A-11074, 1:1500), Goat anti-Guinea Pig IgG (H+L) Secondary Antibody, Alexa Fluor™ 488 (Thermo Fisher Scientific, A-11073, 1:1500), Goat anti-Rabbit IgG (H+L) Highly Cross-Adsorbed Secondary Antibody, Alexa Fluor™ 647 (Thermo Fisher Scientific, A-21245, 1:1500), Goat anti-Chicken IgY (H+L) Secondary Antibody, Alexa Fluor™ 488 (Thermo Fisher Scientific, A-11039, 1:1500), Goat anti-Chicken IgY (H+L) Secondary Antibody, Alexa Fluor™ 546 (Thermo Fisher Scientific, A-11040, 1:1500), Goat anti-Mouse IgG (H+L) Highly Cross-Adsorbed Secondary Antibody, Alexa Fluor™ 647 (Thermo Fisher Scientific, A-21236, 1:1500), and Goat anti-Rabbit IgG (H+L) Cross-Adsorbed Secondary Antibody, Alexa Fluor™ 546 (Thermo Fisher Scientific, A-11010, 1:1500), Goat anti-Mouse IgG (H+L) Highly Cross-Adsorbed Secondary Antibody, Alexa Fluor™ 488 (Thermo Fisher Scientific Catalog, A-11029, 1:1500).

These antibodies were used for STED microscopy imaging at the indicated dilution ratios: HA (mouse, Covance Cat, MMS101R; 1:1000), Homer1 (guinea pig, Synaptic Systems, 160004, 1:500), vGlut1 (rabbit, Yenzym 6089, 1:500), abberior STAR 460L, goat anti-mouse IgG (abberior, ST460L-1001, 1:500), abberior STAR RED, goat anti-guinea pig IgG (abberior, STRED-1006, 1:500), and abberior STAR ORANGE, goat anti-rabbit IgG (abberior, STORANGE-1002, 1:500).

### Quantitative RT-PCR

To determine the mRNA levels of BAIs in cultured cells, quantitative RT-PCR measurements were performed on total RNA (isolated with PrepEase RNA Spin Kit, Affymetrix) using TaqMan probes with TaqMan™ Fast Virus 1-Step Multiplex Master Mix for qPCR (Applied Biosystems) and a QuanStudio3 Real-Time PCR Systems (Thermo Fisher Scientific). The TaqMan primer/probe sets were designed and purchased from Integrated DNA Technologies (IDT). Mm Gapdh, Mm.PT.39a.1 from IDT was used as the internal control. The TaqMan primer/probe sets for this present study are:

BAI1 forward primer: TGCAAGTCGCTGGATCAAG

BAI1 probe: CCACAGGCAAACACCAGGAGC

BAI1 reverse primer: GGTTTCAGGAGACAGTGGAAG

BAI2 forward primer: AGCCGGAAATGCAGTGTG

BAI2 probe: ATCCGTGAGGGCACCTGCG

BAI2 reverse primer: ACACGTCTTGGAGCACAG

BAI3 forward primer 1: TGTCCACTGAATGCCACAG

BAI3 probe 1: TCCTGGGAACAGCCGAGCTTT

BAI3 reverse primer 1: CCTTCGCAAGGTGCTCTTTA

BAI3 forward primer 2: CCAAGAGACCACCCAAAGAAG

BAI3 probe 2: AAAAGTCAGCGACCTCGATCTGTCC

BAI3 reverse primer 2: TTCCACACCAGATTCACCAG

### Cell aggregation assay

As previously described^27^, 10 mL of FreeStyle 293-F cells were cultured in a flask and adjusted to a density of 1 × 10^6^ cells/mL prior to transfection (viability > 90%). In tube A, 10 µL of FreeStyle MAX Reagent (Thermo Fisher Scientific) was added to 167 µL of Opti-MEM (Thermo Fisher Scientific) and incubated at room temperature for 5 min. In tube B, 10 µg of total DNA (5 µg of pCMV5-GFP or pCMV5-mCherry plus 5 µg of the surface display plasmid) was added to 333 µL of Opti-MEM. Tubes A and B were mixed by pipetting, incubated at room temperature for 25 min, and then added to the 10 mL of cells.

After 48 h of transfection, cells were filtered using a 70 µm cell strainer to remove aggregated cells. GFP co-transfected cells and mCherry co-transfected cells were mixed at a 1:1 ratio in non-treated 6-well plates (SPL Life Sciences) and incubated with shaking in an incubator (125 rpm, 37 °C) for 1 h. Images were acquired using a Zeiss Cell Discoverer 7 Automated Microscope with a 5X objective and the built-in magnification changer set to X2. The aggregation index was calculated using the “Analyze Particles” feature in Fiji software. This index represents the percentage of the total surface area occupied by particles larger than 5000 pixels, relative to the total surface area of all particles in the sample. To quantify this index, we first measured the total surface area of all particles present. Then, we specifically measured the surface area of particles that exceeded the 5000 pixels threshold. Finally, the aggregation index was expressed as the percentage of the surface area of these larger particles compared to the overall particle surface area.

### Cell surface labeling assay

HEK293T cells were transiently transfected with 0.125 μg of pCMV5-mCherry and 0.5 μg of pCAG empty vector or vector encoding FLAG-tagged BAIs or BAI3 mutants (FuGENE® HD Transfection Reagent, Promega E2311). On the second day post-transfection, purified HA-tagged C1qls (containing only the C1q domain) were added to the cells at a final concentration of 100 nM in fresh DMEM media. The cells were incubated at room temperature for 1 h, followed by three washes with PBS (5 min each) and fixation in 4% paraformaldehyde (PFA) and 4% sucrose in PBS at 4 °C for 20 min. After three washes with PBS, cells were blocked in 5% BSA for 1 h at room temperature and then incubated overnight at 4 °C with the following primary antibodies: rabbit anti-FLAG antibody (1:500 in 5% BSA and PBS, Sigma Catalog # F7425) to label the BAI receptors and mouse anti-HA antibody (1:1000 in 5% BSA and PBS, Covance Catalog # MMS101R) for the C1qls. Following three additional washes with PBS, Goat anti-Mouse IgG (H+L) Highly Cross-Adsorbed Secondary Antibody, Alexa Fluor™ 488 (1:1500 in 5% BSA and PBS, Thermo Fisher Scientific Catalog # A-11029) and Goat anti-Rabbit IgG (H+L) Highly Cross-Adsorbed Secondary Antibody, Alexa Fluor™ 647 (1:1500 in 5% BSA and PBS, Thermo Fisher Scientific Catalog # A-21245) were applied for 1 hour at room temperature. After three more washes with PBS, coverslips were mounted on microscope slides using DAPI Fluoromount-G (SouthernBiotech, 0100-20). Fixed cells were imaged using a Nikon A1RSi confocal microscopy system 60x objective, and representative images along with related analyses were performed using Fiji software.

### Primary hippocampal culture of mixed neurons and glia

Postnatal day 0 (P0) mice of both genders were randomly used to make primary cultures. At DIV 0, Hippocampi or cortex were dissected from P0 mice, digested by papain (Worthington) for 20 min at 37 °C, filtered through a 70 μm cell strainer, and plated on Matrigel (Corning) coated coverslips in 24-well plates. Plating media contained 5% FBS (Atlanta), B27 (GIBCO), 0.4% glucose (Millipore-Sigma), 2 mM glutamine (GIBCO) in MEM (GIBCO). At DIV 1, culture medium was changed to growth medium containing 5% FBS (Atlanta), B27 (GIBCO), 2 mM glutamine (GIBCO) in Neurobasal A (GIBCO). At DIV 4, half medium was changed by growth medium containing 8 µM Ara-C (final concentration at 4 µM) (Millipore-Sigma), and neurons were analyzed at DIV 14.

### Lentivirus production and infection in hippocampal cultures

Lentiviral plasmids used in this study were: FSW-hSyn-NLS-EGFP-Cre, FSW-hSyn-NLS-EGFP-ΔCre, FSW-hSyn-NLS-tdTomato-Cre, FSW-hSyn-NLS-tdTomato-ΔCre, FSW-hSyn-Flag-mBAI3-HA, FSW-hSyn-Flag-mBAI3 T360V-HA, FSW-hSyn-Flag-mBAI3 R366A-HA, and FSW-hSyn-Flag-mBAI3 ΔN-HA. Lentiviruses were produced in Lenti-X 293T cells by co-transfection lentiviral plasmid (12 µg per T75 flask) with three helper plasmids (pRSV-REV, pMDLg/pRRE and vesicular stomatitis virus G protein (VSV-G); 4, 8, and 6 µg per T75 flask, respectively) with FuGENE HD Transfection Reagent (Promega E2311). Lentiviruses were harvested in the medium 48 h after transfection, concentrated by PEG-it Virus Precipitation Solution (System Biosciences), resuspended in 100 µL MEM (GIBCO), aliquoted, and frozen at -80 °C. 1-2 µL ΔCre and Cre lentiviruses were infected first at DIV 4 to ensure efficient gene deletion, and 3 µL BAI3 rescue lentiviruses were infected at DIV 6.

### Transduction efficiency of lentiviruses in hippocampal cultures

To quantify neuronal and glial lentiviral transduction efficiency, primary hippocampal cultures were prepared from BAI3 cKO mice and infected with either ΔCre or Cre at day in vitro (DIV) 4. At DIV 14, cells were washed once with pre-warmed PBS and then fixed in a solution containing 4% PFA and 4% sucrose in PBS for 20 minutes at 4 °C. Following fixation, cells were washed three times with PBS and blocked for 1 h at room temperature using a solution of 2.5% goat serum (Millipore-Sigma), 2.5% BSA, and 0.2% Triton X-100 in PBS for permeabilization. Primary antibodies against NeuN or NeuN and GFP were applied overnight at 4 °C. After another series of three PBS washes, cultures were incubated with secondary antibodies in the blocking buffer for 1 hour at room temperature. A final round of three PBS washes was performed before mounting coverslips on microscope slides using DAPI Fluoromount-G (SouthernBiotech, 0100-20). Imaging was conducted using a Nikon A1RSi confocal microscope. Quantifications were performed using NIS-Elements software (Nikon). Neurons were identified as NeuN+ and DAPI+, while glia were defined as NeuN- and DAPI+. Neuronal transduction efficiency was calculated as the fraction of neurons having EGFP signal, and glial transduction efficiency was calculated as the fraction of glia having EGFP signal.

For genotyping hippocampal cultures from BAI3 cKO mice, cells were washed once with pre-warmed PBS at DIV 14. Genomic DNA was extracted using QuickExtract DNA extraction solution (Lucigen). The extracted DNA was PCR amplified with CloneAmp HiFi PCR Premix (Takara) and analyzed by gel electrophoresis. Controls were genomic DNAs extracted from homozygous BAI3 cKO mice. Band intensities were quantified using Fiji software. The primers were designed and purchased from Integrated DNA Technologies (IDT) and are listed below:

BAI3 primer 1: AAAGCAAGGAGGCGTATCCAG

BAI3 primer 2: TCTGTGGTGCAAGGAACCAT

GAPDH primer 1: ACCACAGTCCATGCCATCAC

GAPDH primer 2: TCCACCACCCTGTTGCTGTA

### Measurement of surface expression levels of BAI3 WT and mutants

At DIV 14, primary hippocampal cultures underwent a single wash with pre-warmed PBS before being fixed in a solution containing 4% PFA and 4% sucrose in PBS for 20 min at 4 °C. Following three washes with PBS, the cultures were blocked using a buffer composed of 2.5% goat serum (Millipore-Sigma) and 2.5% BSA in PBS, without permeabilization. The Flag antibody was then applied in the same blocking buffer and incubated overnight at 4 °C. After three additional washes with PBS, the secondary antibody was introduced in the same blocking buffer for 1 hour at room temperature. The cells were then washed three times with PBS and re-fixed in the 4% PFA, 4% sucrose and PBS solution for another 20 min at 4 °C. After three more PBS washes, a new blocking buffer containing 2.5% goat serum (Millipore-Sigma), 2.5% BSA, and 0.2% Triton X-100 (for permeabilization) was applied for 1 h at room temperature. The HA and MAP2 antibodies were subsequently added in this new blocking buffer and incubated overnight at 4 °C. Following another series of three PBS washes, the cultures were incubated with secondary antibodies in the same new blocking buffer for 1 hour at room temperature. After a final round of three PBS washes, coverslips were mounted on microscope slides using DAPI Fluoromount-G (SouthernBiotech, 0100-20). Imaging was performed with a Nikon A1RSi confocal microscope equipped with a 60x objective lens. Image analysis was conducted using Fiji software, where the intensity ratio of Flag/MAP2 was used as an indicator of expression levels.

### Morphology analysis

Hippocampal cultures were sparsely transfected with tdTomato by calcium phosphate transfection (CalPhos™ Mammalian Transfection Kit, Takara 631312) at DIV 9 (for axon tracing, 0.5 µg DNA per well in 24-well plates; for dendrite arborization and soma area, 1.0 µg DNA per well in 24-well plates). At DIV 14, cells were washed once with PBS and fixed in 4% paraformaldehyde (PFA), 4% sucrose and PBS for 20 min at 4 °C. After washed with PBS three times again, coverslips were mounted on microscope slides. Images were taken by a Nikon A1RSi confocal microscopy. Axon tracing was performed using a 10X objective lens, while dendrite morphology and soma area were examined with a 60X objective lens. The soma area was measured using Fiji software. For analyzing axonal and dendritic arborizations, the Simple Neurite Tracer (SNT) plugin in Fiji software was utilized^53,54^

### Immunocytochemistry and quantifications for synapse density

At DIV 14, primary hippocampal cultures were washed once with pre-warmed PBS and fixed in 4% PFA, 4% sucrose and PBS for 20 min at 4 °C. After three times wash with PBS, cells were blocked in PBS containing 2.5% goat serum (Millipore-Sigma), 2.5% BSA and 0.2% Triton X-100 for 1 hour at room temperature. Primary antibodies were applied in the same blocking buffer overnight at 4 °C. Cells were washed in PBS three times and incubated with fluorescence-labeled secondary antibodies in blocking buffer for 1 h at room temperature. After three times wash with PBS, coverslips were mounted on microscope slides by DAPI Fluoromount-G (SouthernBiotech, 0100-20). All images were taken by a Nikon A1RSi confocal microscopy system 60x objective.

Quantifications of the synaptic puncta were performed by general analysis in NIS-Elements software (Nikon) as previously described^55^. For each batch of samples, the average background intensities for each channel were calculated and subsequently subtracted from the corresponding images. A uniform binary mask was applied consistently across all images within the same batch. Three regions of interest (ROIs) were identified for each neuron along three distinct primary dendrites. The soma was positioned at the center of the image, with each ROI encompassing the entire length of the primary dendrite, extending approximately 100 μm from the edge of the soma to the edge of the image. Synapse density was determined by counting the number of colocalized vGlut1 and Homer1 puncta or vGAT and gephyrin puncta and dividing this by the length of the dendritic segment. The puncta size was calculated by dividing the total binary area of the puncta by the number of objects identified within the ROI. The overall synapse density or puncta size for each neuron was calculated by averaging the densities obtained from the three ROIs. The representative images were processed by Fiji software.

### Immunocytochemistry and analyses for STED microscopy imaging

At DIV 14, primary hippocampal cultures were washed once with pre-warmed PBS and fixed in 4% PFA, 4% sucrose and PBS for 20 min at 4 °C. After three times wash with PBS, cells were blocked in PBS containing 5% goat serum (Millipore-Sigma) and 0.2% Triton X-100 for 1 h at room temperature. Primary antibodies were applied in the same blocking buffer overnight at 4 °C. Cells were washed in PBS three times and incubated with fluorescence-labeled secondary antibodies in blocking buffer for 1 h at room temperature. After three times wash with PBS, coverslips were mounted on microscope slides by ProLong™ Gold Antifade Mountant (Thermo Fisher Scientific, P36930). All images were taken by a Nikon Ti2-E microscope stand equipped with a STEDYCON confocal and STED module from Abberior Instruments, Inc. Please note the STED imaging captures only a single layer snapshot without a “z” dimension.

Images were analyzed by the Huygens Software and Nikon NIS-Elements as previously described^49^. A consistent binary mask was applied across all images in each batch. For each neuron, three polygonal regions of interest (ROIs) were defined. A “synapse” was identified when vGlut1 had at least one pixel overlapping with the Homer1 mask, or vice versa. The “synaptic BAI3” was calculated as the proportion of BAI3 puncta (number, not area) that had at least one pixel overlapping with a “synapse”. “Synapse having BAI3” was assessed by calculating the number of synapses that had at least one pixel overlapping with the BAI3 mask. “A having B” is defined as the fraction of A puncta number having at least one pixel overlapping with B. The puncta size was calculated by dividing the total binary area of the puncta by the number of objects identified within the ROI. The average value from the three ROIs for each neuron was averaged in the analysis.

### Electrophysiology

Electrophysiological recordings were performed from primary hippocampal culture neurons plated on coverslip, which were placed in a recording chamber mounted on a fixed stage inverted phase-contrast microscope (Olympus)^56,57^. Patch electrodes (3-5 MΩ) were pulled from borosilicate glass capillary tubes (Warner Instruments) using a PC-10 pipette puller (Narishige). Whole-cell capacitance and series resistances were recorded and compensated to >80%, and in addition, series resistances were less than two times the tip resistance. The Tyrode’s bath solution contained (in mM): 129 NaCl, 5 KCl, 2 CaCl_2_, 1 MgCl_2_, 0.01 glycine, 30 D-glucose and 25 HEPES, pH 7.2–7. 4.

All recording under voltage-clamp model with a pipette solution containing (in mM): 135 CsCl, 10 HEPES, 10 EGTA, 2 Mg-ATP, 2 Na_2_GTP, and 5 QX-314, pH 7.35 (adjusted with CsOH). Miniature excitatory postsynaptic currents (mEPSCs) were pharmacologically isolated with picrotoxin (50 µM) and recorded at a -70mV holding potential, mEPSCs were monitored in the presence of tetrodotoxin (1 µM). Presynaptic Action-potential for evoked synaptic responses were triggered by 0.5 ms current (40–90 μA) injections through a local extracellular electrode (FHC concentric bipolar electrode, Catalogue number CBAEC75) placed ~100 μm from the soma of neurons recorded. The frequency, duration, and magnitude of the extracellular stimulus were controlled with a Model 2100 Isolated, Pulse Stimulator (A-M Systems, Inc.) synchronized with the Clampex 10.2 data acquisition software (Molecular Devices). To determine paired-pulse ratios (PPRs) of evoked synaptic responses, two sequential APs were elicited with a given time interval. Each paired stimulation intervals (20, 50, 100, 500, and 1000 ms) were repeated 3 times with an intertrial-interval of 30 s. All Data were filter during acquisition with a low pass filter set at 2 kHz using pClamp 10 (Molecular Devices), Data analysis was performed offline with Clampfit 10.2 (Molecular Devices).

### Expression analyses of select genes across hippocampal cell types

For Fig. 8, the expression matrix of the SMART-Seq v4 dataset and corresponding cell type annotations were downloaded from the NeMO Archive for the BRAIN Initiative Cell Census Network (https://assets.nemoarchive.org/dat-jb2f34y). Transcriptomic data analysis and visualization were performed using R with the RStudio IDE (https://www.rstudio.com/). Hippocampus-specific data were subsequently extracted for evaluating RPKM-normalized expression profiles of candidate genes.

### Protein expression

The BAI extracellular domains and C1ql1-4 C1q domain constructs were cloned into the pEG BacMam vectors with 6X His tag. Proteins were expressed in Expi293F^TM^ cells following the Thermo Fisher Scientific protocol. Briefly, Expi293 cells transfected at a density of 3 x 10^6^ cells/mL using ExpiFectamine^TM^ 293 transfection kit (Thermo Fisher Scientific, A14524) following enhancer addition 16 hrs post transfection. Cells were further incubated for either 48 or 72 h before harvesting. Protein purification was performed using Ni^2+^-NTA affinity column chromatography and subsequent size exclusion chromatography (SEC) using 1X HBS buffer (10 mM HEPES, pH 7.2, 150 mM NaCl). Notably, for C1ql1-4 purification, 2 mM CaCl_2_ was included in size exclusion chromatography buffers. Protein size exclusion chromatography data were exported from UNICORN (Cytiva) and plotted using GraphPad Prism 9 software.

### Quantification And Statistical Analysis

Statistical analyses were performed using GraphPad Prism 9 and 10 software. Comparisons between two groups were performed using unpaired two-tailed Student’s t-tests (n.s., not significant, * *p*<0.05, ** *p*<0.01, *** *p*<0.001, **** *p*<0.0001). Comparisons for more than two groups were calculated using one-way ANOVA followed by Dunnett’s multiple comparison tests (n.s., not significant, * p<0.05, ** p<0.01, *** p<0.001, **** p<0.0001) or two-way ANOVA. The genotype distribution of BAI1 KO mice was analyzed by chi-square test. Two-tailed Kolmogorov–Smirnov test at a 95% confidence level was performed for cumulative distributions in electrophysiological recordings.

### Lead Contact

Further information and requests for resources and reagents should be directed to and will be fulfilled by the Lead Contact Thomas C. Südhof (tcs1@stanford.edu).

### Reporting summary

Further information on research design is available in the Nature Portfolio Reporting Summary linked to this article.

## Supplementary information

Supplementary Information

Reporting Summary

Transparent Peer Review file

## Source data

Source Data

## Data Availability

Source data are provided with this paper.

## Code availability

This paper does not report original code.
