# Supplementary material for: BAI adhesion-GPCRs perform distinct functions in neural development differentially controlled by RTN4R and C1ql ligands
Source: Nat Commun. 2025 Dec 23;17:6140. doi: 10.1038/s41467-025-67453-6 (PMC13365561; doi:10.1038/s41467-025-67453-6)
Supplement: Supplementary file 1 — Supplementary Information [file 41467_2025_67453_MOESM1_ESM.pdf]

Supplementary Information

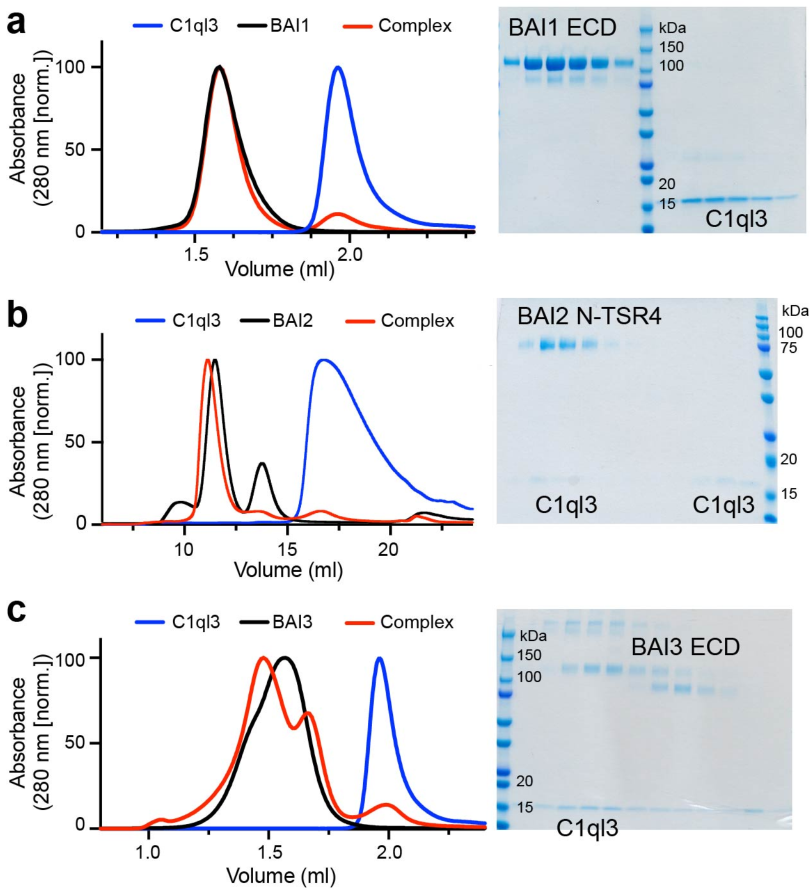

Supplementary Fig. 1: BAI3 and BAI2 form stable complexes with C1ql3 during size-exclusion chromatography, whereas BAI1 does not bind to C1ql3.

(**a**) The entire BAI1 extracellular domains (ECD; residues 34-948) do not form a complex with C1ql3 (residues 122-255) as monitored by size-exclusion chromatography (left, elution profile of purified proteins; right, Coomassie-stained SDS-PAGE gel of selected fractions).

(**b**) The N-terminal extracellular domains of BAI2 (N-TSR4, residues 21-519 including the N-terminal domain and thrombospondin repeats) forms a complex with C1ql3 (residues 122-255) as monitored by size-exclusion chromatography (left, elution profile of purified proteins; right, Coomassie-stained SDS-PAGE gel of selected fractions). The experiment used the N-terminal domains instead of the complete extracellular sequences of BAI2 because the latter could not be expressed in high yield.

(**c**) The entire BAI3 extracellular domains (ECD; residues 26-880) form a complex with C1ql3 (residues 122-255) as monitored by size-exclusion chromatography (left, elution profile of purified proteins; right, Coomassie-stained SDS-PAGE gel of selected fractions).

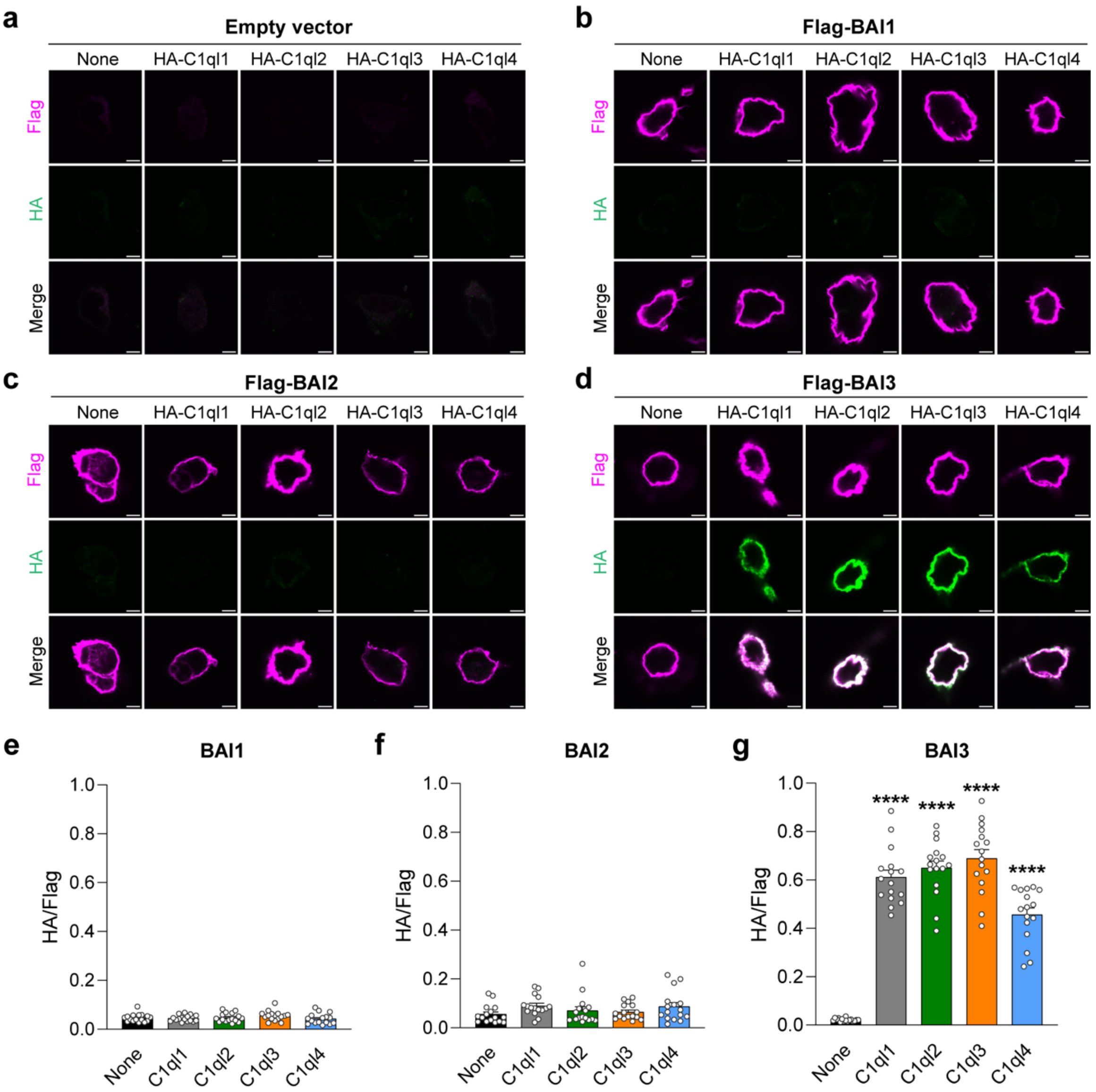

Supplementary Fig. 2: BAI3 interacts strongly with all C1ql isoforms (C1ql1-4) in cell surface labeling assays, whereas BAI1 and BAI2 exhibit little binding.

(**a-d**) Cell surface staining of the HA-C1q domain of C1ql1-4 in HEK293T cells expressing either the empty vector (a), FLAG-BAI1 (b), FLAG-BAI2 (c), or FLAG-BAI3 (d). The top, middle, and bottom rows display FLAG staining (magenta), HA staining (green), and the merged image, respectively. Scale bars are 5 µm.

(**e-g**) Quantification of the HA to FLAG intensity ratio for experiments in (**b-d**). Data are means ± SEM (n=16/2 cells/experiments). Statistical analyses were performed with one-way ANOVA followed by Dunnett's multiple comparison tests (**** $p < 0.0001$). In (**e**), p = 0.3916 for ANOVA; for multiple comparisons, p = 0.9956 (None vs. C1ql1), 0.9996 (None vs. C1ql2), 0.6044 (None vs. C1ql3) and 0.9562 (None vs. C1ql4). In (**f**), p = 0.2140 for ANOVA; for multiple comparisons, p = 0.1462 (None vs. C1ql1), 0.7851 (None vs. C1ql2), 0.9508 (None vs. C1ql3) and 0.1988 (None vs. C1ql4). In (**g**), p< 0.0001 for ANOVA; for multiple comparisons, p < 0.0001 for None vs. C1ql1, C1ql2, C1ql3 or C1ql4. Note that our recently published cryo-EM structure

of the complex of the BAI3 NTD with the trimeric C1q-like domains of C1ql3 reveals a 3:3 stoichiometry with the BAI3 NTD inserting into the groove formed by two C1q-like domains[42]. Analysis of the interface residues and binding measurements confirms that the BAI1 NTD cannot bind to C1qls whereas the BAI2 NTD likely binds weakly.

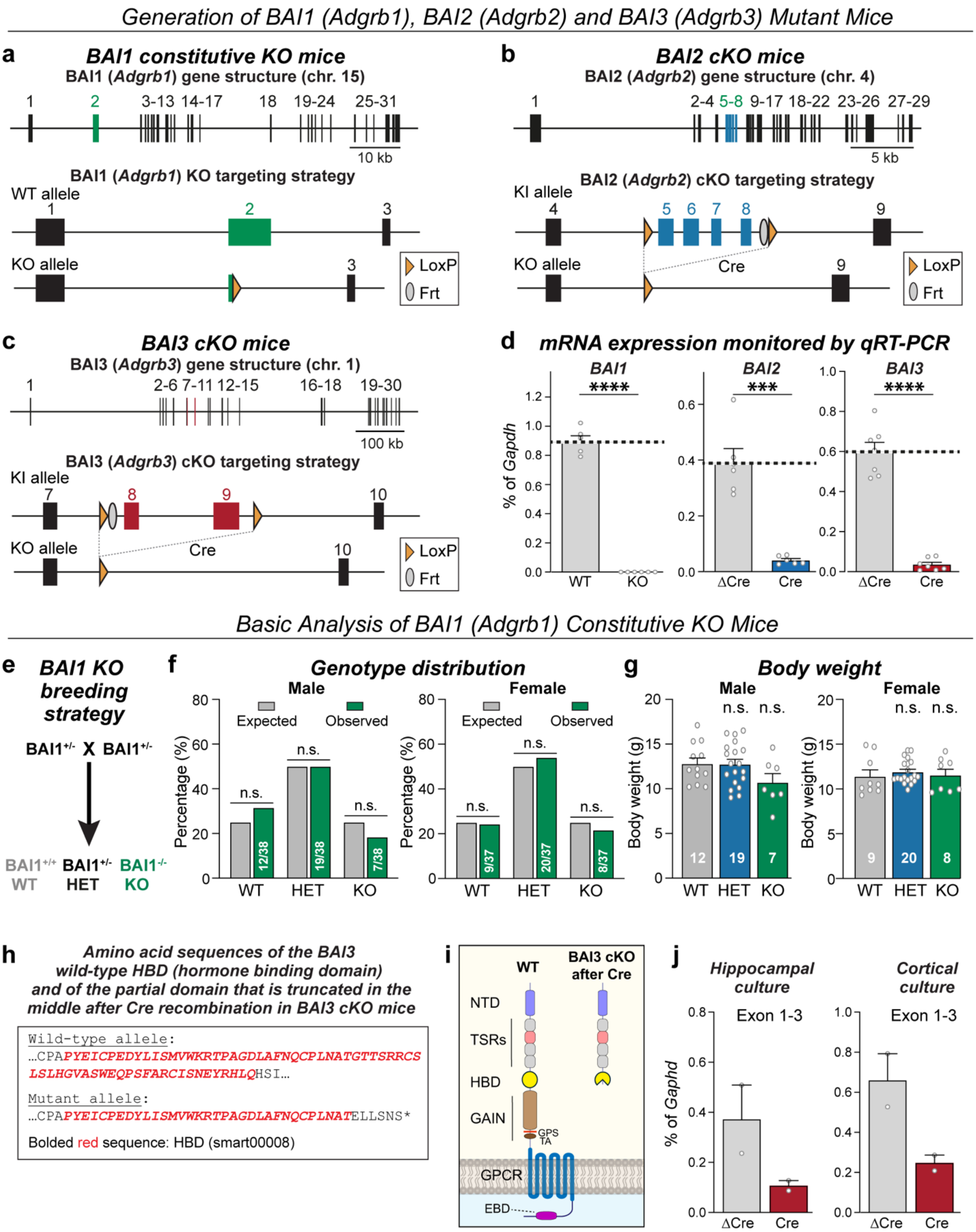

Supplementary Fig. 3: Generation of BAI1-3 mutant mice (a-d), genotype distribution and body weight of BAI1 knockout (KO) mice (e-g), and analysis of BAI3 KO effects on the encoded remnant BAI3 protein (h-j).

(**a-c**) Diagrams of the gene structures of BAI1 (*Adgrb1*, **a**), BAI2 (*Adgrb2*, **b**), and BAI3 (*Adgrb3*, **c**), and of the constitutive BAI1 and conditional BAI2 and BAI3 knockout (cKO)

strategies to delete the expression of these BAI's in mice. Note that the BAI2 and BAI3 cKO mice were reported previously[15].

(**d**) Validation of the BAI deletion by qRT-PCR in hippocampal cultures from BAI1 KO, BAI2 cKO and BAI3 cKO mice at DIV 14. For BAI1, cultures from wild-type (WT) and littermate homozygous KO mice were analyzed, and qRT-PCR primers and probe are targeted at exon 1-2. For BAI2 and BAI3 cKO, cultures were infected with lentiviruses expressing Cre recombinase (Cre) or a non-functional mutant Cre recombinase (ΔCre, control). qRT-PCR primers and probe are targeted at exon 5-6 and exon 7-9 for *Bai2* and *Bai3*, respectively. *Gapdh* was used as an internal control (n = 6, 6, and 7 independent batches of cultures for *Bai1*, *Bai2*, and *Bai3*, respectively).

(**e**) BAI1 KO mice breeding strategy: heterozygous parents were crossed to produce wild-type (WT) and littermate homozygous knockout (KO) offspring for experiments.

(**f**) Genotype distribution of BAI1 KO offspring at P21. Grey bars represent the expected percentage for each genotype, calculated through Mendelian inheritance from heterozygous parents. A chi-square test revealed no significant disparity between the observed and expected distribution of BAI1 KO offspring. Numbers within bars are the count of mice per genotype and the total number of mice.

(**g**) Quantification of body weight of BAI1 KO offspring at P21. Number of mice are indicated in bars.

(**h**) Amino acid sequence of the hormone-binding domain (HormR domain [smart00008]; also called 'hormone receptor domain') in wild-type mouse BAI3 and in the BAI3 KO after deletion of exons 8 and 9. Note that the deletion causes a premature stop codon and thereby disrupts the hormone-binding domain in the middle, thus preventing proper folding of this domain.

(**i**) Diagram of the wild-type BAI3 protein and the encoded N-terminal protein fragment after Cre-mediated deletion of exons 8 and 9. The truncated fragment is likely unstable given the inability of the HBD to fold after Cre-recombination.

(**j**) Further validation of the BAI3 deletion by qRT-PCR targeting exons outside of the loxP-flanked regions in hippocampal and cortical cultures from BAI3 cKO mice at DIV 14. Cultures were infected with lentiviruses expressing either Cre recombinase (Cre) or a non-functional mutant Cre recombinase (ΔCre, control). qRT-PCR primers and probes targeted exons 1-3 of the BAI3 gene. GAPDH was used as an internal control. Data represent results from 2 independent batches of hippocampal and cortical cultures. Please note that in Figure S3 of the previously published paper[21], Southern blot analysis of genomic DNA from ES clones demonstrated that the knockout strategy effectively deletes the targeted exons. In the same paper[21], immunogold electron microscopy (EM) of endogenous BAI3 showed that the BAI3 protein signal is absent. The additional qRT-PCR data shown here, together with the demonstration in panel d that the Cre-mediated deletion is nearly 100% effective, and the previously published data[21] suggest that the mRNA generated after deletion of exons 8 and 9 is unstable and subject to missense-mediated decay. This instability arises because the deletion

disrupts the hormone-binding domain in the middle of the BAI3 protein, impairing its folding. Together, these findings confirm that the BAI3 KO is truly a fully KO of the protein.

Data in (**d**), (**g**) and (**j**) are means ± SEM. Statistical analyses were performed with Student's t-test (**d**) and one-way ANOVA followed by Dunnett's multiple comparison tests (**g**) (n.s., not significant, *** p<0.001, **** p<0.0001). In **(d)**, p <0.0001 for BAI1 and BAI3, p = 0.0006 for BAI2. In (**g**), male group: p = 0.1383 for ANOVA; for multiple comparisons, p = 0.9978 (WT vs. HET) and 0.1360 (WT vs. KO). In (**g**), female group: p = 0.7188 for ANOVA; for multiple comparisons, p = 0.6679 (WT vs. HET) and 0.9824 (WT vs. KO).

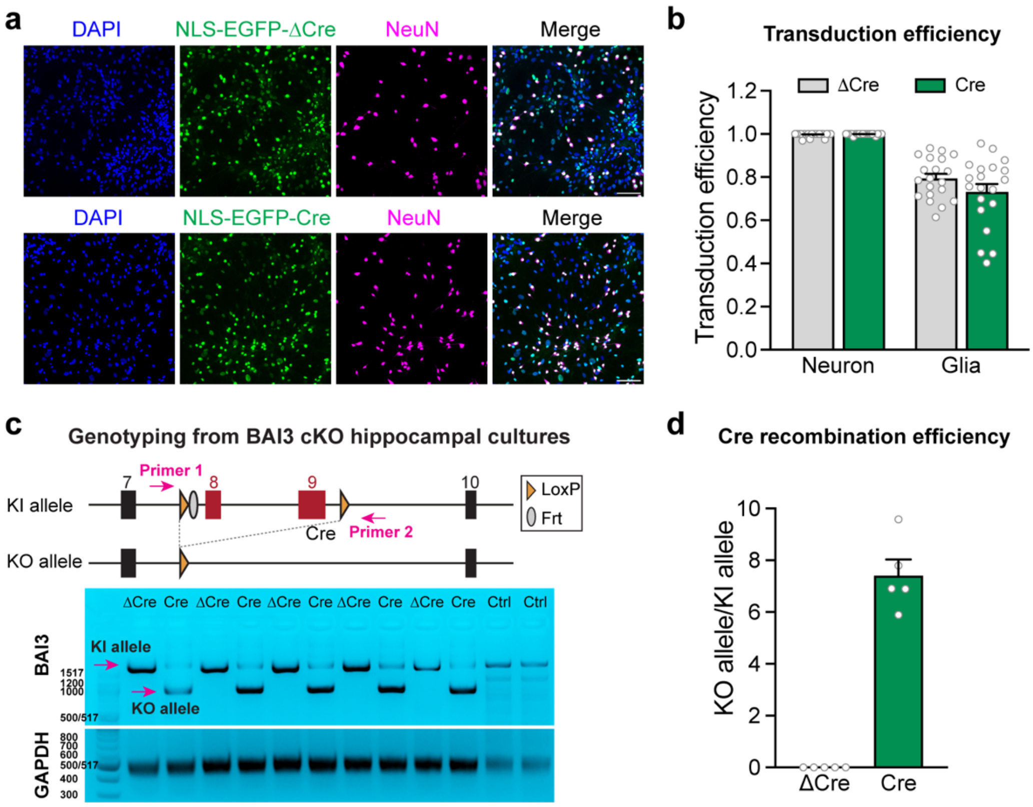

Supplementary Fig. 4: Transduction efficiency of ΔCre and Cre lentiviruses in cultured hippocampal neurons.

(**a**) Representative immunocytochemistry images of mixed neuron-glia hippocampal cultures from BAI3 conditional knockout (cKO) mice infected with lentiviruses expressing either NLS-EGFP-ΔCre or Cre at DIV4, analyzed at DIV14. DAPI (blue) labels nuclei, ΔCre or Cre (green) indicates viral expression, and NeuN (magenta) labels neurons. Scale bars represent 100 µm. (**b**) Quantification of transduction efficiency of ΔCre or Cre in neurons (NeuN+ and DAPI+) and glia (NeuN- and DAPI+). Data are means ± SEM. N = 20/3 (cells/experiments) for each group. (**c**) Upper: schematic representation of BAI3 cKO mice and the primers (magenta) used for genotyping BAI3 cKO hippocampal cultures. Below: gel electrophoresis image displaying the knockin (KI) and knockout (KO) alleles in cultures infected with ΔCre or Cre lentiviruses. Control (Ctrl) represents genomic DNA from homozygous BAI3 cKO mice. (**d**) Quantification of the KO to KI allele intensity ratio from (c). Data are shown as means ± SEM. N = 5 independent batches for each group.

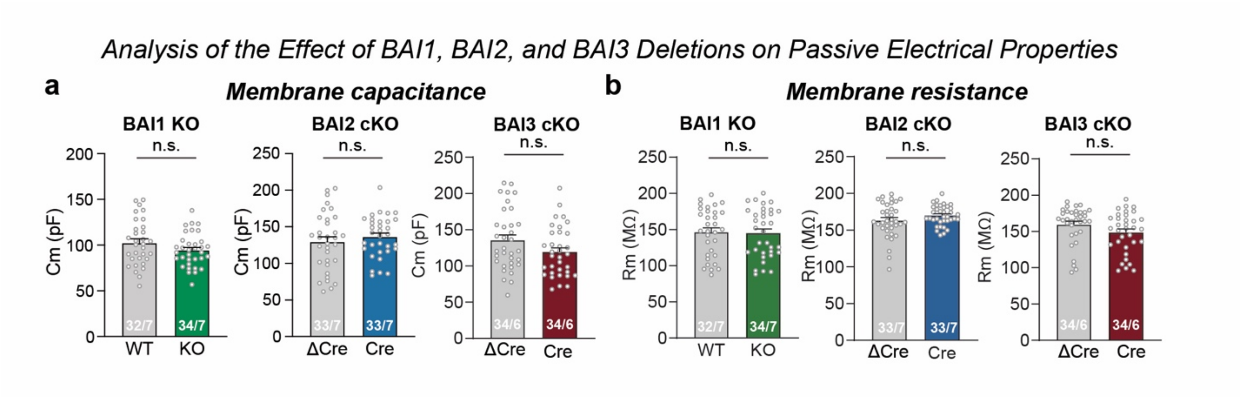

Supplementary Fig. 5: Effect of BAI1-3 deletions on passive electrical properties in cultured hippocampal neurons.

(**a** & **b**) Summary graphs of membrane capacitance (**a**) and membrane resistance (**b**) from the indicated cultured hippocampal neurons.

Data are means ± SEM. The numbers of cells/experiments are indicated in the bars. Unpaired two-tailed Student's t-tests were used (n.s., not significant). In (**a**), BAI1 KO WT vs KO, $p = 0.1440$; BAI2 cKO ΔCre vs. Cre, $p = 0.4092$; BAI3 cKO ΔCre vs. Cre, $p = 0.0819$. In (**b**), BAI1 KO WT vs KO, $p = 0.8786$; BAI2 cKO ΔCre vs. Cre, $p = 0.1426$; BAI3 cKO ΔCre vs. Cre, $p = 0.0921$.

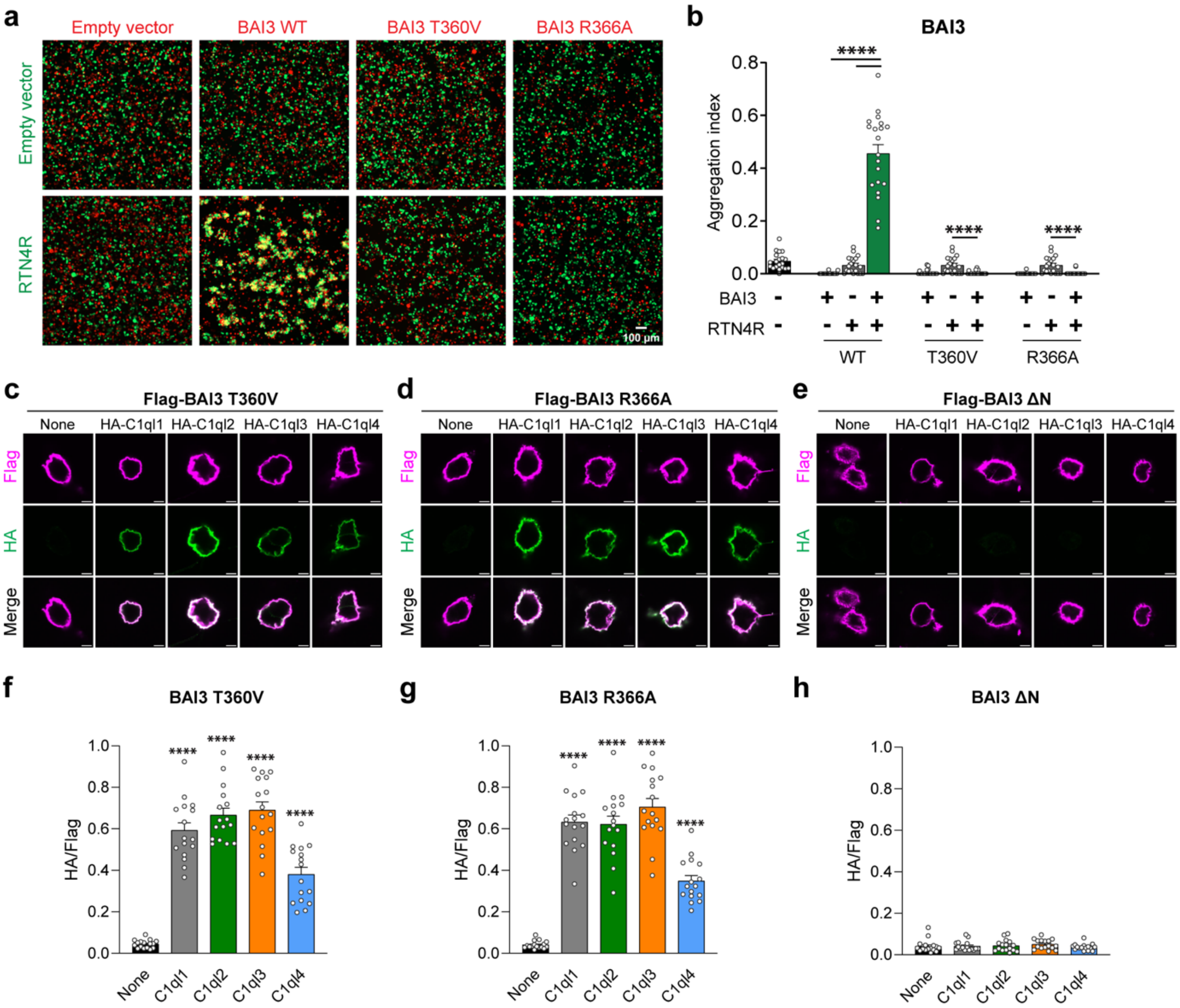

Supplementary Fig. 6: BAI3 T360V and R366A do not bind RTN4R, and BAI3 ΔN does not bind to C1qls.

(**a**) Cell aggregation assays were conducted using wild-type (WT) or mutant BAI3 in FreeStyle 293-F cells co-expressing mCherry or GFP along with the indicated proteins or empty vector (EV). (**b**) Quantification for experiments in (a). N = 20/2 (fields/experiments) for each group. (**c**-**e**) Cell surface staining of the HA-C1q domain of C1ql1-4 in HEK293T cells expressing FLAG-BAI3 T360V (c), FLAG-BAI3 R366A (d), or FLAG-BAI3 ΔN (e). The images display FLAG staining (magenta) in the top row, HA staining (green) in the middle row, and the merged images in the bottom row. Scale bars represent 5 μm. (**f**-**h**) Quantification of the HA to FLAG intensity ratio for the experiments shown in (c-e). N = 16/2 (cells/experiments) for each group. Please note experiments in (c-h) were performed in parallel with those in Supplementary Fig. 2. Data are means ± SEM. Statistical analyses were performed using one-way ANOVA followed by Dunnett's multiple comparison tests (**** $p < 0.0001$). In (**b**), WT group: $p < 0.0001$ for ANOVA; for multiple comparisons, $p < 0.0001$ for WT + RTN4R vs. WT +EV or EV + RTN4R. In (**b**), T360V group: $p < 0.0001$ for ANOVA; for multiple comparisons, $p = 0.9159$ for T360V + RTN4R vs. T360V +EV, and < 0.0001 for T360V + RTN4R vs. EV + RTN4R. In (**b**), R366A group: $p < 0.0001$ for ANOVA; for multiple

comparisons, p = 0.9236 for R366A + RTN4R vs. R366A +EV, and < 0.0001 for R366A + RTN4R vs. EV + RTN4R. In (**f**) and (**g**), p< 0.0001 for ANOVA; for multiple comparisons, p < 0.0001 for None vs. C1ql1, C1ql2, C1ql3 or C1ql4. In (**h**), p = 0.6473 for ANOVA; for multiple comparisons, p = 0.9997 (None vs. C1ql1), 0.9898 (None vs. C1ql2), 0.6311 (None vs. C1ql3) and 0.9850 (None vs. C1ql4).

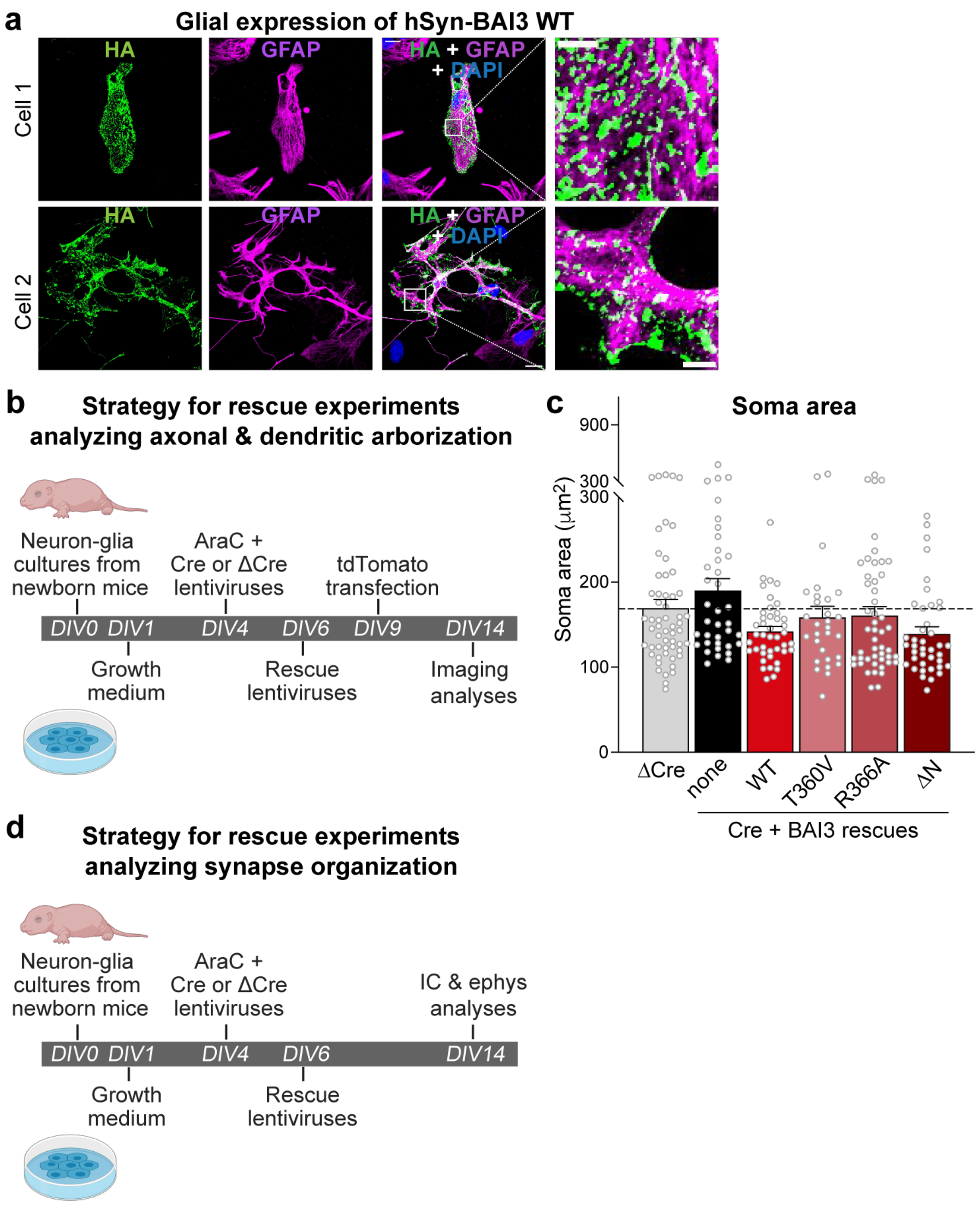

Supplementary Fig. 7: Expression of wild-type (WT) BAI3 in glia of hippocampal cultures, rescue experiment strategy, and lack of an effect of the BAI3 deletion and the BAI3 rescue on soma size.

(**a**) Representative images of hippocampal astrocytes in mixed neuron-glia cultures stained for HA-tagged BAI3 (green), GFAP (magenta) and DAPI (blue) to illustrate that the synapsin-1 promoter in lentiviruses also drives gene expression in astrocytes (scale bar is 20 μm on the third image, and 5 μm on the fourth image).

(**b**) Experimental timeline for rescue experiments analyzing axonal and dendritic arborizations in BAI3-deficient hippocampal neuron-glia cultures, related to **Fig 4**. Created in BioRender. Miao, Y. (2025) https://BioRender.com/6ryzwic.

(**c**) Quantification of the soma area for experiments in **Fig. 4d, 4e**. Data are means ± SEM (n's [cells/experiments] for each column [left to right] = 56/7, 37/7, 42/7, 29/6, 49/7, and 40/6). One-way ANOVA followed by Dunnett's multiple comparison tests were used and no statistical significance was detected. For ANOVA, p = 0.0080; for multiple comparisons, p = 0.4594 (ΔCre vs. Cre), 0.1668 (ΔCre vs. Cre + BAI3 WT), 0.9341 (ΔCre vs. Cre + BAI3 T360V), 0.9518 (ΔCre vs. Cre + BAI3 R366A) and 0.1155 (ΔCre vs. Cre + BAI3 ΔN).

(**d**) Experimental timeline for rescue experiments analyzing synapse numbers and electrophysiological responses in BAI3-deficient hippocampal neuron-glia cultures, related to **Fig 5, 6**. Created in BioRender. Miao, Y. (2025) https://BioRender.com/6ryzwic.

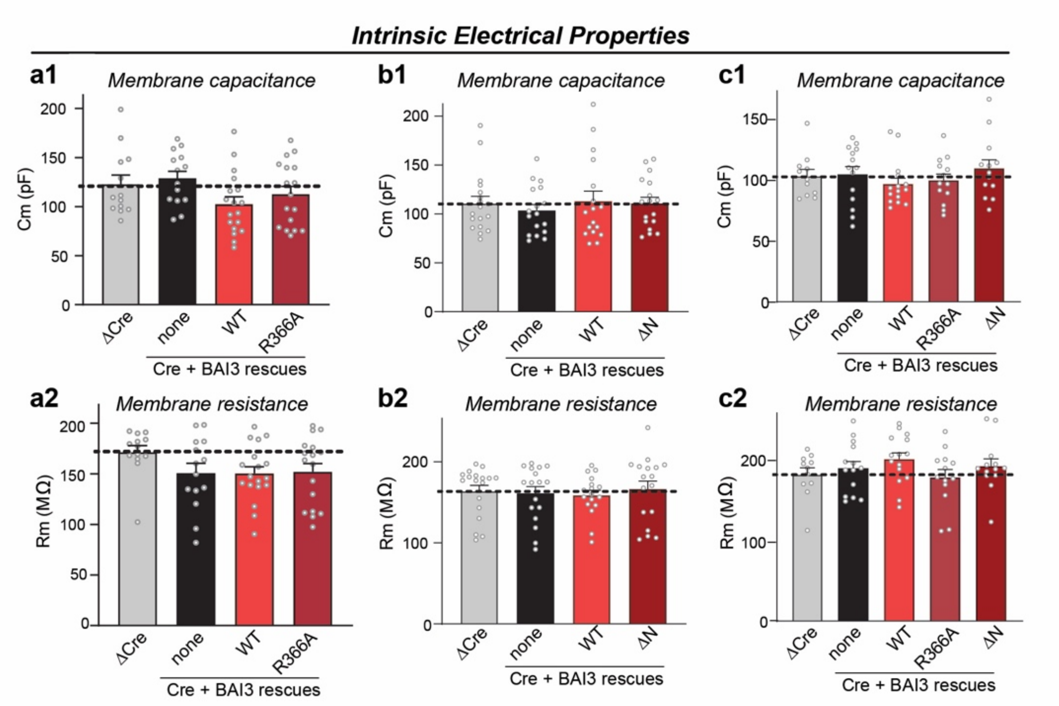

Supplementary Fig. 8: The BAI3 deletion and rescue manipulations have no effect on the intrinsic electrical properties of cultured neurons.

(**a1**–**c1** & **a2**–**c2**) Quantifications of membrane capacitance (**a1**-**c1**) and membrane resistance (**a2**-**c2**) during the electrophysiological recordings described in **Fig. 6a** (**a1** & **a2**; n's [cells/experiments] = 13/4, 14/4, 18/4, and 17/4 for each column from left to right), **Fig. 6b** (**b1** & **b2**; n's [cells/experiments] = 18/5, 17/5, 17/5, and 17/5 for each column, from left to right) and **Fig. 6c**-**6e** (**c1** & **c2**; n's [cells/experiments] = 11/4, 14/4, 15/4, 13/4, and 13/4 for each column, from left to right). For all graphs, data are means ± SEM. Statistical significance was examined by one-way ANOVA followed by Dunnett's multiple comparison tests, and no significant change was detected. In (**a1**), For ANOVA, p = 0.1061; for multiple comparisons, p = 0.9598 (ΔCre vs. Cre), 0.2979 (ΔCre vs. Cre + BAI3 WT), and 0.8225 (ΔCre vs. Cre + BAI3 R366A). In (**a2**), For ANOVA, p = 0.1796; for multiple comparisons, p = 0.3093 (ΔCre vs. Cre), 0.2463 (ΔCre vs. Cre + BAI3 WT), and 0.3270 (ΔCre vs. Cre + BAI3 R366A). In (**b1**), For ANOVA, p = 0.8375; for multiple comparisons, p = 0.9087 (ΔCre vs. Cre), 0.9967 (ΔCre vs. Cre + BAI3 WT), and >0.9999 (ΔCre vs. Cre + BAI3 ΔN). In (**b2**), For ANOVA, p = 0.3743; for multiple comparisons, p = 0.9959 (ΔCre vs. Cre), 0.9721 (ΔCre vs. Cre + BAI3 WT), and 0.9950 (ΔCre vs. Cre + BAI3 ΔN). In (**c1**), For ANOVA, p = 0.5836; for multiple comparisons, p = 0.9993 (ΔCre vs. Cre), 0.8521 (ΔCre vs. Cre + BAI3 WT), 0.9822 (ΔCre vs. Cre + BAI3 R366A) and 0.8667 (ΔCre vs. Cre + BAI3 ΔN). In (**c2**), For ANOVA, p = 0.0.3837; for multiple comparisons, p = 0.9245 (ΔCre vs. Cre), 0.3550 (ΔCre vs. Cre + BAI3 WT), 0.9964 (ΔCre vs. Cre + BAI3 R366A) and 0.8217 (ΔCre vs. Cre + BAI3 ΔN).

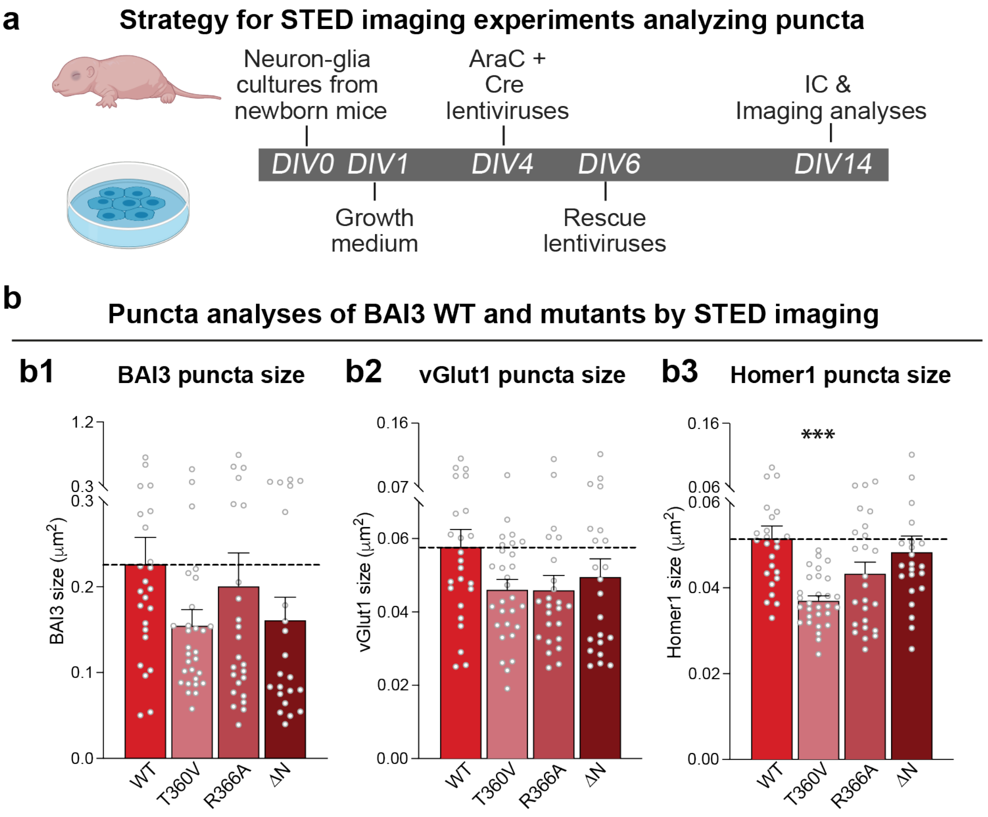

Supplementary Fig. 9: Puncta analyses of wild-type and mutant BAI3 proteins visualized by STED imaging.

(**a**) Experimental timeline for STED imaging experiments analyzing synaptic puncta and BAI3 puncta in BAI3-deficient hippocampal neuron-glia cultures, related to **Fig 7**. Created in BioRender. Miao, Y. (2025) https://BioRender.com/6ryzwic.

(**b**) Detailed quantification of BAI3 puncta size (**b1**), vGlut1 puncta size (**b2**), Homer1 puncta size (**b3**) for wild-type and mutant HA-tagged BAI3 proteins used in STED imaging experiments in **Fig. 7**. Data are means ± SEM (n's [cells/experiments] for each column [left to right] = 24/3, 29/3, 24/3, and 22/3). One-way ANOVA followed by Dunnett's multiple comparison tests were used (*** $p<0.001$). In (**b1**), p = 0.2461 for ANOVA; for multiple comparisons, p = 0.1742 (WT vs. T360V), 0.8654 (WT vs. R366A) and 0.2867 (WT vs. ΔN). In (**b2**), p = 0.1346 for ANOVA; for multiple comparisons, p = 0.0936 (WT vs. T360V), 0.1082 (WT vs. R366A) and 0.3686 (WT vs. ΔN). In (**b3**), p = 0.0005 for ANOVA; for multiple comparisons, p = 0.0003 (WT vs. T360V), 0.0716 (WT vs. R366A) and 0.7300 (WT vs. ΔN).
