# Supplementary material for: BAI adhesion-GPCRs perform distinct functions in neural development differentially controlled by RTN4R and C1ql ligands
Source: Nat Commun. 2025 Dec 23;17:6140. doi: 10.1038/s41467-025-67453-6 (PMC13365561; doi:10.1038/s41467-025-67453-6)
Supplement: Supplementary file 2 — Reporting Summary [file 41467_2025_67453_MOESM2_ESM.pdf]

nature portfolio

Corresponding author(s): Thomas Sudhof
Jie Wang

Last updated by author(s): Sep 16, 2025

Reporting Summary

Nature Portfolio wishes to improve the reproducibility of the work that we publish. This form provides structure for consistency and transparency in reporting. For further information on Nature Portfolio policies, see our Editorial Policies and the Editorial Policy Checklist.

Statistics

For all statistical analyses, confirm that the following items are present in the figure legend, table legend, main text, or Methods section.

n/a	Confirmed	
☐	☒	The exact sample size (*n*) for each experimental group/condition, given as a discrete number and unit of measurement
☐	☒	A statement on whether measurements were taken from distinct samples or whether the same sample was measured repeatedly
☐	☒	The statistical test(s) used AND whether they are one- or two-sided<br>*Only common tests should be described solely by name; describe more complex techniques in the Methods section.*
☐	☒	A description of all covariates tested
☐	☒	A description of any assumptions or corrections, such as tests of normality and adjustment for multiple comparisons
☐	☒	A full description of the statistical parameters including central tendency (e.g. means) or other basic estimates (e.g. regression coefficient) AND variation (e.g. standard deviation) or associated estimates of uncertainty (e.g. confidence intervals)
☐	☒	For null hypothesis testing, the test statistic (e.g. *F*, *t*, *r*) with confidence intervals, effect sizes, degrees of freedom and *P* value noted<br>*Give P values as exact values whenever suitable.*
☒	☐	For Bayesian analysis, information on the choice of priors and Markov chain Monte Carlo settings
☒	☐	For hierarchical and complex designs, identification of the appropriate level for tests and full reporting of outcomes
☒	☐	Estimates of effect sizes (e.g. Cohen's *d*, Pearson's *r*), indicating how they were calculated

Our web collection on statistics for biologists contains articles on many of the points above.

Software and code

Policy information about availability of computer code

Data collection	Confocal images were taken by a Nikon A1RSi confocal microscopy. STED images were taken by a Nikon Ti2-E microscope stand equipped with a STEDYCON confocal and STED module from Abberior Instruments, Inc. Electrophysiological recordings were performed on a fixed stage inverted phase-contrast microscope (Olympus). Quantitative RT-PCRs were performed using the QuanStudio3 Real-Time PCR Systems (Thermo Fisher Scientific). Protein size exclusion chromatography was performed using AKTA pure (Cytiva). Cell aggregation assay images were taken by a Zeiss Cell Discoverer 7 Automated Microscope.
Data analysis	For the quantification of axonal and dendritic growth, data were analyzed using the Simple Neurite Tracer (SNT) plugin in Fiji software. Synaptic puncta in confocal images were quantified with Nikon NIS-Elements software (Nikon). Electrophysiological recordings were analyzed offline using Clampfit 10.2 (Molecular Devices). BAI3 protein surface expression was quantified with Fiji software. Puncta in STED images were analyzed using Huygens Software and Nikon NIS-Elements. Data from the cell aggregation assay, cell surface labeling assay, and genotyping gel band intensity were also analyzed with Fiji software. Lentiviral transduction efficiency in neurons and glia was quantified using Nikon NIS-Elements software. All representative images were processed with Fiji software, and statistical analyses were performed using GraphPad Prism 9 or 10. Protein size exclusion chromatography data were exported from UNICORN (Cytiva) and plotted using GraphPad Prism 9 software.

For manuscripts utilizing custom algorithms or software that are central to the research but not yet described in published literature, software must be made available to editors and reviewers. We strongly encourage code deposition in a community repository (e.g. GitHub). See the Nature Portfolio guidelines for submitting code & software for further information.

Data

Policy information about availability of data

All manuscripts must include a data availability statement. This statement should provide the following information, where applicable:

- Accession codes, unique identifiers, or web links for publicly available datasets
- A description of any restrictions on data availability
- For clinical datasets or third party data, please ensure that the statement adheres to our policy

All raw data of the study have been deposited in the Stanford Data Repository . Source data are provided with this paper. This paper does not report original code.

Research involving human participants, their data, or biological material

Policy information about studies with human participants or human data. See also policy information about sex, gender (identity/presentation), and sexual orientation and race, ethnicity and racism.

Reporting on sex and gender	N/A
Reporting on race, ethnicity, or other socially relevant groupings	N/A
Population characteristics	N/A
Recruitment	N/A
Ethics oversight	N/A

Note that full information on the approval of the study protocol must also be provided in the manuscript.

Field-specific reporting

Please select the one below that is the best fit for your research. If you are not sure, read the appropriate sections before making your selection.

[x] Life sciences [ ] Behavioural & social sciences [ ] Ecological, evolutionary & environmental sciences

For a reference copy of the document with all sections, see nature.com/documents/nr-reporting-summary-flat.pdf

Life sciences study design

All studies must disclose on these points even when the disclosure is negative.

Sample size	Sample sizes are shown in the figure legends or figures and were determined based on historical practices in the lab (PMID: 31985401, PMID: 36997523) to claim statistical effects. We did not use a power analysis or other statistical methods to predetermine the sample size.
Data exclusions	No data was excluded from the analyses.
Replication	Efforts were made to incorporate variation by sampling from different litters and independent biological replicates. Key phenotypes were reproduced across different measurement types by independent experimenters working blindly (electrophysiology, confocal microscopy imaging, and super-resolution STED imaging).
Randomization	Allocation was random.
Blinding	All the imaging and electrophysiology experiments were performed blind to genotype or treatment. The only unblinded experiments were the size-exclusion chromatography experiments (Extended Data Fig. 1), the qRT-PCR quantification of mRNA levels (Extended Data Fig. 3d) and genotyping (Extended Data Fig. 4c), as they could be performed directly without the risk of experimental bias.

Reporting for specific materials, systems and methods

We require information from authors about some types of materials, experimental systems and methods used in many studies. Here, indicate whether each material, system or method listed is relevant to your study. If you are not sure if a list item applies to your research, read the appropriate section before selecting a response.

Materials & experimental systems

n/a	Involved in the study
[ ]	[x] Antibodies
[ ]	[x] Eukaryotic cell lines
[x]	[ ] Palaeontology and archaeology
[ ]	[x] Animals and other organisms
[x]	[ ] Clinical data
[x]	[ ] Dual use research of concern
[x]	[ ] Plants

Methods

n/a	Involved in the study
[x]	[ ] ChIP-seq
[x]	[ ] Flow cytometry
[x]	[ ] MRI-based neuroimaging

Antibodies

Antibodies used

These antibodies were used for confocal microscopy imaging at the indicated dilution ratios:
1. vGlut1 (guinea pig, Millipore, AB5905, 1:1000)
2. Homer1 (rabbit, Millipore, ABN37, 1:500)
3. MAP2 (chicken, Encor, #CPCA-MAP2, 1:1000)
4. vGAT (guinea pig, Synaptic Systems, 131004, 1:1000)
5. Gephyrin (mouse, Synaptic Systems, 147111, 1:500)
6. HA (mouse, Covance, MMS101R, 1:1000)
7. GFAP (mouse, Millipore, MAB360, 1:1000)
8. HA (rabbit, Cell Signaling Technologies, 3724, 1:1000)
9. FLAG (rabbit, Sigma, F7425, 1:500)
10. NeuN (rabbit, Millipore, ABN78, 1:1000)
11. GFP (chicken, Aves Labs, GFP-1020, 1;1000)
12. FLAG (mouse, Sigma, F3165, 1:500)
13. Goat anti-Guinea Pig IgG (H+L) Secondary Antibody, Alexa Fluor™ 546 (Thermo Fisher Scientific, A-11074, 1:1500)
14. Goat anti-Guinea Pig IgG (H+L) Secondary Antibody, Alexa Fluor™ 488 (Thermo Fisher Scientific, A-11073, 1:1500)
15. Goat anti-Rabbit IgG (H+L) Highly Cross-Adsorbed Secondary Antibody, Alexa Fluor™ 647 (Thermo Fisher Scientific, A-21245, 1:1500)
16. Goat anti-Chicken IgY (H+L) Secondary Antibody, Alexa Fluor™ 488 (Thermo Fisher Scientific, A-11039, 1:1500)
17. Goat anti-Chicken IgY (H+L) Secondary Antibody, Alexa Fluor™ 546 (Thermo Fisher Scientific, A-11040, 1:1500)
18. Goat anti-Mouse IgG (H+L) Highly Cross-Adsorbed Secondary Antibody, Alexa Fluor™ 647 (Thermo Fisher Scientific, A-21236, 1:1500)
19. Goat anti-Rabbit IgG (H+L) Cross-Adsorbed Secondary Antibody, Alexa Fluor™ 546 (Thermo Fisher Scientific, A-11010, 1:1500)
20. Goat anti-Mouse IgG (H+L) Highly Cross-Adsorbed Secondary Antibody, Alexa Fluor™ 488 (Thermo Fisher Scientific, A-11029, 1:1500).

These antibodies were used for STED microscopy imaging at the indicated dilution ratios:
1. HA (mouse, Covance, MMS101R; 1:1,000)
2. Homer1 (guinea pig, Synaptic Systems, 160004, 1:500)
3. vGlut1 (rabbit, Yenzym, 6089, 1:500)
4. Abberior STAR 460L, goat anti-mouse IgG (abberior, ST460L-1001, 1:500)
5. Abberior STAR RED, goat anti-guinea pig IgG (abberior, STRED-1006, 1:500)
6. Abberior STAR ORANGE, goat anti-rabbit IgG (abberior, STORANGE-1002, 1:500)

Validation

Validation information for antibodies used in confocal microscopy imaging:
1. vGlut1 (guinea pig, Millipore, AB5905, 1:1000), validated by the vendor, and used by papers (PMID: 37591863, PMID: 33646123).
2. Homer1 (rabbit, Millipore, ABN37, 1:500), validated by the vendor, and used by reference paper (PMID: 34913963).
3. MAP2 (chicken, Encor, #CPCA-MAP2, 1:1000), validated by the vendor and used by papers (PMID: 34913963, PMID: 33646123).
4. vGAT (guinea pig, Synaptic Systems, 131004, 1:1000), validated by the vendor in knockout mice.
5. Gephyrin (mouse, Synaptic Systems, 147111, 1:500), validated by the vendor in knockout mice.
6. HA (mouse, Covance, MMS101R, 1:1000), validated by the vendor, and used by reference paper (PMID: 30792275).
7. GFAP (mouse, Millipore, MAB360, 1:1000), validated by the vendor, and used by reference paper (PMID: 31883794).
8. HA (rabbit, Cell Signaling Technologies, 3724, 1:1000), reference papers (PMID: 36997523, PMID: 31262725).
9. FLAG (rabbit, Sigma, F7425, 1:500), reference papers (PMID: 28297718, PMID: 32504010)
10. NeuN (rabbit, Millipore, ABN78, 1:1000), reference papers (PMID: 31996670, PMID: 29398364)
11. GFP (chicken, Aves Labs, GFP-1020, 1;1000), reference papers (PMID: 39708809, PMID: 39117691)
12-19. All secondary antibodies were extensively validated by vendors and previous papers from the lab.

Validation information for antibodies used for STED microscopy imaging:
1. HA (mouse, Covance, MMS101R, 1:1000), validated by the vendor, and used by reference paper (PMID: 30792275).
2. Homer1 (guinea pig, Synaptic Systems, 160004, 1:500), validated by the vendor, and used by reference paper (PMID: 31262725).
3. vGlut1 (rabbit, Yenzym, 6089, 1:500), validated by the vendor, and used by reference paper (PMID: 35420982).
4. Abberior STAR 460L, goat anti-mouse IgG (abberior, ST460L-1001, 1:500), validated by the vendor, and used by reference paper

(PMID: 38684366).
5. Abberior STAR RED, goat anti-guinea pig IgG (abberior, STRED-1006, 1:500), validated by the vendor, and used by reference paper (PMID: 36997523).
6. Abberior STAR ORANGE, goat anti-rabbit IgG (abberior, STORANGE-1002, 1:500), validated by the vendor, and used by reference paper (PMID: 33472075).

Eukaryotic cell lines

Policy information about cell lines and Sex and Gender in Research

Cell line source(s)	Expi293F cells (Thermo Fisher Scientific) were cultured using Expi293 Expression Medium (Thermo Fisher Scientific). FreeStyle 293-F cells (Thermo Fisher Scientific) were grown in FreeStyle 293 Expression Medium (Thermo Fisher Scientific). Cells were cultured at 37 °C, with humidified atmosphere of 8% CO2 on a shaker at 130 rpm. Lenti-X 293T cells (Takara) and HEK 293T cells (ATCC) were cultured in Dulbecco's Modified Eagle Medium (Thermo Fisher Scientific) supplemented with 10% FBS, at 37°C, with humidified atmosphere of 5% CO2.
Authentication	None of the cell lines used were authenticated.
Mycoplasma contamination	These cells were thawed from a fresh vial every month and therefore not checked for mycoplasma contamination.
Commonly misidentified lines (See ICLAC register)	No.

Animals and other research organisms

Policy information about studies involving animals; ARRIVE guidelines recommended for reporting animal research, and Sex and Gender in Research

Laboratory animals	P0 pups including both males and females were used to make primary hippocampal culture of mixed neurons and glia. These mouse lines were used:<br>1. BAI1 constitutive knockout mice (this study). To generate BAI1 constitutive knockout mice, BAI1 knockin and cKO male mice (PMID: 34758294) were crossed with CMV-Cre (B6.C-Tg(CMV-cre)1Cgn/J, The Jackson Laboratory, #006054) female mice to delete BAI1 in all tissues. The Cre was further removed by crossing the offspring with C57BL/6J wild-type mice (The Jackson Laboratory, # 000664).<br>2. BAI2 conditional knockout mice (PMID: 25611509)<br>3. BAI3 conditional knockout mice (PMID: 25611509)<br>For genotype distribution and body weight measurement of BAI1 constitutive knockout mice, P21 offspring from heterozygous parents were used. Males and females were analyzed separately.<br>CD1 IGS mice were purchased from Charles River Laboratories.<br>All the animals were housed in the RAF2 animal facility, Room 071 at Stanford University, which is designed to provide optimal living conditions for laboratory mice. The facility maintains a controlled environment and the facility staff monitor and maintain the housing conditions.
Wild animals	No wild animal was used in this study.
Reporting on sex	Both male and female mice were used in this study.
Field-collected samples	This study does not have field-collected samples.
Ethics oversight	All animal usage was approved by Stanford IACUC, Administrative Panel on Laboratory Animal Care (APLAC) Research Compliance Office, Stanford University and followed NIH Guidelines for the Care and Use of Laboratory Animals.

Note that full information on the approval of the study protocol must also be provided in the manuscript.

Plants

Seed stocks	N/A
Novel plant genotypes	N/A
Authentication	N/A
