# Supplementary material for: BAI adhesion-GPCRs perform distinct functions in neural development differentially controlled by RTN4R and C1ql ligands
Source: Nat Commun. 2025 Dec 23;17:6140. doi: 10.1038/s41467-025-67453-6 (PMC13365561; doi:10.1038/s41467-025-67453-6)
Supplement: Supplementary file 3 — Transparent Peer Review file [file 41467_2025_67453_MOESM3_ESM.pdf]

nature portfolio

Peer Review File

BAI Adhesion-GPCRs Perform Distinct Functions in Neural Development Differentially Controlled by RTN4R and C1ql Ligands

Corresponding Author: Dr Jie Wang

This file contains all reviewer reports in order by version, followed by all author rebuttals in order by version.

Attachments originally included by the reviewers as part of their assessment can be found at the end of this file.

Version 0:

Reviewer comments:

Reviewer #1

(Remarks to the Author)
In this manuscript, Wang et al. analyse the roles of BAI adhesion-GPCRs in neuronal development, synapse formation, and function. The objective is to determine whether all three BAIs play similar roles and if their interactions with two known groups of ligands, RTN4R receptors and C1QL secreted proteins, are necessary for these functions. To achieve this, the authors utilised cultured hippocampal neurons from genetically modified mice with knockout of each BAI receptor and assessed the consequences of these loss-of-function experiments through morphological and electrophysiological approaches. They discovered that BAI1 and BAI3, but not BAI2, are involved in regulating dendritogenesis, axonogenesis, and excitatory synapse density and function, with no observed effects on inhibitory synapses. Rescue experiments further demonstrated that a BAI3 protein with mutations in the TSR2 domain could not restore any of these functions. Interestingly, removing the N-terminus of BAI3, while preserving the TSR domains, did not impact BAI3's ability to rescue the phenotypes except for its effect on synaptic transmission. The authors conclude that RTN4R binding is essential for all BAI functions, whereas binding to C1QLs is crucial only for synapse organization. This important work significantly enhances our understanding of BAI receptors, which are increasingly recognized as key regulators of brain development and function.

A main concern is the lack of biochemical characterization of the binding properties of the different BAIs and the mutant forms of BAI3 with RTN4Rs and C1QLs. The authors cite Wang et al. (Cell 2021) for the point mutations of BAI3 that are stated to abolish RTN4R binding. However, while Wang et al. experimentally verified the effects of the T424V and R430A mutations on BAI1's RTN4R binding capacity, I do not find data showing that they tested these “equivalent” mutations in BAI3. It is also crucial to experimentally determine whether these mutations affect BAI3's ability to bind C1QL3 and other C1QLs. Similarly, the authors reference Kakegawa et al. (2015) for the ΔN-BAI3 mutant. In this article, the capacity of the ΔN-BAI3 mutant to bind to C1QL1 was tested using SPR. It is important to assess whether this deletion affects binding to all C1QLs uniformly. The specificity of C1QL3 binding to the different BAIs was assessed using size-exclusion chromatography (Sup. Fig1), but C1QL3 is scarcely visible in the gels for the BAI2 and BAI3 experiments. Validating this finding using other approaches would strengthen the authors’ interpretations.

The KO models are validated using quantitative RT-PCR with probes targeting the floxed regions. Can the authors confirm that no truncated mRNA and/or protein is expressed following recombination?

On page 7, the authors mention that BAI3 mutant constructs are expressed at similar levels on the cell surface. However, the protocol for immunocytochemistry indicates the use of Triton X-100 in the incubation buffer. This means that their protocol does not exclusively label surface proteins. Is the tag on the intracellular part of the proteins? If an extracellular tag is available (Flag as stated in the material and methods section?), immunolabelling in non-permeabilizing conditions could be performed to really assess whether all constructs are expressed similarly at the cell surface.

Finally, estimating the transduction efficiency in the neuronal cultures would be beneficial for data interpretation.

Reviewer #2

(Remarks to the Author)
BAI1-3 are adhesion GPCRs involved in multiple functions in neuronal development. BAIs are known to interact with families of BAI ligands, including secreted C1qls and membrane-bound RTN4R proteins, but which of these ligands regulate specific functions of BAIs is incompletely understood. To address these questions, the authors investigated the functions of Bai1-3 in cultured hippocampal neurons using genetic knockouts and rescue experiments. They showed that the deletion of either BAI1 or BAI3, but not BAI2, increased axonal and dendritic arborization but decreased synapse formation. They focused on the role of two Bai3 ligands, C1qls and RTN4R, using rescue experiments with BAI3 mutants that cannot bind either C1qls or RTN4R. The authors demonstrated that RTN4R binding to BAI3 is essential for restricting axonal and dendritic arborization and promoting excitatory synapse formation, whereas C1ql binding to BAI3 is required only for functional synapses. Based on these results, the authors propose that the three BAI proteins perform distinct functions using two ligands.

These authors have previously identified C1qls and RTN4R as ligands for Bai proteins and analyzed their functions. I appreciate the importance of the present work, which aims to clarify how these ligands regulate neuronal functions. However, as shown below, the research methodology may not be appropriate to answer this question. In addition, the details of the analytical methods are not well described, and several important experiments are missing. Therefore, unfortunately, I cannot support the authors' conclusions based on the data currently presented.

Major points:

1. Expression of Endogenous Bai1-3 in the Hippocampus and Its Function
While studying Bai1-3 function in a reduced system such as cultured neurons is not inherently problematic, it is important to note that Bai1-3 function is dependent on the cell context. For instance, Bai3 KO mice exhibit impaired synapse formation in vivo in the olfactory bulb, cerebellum, and amygdala. However, in the hippocampus, Bai2 and Bai3 genetic KO mice demonstrate no change in the number of CA3-CA1 synapses where endogenous C1ql2 and C1ql3 are localized (Matsuda et al, Neuron 2016). Therefore, it is questionable whether cultured hippocampal neurons can be used as a model to study changes in synapse number or pyramidal cell morphology. It is also unclear whether Bai1-3 is expressed in all hippocampal neurons.
One potential avenue for investigation is to examine the function of overexpressed Bai in cultured hippocampal neurons, independent of endogenous Bai1-3 function in vivo. However, it is important to note that the results obtained may not necessarily reflect Bai1-3 function in vivo. Additionally, it is necessary to confirm that the ligands for Bai1-3 are ubiquitously expressed in cultured hippocampal neurons. For instance, the ligands for Bai3, C1ql2 and C1ql3, are specifically expressed in granule cells of the dentate gyrus, which make synapses with CA3 pyramidal neurons. However, it is unclear whether C1qls bind to Bai in cultured hippocampal neurons other than CA3 neurons. The same is true for RTN4Rs, the ligands for Bai1 and Bai3. In this paper, the authors utilized Bai with mutations in the binding sites for C1qls and RTN4Rs, but it remains unclear whether and how these ligands are expressed in cultured hippocampal neurons. To address these concerns, the authors should clarify which neurons and synapses express BAI1-3 ligands in cultured neurons. It would also be reassuring if neurons cultured from Bai ligand (e.g., C1qls or RTN4Rs) KO mice show the same phenotypes as neurons expressing Bai3 mutants.

2. Elongation of Dendrite/Axon and Synaptic Density
It is not surprising that synaptic density per unit length increases when the length of dendrites and axons decreases. Indeed, in Bai1 KO neurons, which are unable to bind C1qls (this study), and in RTN4R KO neurons (Wang et al., Cell 2021), the length of dendrites and axons increased and synaptic density decreased. It can be postulated that the fundamental function of Bai1/3-RTN4R signaling may be to inhibit axon and dendrite length, rather than to promote synaptogenesis. It is therefore not surprising that Bai3-dN, which is unable to bind to C1ql, can still rescue the reduction in synapse number observed in Bai3 KOs.
As previously stated, I believe that cultured hippocampal neurons may not be an optimal model for assessing the synaptogenic potential of Bai. However, if the synaptogenic potential of Bai overexpression is to be evaluated in hippocampal neurons, the authors should assess the number of synapses as well as spine morphology, rather than synaptic density, by sparse KO + rescue experiments as discussed below.

3. Experimental Methods for KO-Rescue
In a dissociated primary culture, the maturation and number of synapses are significantly influenced by the density of surrounding neurons and glia. For instance, as illustrated in Fig. 5a, the density of neurons appears to be relatively high for the third (Cre+WT) and fifth (Cre +dN) panels, which may influence the synaptic density. Furthermore, the authors' model (Wang et al., Cell 2021) indicates that RTN4Rs expressed on neuronal axons inhibit axon outgrowth by interacting with Bai in glial cells in trans. Consequently, dendrite outgrowth may be influenced in neurons lacking Bai, whereas axon outgrowth should remain unaltered if the surrounding glia is wild-type expressing Bai. To circumvent this issue, it would be better to sparsely express the Cre and rescue constructs and then compare them to neighboring wild-type neurons in the same culture dish. To evaluate the role of glial Bai, the authors should utilize glia-specific promoters to induce Cre expression.
A related question is the efficiency of the knockout and rescue processes. Cre was expressed by lentivirus at 4DIV, but it is unclear what percentage of neurons and glia are actually knocked out with Bai2, 3. In Supplement Fig. 4a, it appears that HA-BAI3 rescued by lentivirus is not expressed in all neurons. It is not described how the authors ensured that the neurons being evaluated for electrophysiology and morphology have been successfully knocked out or knocked out and rescued for BAI. For instance, in Supplemental Figure 4a, MAP2 (magenta) is utilized to identify dendrites, and HA (green) is employed for rescue constructs. However, it is also described as magenta was used for Cre.

4. The Function of Bai3 Regulated by C1qL
The authors propose that C1qL binding to Bai3 is required for synapse “organization,” not synaptogenesis, because Bai3 dN can rescue the reduction in synapse number of Bai3 KOs (Fig. 5b) but it cannot rescue the reduction in mEPSCs (Fig. 6b). However, the specific nature of synapse “organization” remains unclear. If glutamate release from the presynapse (Fig. 6e) is not affected, the authors must examine whether there are abnormalities in the number or arrangement of postsynaptic AMPA receptors.

5. Quantitative Methods for Synapses and Synaptic Molecules
The methods for quantifying synaptic morphology and synaptic molecules are not well described. Specifically, authors need to describe how they determined the brightness and size of the puncta of synaptic molecules. It is also unclear how synapses were defined, and the criteria used to select dendrites for measurement of synaptic density, including details such as distance from the cell body and apical/basal dendrites.
In Fig. 7, the synaptic localization of Bai3 is examined, yet the method utilized for quantification is not well described. For instance, in Fig. 7a, the puncta exhibit a range of sizes, and it is not evident which ones represent pre- and postsynaptic or glial cells. Additionally, in the merged image in Fig. 7, it is not possible to distinguish which molecules are co-localized by color. For example, it is not possible to distinguish between the following combinations because the merged images will become white in all cases:
- (1) Cyan (HA) + Yellow (Homer): merge [Homer having BAI]
- (2) Cyan (HA) + Red (vGluT1): merge [vGluT1 having BAI]
- (3) Cyan (HA) + Yellow (Homer) + Red (vGluT1): merge [Synaptic BAI]
In addition, Homer alone (yellow) is indistinguishable from synapses (yellow (Homer) + red (vGluT1)).
Upon closer examination of the image, the majority of HA-BAI3 signals do not appear to overlap the "synapse." This does not align with the assertion (page 9, line 280) that "the majority of transfected BAI3 is in the synapse." Additionally, the signals that overlap VGluT1 and Homer are relatively small and do not align with the quantitative results shown in Fig. 7f, g. It is recommended that the pseudocolor of the image be altered to demonstrate the degree of overlap between the colors, and that the quantification method be described or reexamined. The authors also need to show enlarged views of synapses.

6. Binding of Bai2 to C1qls
The authors assert that C1qls bind “weakly” to Bai2 (lines 129-130; 312-313) based on the size-exclusion chromatography, showing that the N-terminal domain (N-TSR4) of Bai2 and the globular domain of C1ql3 co-migrate slightly. However, the term "weakly" should not be used without assessing the Kd value. It is also not a fair comparison to use the full length of the extracellular domain for Bai1 and Bai3, but only a part of it for Bai2. Given that Bai2 lacks a CUB domain, which is required for Bai1 or Bai3 to bind to C1qls, the authors must carefully examine the interaction between full-length C1qls (not only the globular domain) and Bai2. In addition, the term "C1qls" is not accurate since only C1ql3 is utilized as a ligand, yet no other C1ql is subjected to testing. Furthermore, despite the authors' assertion that "It is unclear whether C1qls bind to other BAIs besides Bai3" (line 120), C1ql1 was previously demonstrated to lack binding affinity for Bai1 or Bai2 in cell-based binding assays (Matsuda et al, Neuron, 2016).

Minor points:
1. In Figures 3 and 6, the results of cumulative frequency plots of mEPSC and mIPSC demonstrate notable differences between WT and KO. However, these findings are not explicitly addressed in the text, which may lead to confusion among readers. For instance, Fig. 3 illustrates a significant difference in mEPSC amplitude between WT and Bai1 KO. Additionally, there is a significant difference in mIPSC frequency between WT and Bai1, Bai2 KO.

2. In Fig. 6, the size of evoked EPSCs reaches 4 nA, which is considerably larger than what would be expected in hippocampal dissociated culture. Please verify that there are no mistakes.

3. Membrane capacitance is a sensitive measure of surface area, which is directly related to the elongation of dendrites and axons. However, the text states that "there is no difference in membrane capacitance or input resistance in BAI-deficient cells" (lines 189-192). Please verify the data.

Reviewer #3

(Remarks to the Author)
In this manuscript, Wang et al. utilize mouse hippocampal cultures to investigate the roles of BAI adhesion-GPCRs and their known ligand interactions in hippocampal neuronal development. In agreement with previous studies, the authors confirm that BAI1 and BAI3 restrict axonal and dendritic growth and promote excitatory synapse formation, whereas the authors do not detect a role for BAI2 in these processes. The authors also examine the ligand-binding properties of BAIs, verifying that secreted C1ql proteins interact with both BAI3 and BAI2 (but not BAI1), in contrast to GPI-linked RTN4Rs, which the group previously reported bind to BAI1 and BAI3 (but not BAI2). Using rescue experiments, the authors further show that RTN4R-binding to BAI3 is essential for both limiting axonal/dendritic arborization and enhancing synapse formation, while C1ql-binding to BAI3 is only required for regulating excitatory synapse organization/ function. Based on these findings, the authors conclude that a dual ligand-binding mechanisms controls BAI3's neurodevelopmental functions, with BAI3-RTN4R binding required for axonal/dendritic growth regulation and both RTN4R- and C1ql-binding essential for BAI3-mediated synapse assembly.

Overall, this is an interesting study that extends our understanding of the mechanisms by which BAI adhesion-GPCRs regulate neurodevelopment. The manuscript is well-written, and the data presented are clear, thorough, and generally

convincing. While some of the results are confirming previously reported functions for BAIs and their ligands, this manuscript comprehensively assesses all three BAI family members in hippocampal neurons and compares the specific roles of BAI3's two known ligands. To further strengthen this manuscript, however, the following issues should be addressed.

Major concerns:

* In Figure S1, the authors compare the ability of BAI1, BAI2 and BAI3 to interact with C1ql3. Why was a different extracellular fragment of BAI2 used instead of the directly analogous fragment used for BAI1/3? The authors could also provide additional information in the figure legend, clarifying what is shown in the size-exclusion chromatography and Coomassie-stained SDS-PAGE gel in terms of BAI binding to C1ql3 (note shifts showing positive interactions).

* Figure 3 is a bit confusing. While the summary bar graphs agree with the text, the cumulative fraction plots of the inter-event intervals and peak amplitudes report statistical differences that appear to contradict the text and the bar graphs. These are not mentioned in the text. Can this result be further explained? Is a distinction being drawn between mEPSC amplitude and peak amplitude, for example? What is being added by these histograms and why aren't they discussed in the text? What comparisons are being denoted in these graphs? This same issue applies to Figure 6.

* In Figures 4/S4, the authors show that ligand-binding mutations in BAI3 reduce (but do not eliminate) BAI3's synaptic localization. However, based on the staining method used, it remains unclear whether the BAI3 mutations affect cell surface localization. An addition live-staining experiment with intact plasma membrane could clarify this issue.

* The authors note that their results regarding the C1ql-BAI3 interaction are distinct from those previously reported by themselves and others (only required for synaptic function, not formation). Moreover, the authors do not identify a role for BAI2 in either axonal/dendritic growth or synapse development. However, the authors only performed their experiments in hippocampal mouse cultures. It is important to stress in the discussion that the findings in this paper may be specific for hippocampal neurons and that neurons in different brain regions may be differentially regulated by the BAI family members.

* While the authors acknowledge in the discussion that they did not distinguish between glial and neuronal BAIs, they previously argued that glial BAI1/3 restrict axonal/dendritic growth while neuronal BAI1/3 regulate excitatory synaptogenesis (and they allude to this in their model figure (Figure 8)). Though they show in Figure S4 that they can express BAI3 constructs in both neurons and astrocytes, it would be informative to provide some quantification of the extent of BAI1/2/3 loss and BAI3 rescue construct expression in astrocytes vs neurons in their hippocampal cultures. This issue could also be addressed by independently manipulating BAI levels in neurons vs astrocytes.

* On lines 198 to 202, the authors argue that "the total number of synapses in the BAI1- and BAI3-deficient neurons is likely not changed given that these neurons exhibit an increase in dendritic and axonal growth but a decrease in synapse density per dendritic length. In view of this synapse number constancy, the decrease in synaptic mEPSCs is thus remarkable, suggesting that the BAI1 and BAI3 deletions also impair synaptic strength and not only synapse density". If this is the case, why is mEPSP amplitude not affected? To show that synapse constancy is indeed achieved, the authors need to analyze different domains of the neurons (primary, secondary, tertiary dendrites). If this was done, the methods should make this clear.

* The authors infected hippocampal cultures from BAI3 cKO mice with lentiviruses expressing Cre on DIV4, infected rescue lentiviruses at DIV6, and sparsely transfected neurons with tdTomato at DIV9. Did the authors confirm that the neurons they analyzed were affected by all 3 manipulations? Please clarify?

* Different KO models were used for BAI1, BAI2, and BAI3. The deletion of BAI1 is constitutive while the deletion of BAI2 and BAI3 are more acute. Could this contribute to the different results seen in punctal size analysis between BAI1 and BAI3 shown in Figure 2 (i.e. compensation in the BAI1 model)?

Minor concerns:

* Fig. 5 (page 8). If the decrease in excitatory synapses in the ΔN BAI3 mutant is "slight" and "statistically insignificant", then perhaps it is more accurate to say that there is no decrease in excitatory synapses in this condition.

Version 1:

Reviewer comments:

Reviewer #1

(Remarks to the Author)
The authors have addressed most of my concerns and the data presented are really interesting.
There is only one minor concern left regarding the potential presence of a truncated protein in the KO condition.
I agree with the authors that given the design of the BAI1 KO, one can be confident that no protein that could have a functional impact is produced. In the case of the BAI3 KO condition, it is less clear. The authors refer to the Kakegawa et al. (2015) paper. In that article, the authors document the KO via immunolabeling of tissue sections with a homemade anti-Bai3 antibody. I couldn't find any information on the characterization. of this antibody and immunolabeling does not test whether a

truncated protein is produced. If a good antibody against BAI3 for immunoblotting is unavailable to the authors, then at least a qRT-PCR using a probe outside of the deleted region might be helpful to measure how much truncated mRNA is produced (the mRNA from the mutated allele might be subject to nonsense-mediated decay which would be good).

Reviewer #3

(Remarks to the Author)
I am satisfied that the authors successfully addressed all my concerns. I believe it is a strong, interesting manuscript worthy of publication in Nature Communications.

Open Access This Peer Review File is licensed under a Creative Commons Attribution 4.0 International License, which permits use, sharing, adaptation, distribution and reproduction in any medium or format, as long as you give appropriate credit to the original author(s) and the source, provide a link to the Creative Commons license, and indicate if changes were made.
In cases where reviewers are anonymous, credit should be given to 'Anonymous Referee' and the source.
The images or other third party material in this Peer Review File are included in the article’s Creative Commons license, unless indicated otherwise in a credit line to the material. If material is not included in the article’s Creative Commons license and your intended use is not permitted by statutory regulation or exceeds the permitted use, you will need to obtain permission directly from the copyright holder.
To view a copy of this license, visit https://creativecommons.org/licenses/by/4.0/

Reviewer Comments and Authors' Responses for Wang et al., "BAI Adhesion-GPCRs Perform Distinct Functions in Neural Development Differentially Controlled by RTN4R and C1ql Ligands"

We thank the reviewers for their careful and constructive comments. In light of these comments, we have now performed additional experiments and amended the paper as described in detail below. We apologize for the length of time it has taken us to resubmit this paper, which was due to the extensive nature of the additional experiments that were recommended by the reviewers and to the move of two of the authors, including the first author to new independent positions. As described in detail below, we have tried to address all of the reviewers' comments. We thus hope that the revised manuscript can be deemed acceptable for publication. In the following, we cite the reviewers' comments in full in *italic* typeface and then provide our response in blue typeface. We numbered the comments of reviewers 1 and 3 to facilitate their discussion.

Reviewer #1

In this manuscript, Wang et al. analyse the roles of BAI adhesion-GPCRs in neuronal development, synapse formation, and function. The objective is to determine whether all three BAIs play similar roles and if their interactions with two known groups of ligands, RTN4R receptors and C1QL secreted proteins, are necessary for these functions. To achieve this, the authors utilised cultured hippocampal neurons from genetically modified mice with knockout of each BAI receptor and assessed the consequences of these loss-of-function experiments through morphological and electrophysiological approaches. They discovered that BAI1 and BAI3, but not BAI2, are involved in regulating dendritogenesis, axonogenesis, and excitatory synapse density and function, with no observed effects on inhibitory synapses. Rescue experiments further demonstrated that a BAI3 protein with mutations in the TSR2 domain could not restore any of these functions. Interestingly, removing the N-terminus of BAI3, while preserving the TSR domains, did not impact BAI3's ability to rescue the phenotypes except for its effect on synaptic transmission. The authors conclude that RTN4R binding is essential for all BAI functions, whereas binding to C1QLs is crucial only for synapse organization. This important work significantly enhances our understanding of BAI receptors, which are increasingly recognized as key regulators of brain development and function.

We appreciate the reviewer's concise and accurate summary of our results and constructive comments that have been very helpful in our revision.

1. A main concern is the lack of biochemical characterization of the binding properties of the different BAIs and the mutant forms of BAI3 with RTN4Rs and C1QLs. The authors cite Wang et al. (Cell 2021) for the point mutations of BAI3 that are stated to abolish RTN4R binding. However, while Wang et al. experimentally verified the effects of the T424V and

R430A mutations on BAI1's RTN4R binding capacity, I do not find data showing that they tested these “equivalent” mutations in BAI3. It is also crucial to experimentally determine whether these mutations affect BAI3's ability to bind C1QL3 and other C1QLs. Similarly, the authors reference Kakegawa et al. (2015) for the ΔN-BAI3 mutant. In this article, the capacity of the ΔN-BAI3 mutant to bind to C1QL1 was tested using SPR. It is important to assess whether this deletion affects binding to all C1QLs uniformly. The specificity of C1QL3 binding to the different BAIs was assessed using size-exclusion chromatography (Sup. Fig1), but C1QL3 is scarcely visible in the gels for the BAI2 and BAI3 experiments. Validating this finding using other approaches would strengthen the authors' interpretations.

We agree. To address this concern, we have conducted extensive systematic binding measurements for all C1ql isoforms (C1ql1-4) and all BAI isoforms (BAI1-3) as well as BAI3 mutants, as recommended by the reviewer. The new data are presented in Extended Data Figures 2 and 6. The results confirm that BAI3, but not BAI1, bind to all C1ql isoforms. Moreover, we show that the ΔN mutant of BAI3 disrupts binding C1ql binding, whereas the T360V and R366A mutants of BAI3 do not affect C1ql binding. Finally, we confirm that the T360V and R366A mutants of BAI3 are unable to bind to RTN4R. Note that we recently published the cryo-EM structure of the BAI3-NTD complex with the trimeric C1q-like domains of C1ql3 (Y. Miao et al., Comm. Biol. 2025). This structure predicts that the BAI1 NTD will not bind to C1qls because key contact residues are not conserved, whereas the BAI2 NTD is predicted to weakly bind to C1qls, which is fully consistent with the present results.

2. The KO models are validated using quantitative RT-PCR with probes targeting the floxed regions. Can the authors confirm that no truncated mRNA and/or protein is expressed following recombination?

We entirely concur that in genetic KO experiments it is necessary to assess the possibility that truncated proteins are produced. Such proteins might cause functional interferences, for example by acting as dominant-negative or dominant-positive agents. Since no good commercial antibodies to BAI proteins are available, we carefully designed our experiments to exclude this possibility, building on studies performed by others that are cited in the manuscript.

Specifically, all of the three BAI KO mouse models we use were designed in a manner that an upstream out-of-frame exon is deleted. As a result of these deletions, mRNAs are produced that either lack the sequences containing the initiator methionine codon and the entire N-terminus including the signal peptide (BAI1; our design) or that shift the reading frame at the 5' end of the mRNAs (BAI2 and BAI3 mice; designed by the Yuzaki lab; Kakegawa et al., 2015) (ED Figure 3a-c). We confirmed the genetic designs by genotyping and demonstrated by qRT-PCR that the BAI mRNAs that are produced by the mutant genes lack the deleted exons and cause a frame shift (ED Figure 3d).

The BAI1 gene deletion we designed deletes the entire N-terminal sequences including the initiator methionine and signal peptide. Thus, if some downstream methionine is used as an alternative translation initiator, the mutant BAI1 gene cannot produce a secreted truncated protein because no signal peptide is available. This was confirmed by genotyping and by RT-PCR. The BAI2 and BAI3 conditional KO mice were constructed by the Yuzaki lab in Japan, cause extensive out-of-frame deletions, and were also validated by the Yuzaki laboratory in elegant and extensive experiments that we cite in the paper (see Kakegawa, W. *et al.*, *Neuron* 2015). Given that these mice produce at best short, truncated proteins containing partial domains, have been used in multiple publications, and are well characterized, we hope that the reviewer will agree that a further validation of the KO strategy of these mice from the Yuzaki lab is not necessary.

3. On page 7, the authors mention that BAI3 mutant constructs are expressed at similar levels on the cell surface. However, the protocol for immunocytochemistry indicates the use of Triton X-100 in the incubation buffer. This means that their protocol does not exclusively label surface proteins. Is the tag on the intracellular part of the proteins? If an extracellular tag is available (Flag as stated in the material and methods section?), immunolabelling in non-permeabilizing conditions could be performed to really assess whether all constructs are expressed similarly at the cell surface.

We agree. We have now conducted additional experiments as shown in the updated Fig. 4b, 4c, using the approach suggested by the reviewer. We performed surface staining of non-permeabilized cells with anti-Flag antibodies, followed by permeabilization and MAP2 staining, and quantified the intensity ratio of Flag to MAP2. The results confirm our prior conclusions, thus solidifying the results.

4. Finally, estimating the transduction efficiency in the neuronal cultures would be beneficial for data interpretation.

Again we agree. We have now quantified the percentage of cells expressing the lentiviruses by measuring the percentage of NeuN+ neuronal nuclei and NeuN- glial nuclei (identified by DAPI staining) that express the EGFP-Cre or EGFP-ΔCre fusion proteins, which are encoded by the lentiviruses. This allowed us to determine the proportion of neurons and glia that were infected and produced the Cre- or ΔCre-EGFP fusion proteins driven by the synapsin promoter (which is also active in glial cells). Our findings indicate that >97% of neurons and ~80% of glia were transduced (Extended Data Fig. 4a, 4b). Since a significant fraction of the glia are microglia that are poorly transduced by lentiviruses, the transduction efficiency of astrocytes and OPCs is also likely >90%.

In addition, we quantified the extent of gene recombination following ΔCre vs. Cre expression in BAI3 conditional knockout (cKO) cultures using genomic PCR. We observed an approximate recombination rate of 88% (Extended Data Fig. 4c, 4d). Again, since

microglia are poorly infected by lentiviruses, this also translates into a recombination rate of >90% not counting microglia.

Reviewer #2

BAI1-3 are adhesion GPCRs involved in multiple functions in neuronal development. BAIs are known to interact with families of BAI ligands, including secreted C1qls and membrane-bound RTN4R proteins, but which of these ligands regulate specific functions of BAIs is incompletely understood. To address these questions, the authors investigated the functions of Bai1-3 in cultured hippocampal neurons using genetic knockouts and rescue experiments. They showed that the deletion of either BAI1 or BAI3, but not BAI2, increased axonal and dendritic arborization but decreased synapse formation. They focused on the role of two Bai3 ligands, C1qls and RTN4R, using rescue experiments with BAI3 mutants that cannot bind either C1qls or RTN4R. The authors demonstrated that RTN4R binding to BAI3 is essential for restricting axonal and dendritic arborization and promoting excitatory synapse formation, whereas C1ql binding to BAI3 is required only for functional synapses. Based on these results, the authors propose that the three BAI proteins perform distinct functions using two ligands.

These authors have previously identified C1qls and RTN4R as ligands for Bai proteins and analyzed their functions. I appreciate the importance of the present work, which aims to clarify how these ligands regulate neuronal functions. However, as shown below, the research methodology may not be appropriate to answer this question. In addition, the details of the analytical methods are not well described, and several important experiments are missing. Therefore, unfortunately, I cannot support the authors' conclusions based on the data currently presented.

We appreciate the reviewer's careful assessment of our study. Basically, the reviewer expresses two principal reservations.

First, the reviewer questions the reductionist approach (the 'research methodology') of our project. He/she appears to argue that the use of hippocampal cultures is inappropriate for interrogating the differential functions of BAI1-3 and for analyzing the fundamental structure-function relationship of BAI3. As we will explain below, we hope to convince the reviewer that hippocampal cultures as a reductionist approach are not only an appropriate, but in many ways the only approach to address the basic questions we are posing. Neuron-glia cultures enable examination of fundamental neuronal cell biology, which is extremely difficult and resource-draining to do *in vivo*. We hope that after consideration the reviewer might agree.

Second, the reviewer expresses specific methodological concerns that we have addressed in detail below. We believe we have now explained the validity of each method and hope that the reviewer will also be satisfied with our explanations.

Because many of the reviewer's points include multiple different criticisms, we have subdivided these points into separate sections in order to facilitate discussion.

Major points:

1a. Expression of Endogenous Bai1-3 in the Hippocampus and Its Function

While studying Bai1-3 function in a reduced system such as cultured neurons is not inherently problematic, it is important to note that Bai1-3 function is dependent on the cell context. For instance, Bai3 KO mice exhibit impaired synapse formation in vivo in the olfactory bulb, cerebellum, and amygdala. However, in the hippocampus, Bai2 and Bai3 genetic KO mice demonstrate no change in the number of CA3-CA1 synapses where endogenous C1ql2 and C1ql3 are localized (Matsuda et al, Neuron 2016). Therefore, it is questionable whether cultured hippocampal neurons can be used as a model to study changes in synapse number or pyramidal cell morphology.

If we understand correctly, the reviewer's comment seems to state that it is questionable to study synapses and pyramidal cell morphology in hippocampal cultures because the function of BAIs is dependent on their cell context. He/she cites as evidence an excellent paper that we also discussed in our manuscript (Matsuda et al., 2016), stating that it shows that BAI2 and BAI3 KO mice do not have a change in the number of CA3-CA1 synapses (Schaffer-collateral synapses) and that endogenous C1ql2 and C1ql3 are localized to these synapses. However, in our understanding Matsuda et al. examine dentate gyrus to CA3 synapses, where they do find a major phenotype, not CA3-CA1 synapses. Moreover, they show that CA3-CA1 synapses likely contain no C1ql2 and C1ql3 (confirmed with our new Figure 8 demonstrating the C1ql2 and C1ql3 expression patterns in mice). In hippocampal cultures as a reduced system, furthermore, CA3 neurons and synapses formed by CA3 neurons are likely only a minor component. Dentate gyrus neurons and their synapses, conversely, are likely a major component. Thus, we are not convinced that cultures are an inappropriate system to study the fundamental cell biology of neurons, as documented by thousands of outstanding papers published by hundreds of laboratories, although we completely agree that the interpretation of such studies is limited to neuronal cell biology and is not informative about circuits.

1b. It is also unclear whether Bai1-3 is expressed in all hippocampal neurons.

We agree that this point needed to be addressed. To do so, we have now analyzed single-cell RNAseq data published by the Allen Brain Institute (new Figure 8) and single-nuclei RNAseq data published by the Broad Institute (new ED Figure 10). The results show that, in the hippocampus, BAI1 and BAI3 are expressed in all excitatory and inhibitory neurons at high abundance (approximately half the levels of MAP2). BAI2, conversely, is expressed at even higher abundance in excitatory neurons (approximately the same as MAP2) but at only low levels in inhibitory neurons. These results are now presented and discussed in the revised paper.

1c. One potential avenue for investigation is to examine the function of overexpressed Bai in cultured hippocampal neurons, independent of endogenous Bai1-3 function in vivo. However, it is important to note that the results obtained may not necessarily reflect Bai1-3 function in vivo.

We agree and share the reviewer's reservation that results obtained in culture may not necessarily reflect the full function of BAIs *in vivo* and have now explicitly mentioned this reservation.

1d. Additionally, it is necessary to confirm that the ligands for Bai1-3 are ubiquitously expressed in cultured hippocampal neurons. For instance, the ligands for Bai3, C1ql2 and C1ql3, are specifically expressed in granule cells of the dentate gyrus, which make synapses with CA3 pyramidal neurons. However, it is unclear whether C1qls bind to Bai in cultured hippocampal neurons other than CA3 neurons. The same is true for RTN4Rs, the ligands for Bai1 and Bai3. In this paper, the authors utilized Bai with mutations in the binding sites for C1qls and RTN4Rs, but it remains unclear whether and how these ligands are expressed in cultured hippocampal neurons. To address these concerns, the authors should clarify which neurons and synapses express BAI1-3 ligands in cultured neurons.

Again, we agree with the reviewer that this is an important point. To address this point, we have additionally analyzed the expression of the BAI ligands in the single-cell and single-nuclei RNAseq data as described in our response to this reviewer's Comment 1b above. A very interesting pattern of expression emerges, and we truly thank the reviewer for suggesting this analysis (new Figure 8 and ED Figure 10).

Strikingly, in the hippocampus the three RTN4R isoforms are expressed at high levels at approximately 3-fold (RTN4R), 5-fold (RTN4RL1) and 2-fold higher levels (RTN4RL2) than MAP2 in excitatory neurons. Unexpectedly, however, RTN4Rs are almost not present in inhibitory neurons. Whereas all excitatory neurons express RTN4Rs, only one minor class of inhibitory neurons (Pv-positive neurons) expresses one RTN4R (RTN4RL1) at low levels (new Figure 8). Since RTN4Rs are presynaptic, this surprising expression pattern may account for the specificity of BAI1 and BAI3 for excitatory synapses.

Different from RTN4Rs, and as the reviewer correctly notices, C1qls are expressed in a selective pattern in hippocampal neurons. Much of the C1ql expression has been reported earlier (e.g., see Matsuda *et al., Neuron 2016,* as well as our own study by Martinelli *et al., Neuron 2016*). The RNAseq analyses we now performed are consistent with these earlier results. Basically, C1ql1 is expressed only at low levels in Pv-positive interneurons that represent a small population in the hippocampus but in no other hippocampal neurons, C1ql2 is expressed at high levels (20-fold of those of MAP2) in dentate gyrus granule cells that constitute the largest population of neurons in the hippocampus but again in no other hippocampal neurons, C1ql3 is expressed at low levels in both dentate gyrus granule cells and CA1 neurons, and C1ql4 is not detectable. Although this expression pattern does confirm the cell-type specificity of C1ql expression, it also documents that in the

hippocampus, the majority of excitatory neurons expresses at least one C1ql isoform, with C1ql2 being by far the most abundant. Only CA3 neurons do not express a C1ql (see new Figure 8).

1e. It would also be reassuring if neurons cultured from Bai ligand (e.g., C1qls or RTN4Rs) KO mice show the same phenotypes as neurons expressing Bai3 mutants.

We concur that studying C1ql1-4 and RTN4R KO mice – a total of 7 mouse knockout lines in addition to the 3 BAI1-3 KO mouse lines that we systematically analyzed here – would be useful. However, such a massive experiment is simply beyond the scope of the current study. Just examining 3 lines required many years of work; adding another 7 lines is potentially a decade-long endeavor.

2a. Elongation of Dendrite/Axon and Synaptic Density
It is not surprising that synaptic density per unit length increases when the length of dendrites and axons decreases. Indeed, in Bai1 KO neurons, which are unable to bind C1qls (this study), and in RTN4R KO neurons (Wang et al., Cell 2021), the length of dendrites and axons increased and synaptic density decreased. It can be postulated that the fundamental function of Bai1/3-RTN4R signaling may be to inhibit axon and dendrite length, rather than to promote synaptogenesis.

The reviewer suggests that the decrease in synapse density in BAI1 and BAI3 KO neurons is simply a consequence of the increase in axon and dendrite length caused by the BAI1 and BAI3 KOs. He/she argues that it is thus not surprising that the BAI3-ΔN mutant can still rescue the synapse density decrease since it fully rescues the increase in axon and dendrite length.

Although the reviewer's hypothesis is plausible, two observations argue against it. First, if the synapses were simply 'diluted' by being formed on longer dendrites but the total number of synapses was unchanged, we should have seen no change in electrophysiologically measured synaptic responses. However, we observed a major reduction in responses, suggesting that the total number of active synapses is decreased. Second, the reviewer's hypothesis predicts that both excitatory and inhibitory synapse density on dendrites should be decreased but only the excitatory and NOT the inhibitory synapse density on dendrites is changed (Fig. 2). This result is consistent with the specificity of RTN4R expression in excitatory neurons (Figure 8 and Extended Data Fig. 10) and indicates that synapses are not simply diluted on dendrites.

2b. It is therefore not surprising that Bai3-dN, which is unable to bind to C1ql, can still rescue the reduction in synapse number observed in Bai3 KOs.

We do agree that in the context of the entirety of our results it is not surprising that the BAI3-ΔN mutant can still rescue the decrease in synapse density since a) this decrease is equally observed in BAI1 and BAI3 KO neurons and b) we show in extensive experiments in this

paper and a structural paper that the BAI1 NTD does not bind to C1qls. Thus, the synapse loss we observe cannot be due to a loss of C1ql binding.

Please note, however, that we do not wish to imply that the many previous studies, including some from our own lab, showing that C1ql-binding to BAI3 is essential for synapse formation in specific circuits is incorrect. Whereas, as discussed above, RTN4Rs are expressed in all excitatory neurons, C1qls exhibit more restricted expression patterns. Their role and requirement are thus much more limited. The reviewer is entirely correct in supposing that our experiments, as described in the present manuscript, did not adequately address this role but addressing this role was not the objective of the current study – instead, we here aimed to analyze the most fundamental functions of BAIs in a reduced system.

2c. As previously stated, I believe that cultured hippocampal neurons may not be an optimal model for assessing the synaptogenic potential of Bai. However, if the synaptogenic potential of Bai overexpression is to be evaluated in hippocampal neurons, the authors should assess the number of synapses as well as spine morphology, rather than synaptic density, by sparse KO + rescue experiments as discussed below.

We agree that hippocampal cultures are limited as a reduced system in analyzing synapse biology, although they also provide advantages. However, the experiment suggested by the reviewer in our opinion is not as easy to interpret as it may seem at first glance. Sparse deletions and sparse overexpression experiments create a competitive environment between cells that renders the results difficult to assess mechanistically. A classical example for this artifact is Liqun Luo's *Science* paper on the sparse deletions of TrkC that caused a dendritic growth phenotype even though TrkC has no function in dendritic growth – it is a simple competition effect (PMID: 25359972), as his lab confirmed with a follow-up paper in *Neuron* revising the conclusions of the *Science* paper (PMID: 33352118). Thus, we are hesitant to employ sparse deletion/rescue experiments because they can be difficult to interpret.

3a. Experimental Methods for KO-Rescue
In a dissociated primary culture, the maturation and number of synapses are significantly influenced by the density of surrounding neurons and glia. For instance, as illustrated in Fig. 5a, the density of neurons appears to be relatively high for the third (Cre+WT) and fifth (Cre +dN) panels, which may influence the synaptic density.

We apologize if the representative images sometimes appear to indicate differences that don't exist and might thus be misleading. In our experiments, all conditions are from the same hippocampal cultures and thus have exactly the same density of neurons. None of the manipulations we apply seem to alter the neuron density. To address this concern, we have updated the representative image for the Cre + dN condition in the new Fig. 5.

3b. Furthermore, the authors' model (Wang et al., Cell 2021) indicates that RTN4Rs expressed on neuronal axons inhibit axon outgrowth by interacting with Bai in glial cells in trans. Consequently, dendrite outgrowth may be influenced in neurons lacking Bai, whereas axon outgrowth should remain unaltered if the surrounding glia is wild-type expressing Bai. To circumvent this issue, it would be better to sparsely express the Cre and rescue constructs and then compare them to neighboring wild-type neurons in the same culture dish. To evaluate the role of glial Bai, the authors should utilize glia-specific promoters to induce Cre expression.

We agree that cell specificity is important but feel that this should be better addressed in an *in vivo* context, as actually also suggested by this reviewer. In our experiments on BAI2 and BAI3, Cre and ΔCre are expressed in both neurons and glia, whereas in our experiments on BAI1, we used a constitutive KO in all cells. Thus, we are studying BAI deletions in both neurons and glia and do not make any claims about the cell-type specificity of BAIs.

As regards the recommendation to perform sparse neuronal manipulations, the reviewer suggested this already above in comment 2c; please see our response there.

3c. A related question is the efficiency of the knockout and rescue processes. Cre was expressed by lentivirus at 4DIV, but it is unclear what percentage of neurons and glia are actually knocked out with Bai2, 3. In Supplement Fig. 4a, it appears that HA-BAI3 rescued by lentivirus is not expressed in all neurons. It is not described how the authors ensured that the neurons being evaluated for electrophysiology and morphology have been successfully knocked out or knocked out and rescued for BAI. For instance, in Supplemental Figure 4a, MAP2 (magenta) is utilized to identify dendrites, and HA (green) is employed for rescue constructs. However, it is also described as magenta was used for Cre.

We agree that the infection efficiency is an important issue and have addressed this issue as described above in our response to comment 4 of reviewer #1. The Cre and ΔCre are fused with EGFP and are localized in the nucleus. To optimize the use of available imaging channels, we labeled MAP2 with Alexa Fluor 488. The nuclear localization of Cre and ΔCre does not interfere with the dendritic MAP2 analysis conducted in this study.

4. The Function of Bai3 Regulated by C1qL
The authors propose that C1qL binding to Bai3 is required for synapse "organization," not synaptogenesis, because Bai3 dN can rescue the reduction in synapse number of Bai3 KOs (Fig. 5b) but it cannot rescue the reduction in mEPSCs (Fig. 6b). However, the specific nature of synapse "organization" remains unclear. If glutamate release from the presynapse (Fig. 6e) is not affected, the authors must examine whether there are abnormalities in the number or arrangement of postsynaptic AMPA receptors.

We agree that we have not investigated how C1ql's organize synapses in the current manuscript. Multiple potential mechanisms could be operating to account for the action of

C1ql's that include changes in the release probability, AMPARs and NMDARs, and many other features. Studying this question unfortunately exceeds the scope of the current study.

5a. Quantitative Methods for Synapses and Synaptic Molecules
The methods for quantifying synaptic morphology and synaptic molecules are not well described. Specifically, authors need to describe how they determined the brightness and size of the puncta of synaptic molecules. It is also unclear how synapses were defined, and the criteria used to select dendrites for measurement of synaptic density, including details such as distance from the cell body and apical/basal dendrites.

We have now added these details to the Methods section of the manuscript to enhance clarity and transparency about our quantification procedures. Thank you for bringing this to our attention. The text now states:

"Quantifications of the synaptic puncta were performed by general analysis in NIS-Elements software (Nikon) as previously described (Trotter et al., 2023). For each batch of samples, the average background intensities for each channel were calculated and subsequently subtracted from the corresponding images. A uniform binary mask was applied consistently across all images within the same batch. Three regions of interest (ROIs) were identified for each neuron along three distinct primary dendrites. The soma was positioned at the center of the image, with each ROI encompassing the entire length of the primary dendrite, extending approximately 100 μm from the edge of the soma to the edge of the image. Synapse density was determined by counting the number of colocalized vGlut1 and Homer1 puncta or vGAT and gephyrin puncta and dividing this by the length of the dendritic segment. The puncta size was calculated by dividing the total binary area of the puncta by the number of objects identified within the ROI. The overall synapse density or puncta size for each neuron was calculated by averaging the densities obtained from the three ROIs. The representative images were processed by Fiji software."

Please note that the approach of measuring synapses as puncta exhibiting co-localized pre- and postsynaptic marker signals is the most rigorous method currently available. Please also note that in measurements of synapse density using immunocytochemistry, the numbers obtained are never absolute since small synapses may be overlooked and two adjacent synapses may merge into one detectable punctum. Thus it is important to use highly standardized measurements that employ the same parameters for all conditions for reliable results, and only relative results are comparable.

5b. In Fig. 7, the synaptic localization of Bai3 is examined, yet the method utilized for quantification is not well described. For instance, in Fig. 7a, the puncta exhibit a range of sizes, and it is not evident which ones represent pre- and postsynaptic or glial cells. Additionally, in the merged image in Fig. 7, it is not possible to distinguish which molecules are co-localized by color. For example, it is not possible to distinguish between the following combinations because the merged images will become white in all cases:

- (1) Cyan (HA) + Yellow (Homer): merge [Homer having BAI]
- (2) Cyan (HA) + Red (vGluT1): merge [vGluT1 having BAI]
- (3) Cyan (HA) + Yellow (Homer) + Red (vGluT1): merge [Synaptic BAI]
In addition, Homer alone (yellow) is indistinguishable from synapses (yellow (Homer) + red (vGluT1)).
Upon closer examination of the image, the majority of HA-BAI3 signals do not appear to overlap the "synapse." This does not align with the assertion (page 9, line 280) that "the majority of transfected BAI3 is in the synapse." Additionally, the signals that overlap VGluT1 and Homer are relatively small and do not align with the quantitative results shown in Fig. 7f, g. It is recommended that the pseudocolor of the image be altered to demonstrate the degree of overlap between the colors, and that the quantification method be described or reexamined. The authors also need to show enlarged views of synapses.

We appreciate the concerns of the reviewer indicating that the few sample images are insufficient to fully illustrate the conclusions. We completely agree. Unfortunately, however, space constraints do not allow us to include a large number of different sample images as this would require a lot of journal space. To address this conundrum, we are making all of our raw data, including the complete set of images, available in a freely accessible database. This will allow anyone interested to explore our results, view all images, and critically assess our conclusions.

Moreover, we truly value the reviewer's input on the figures and have made several updates: we have changed the color combinations, provided enlarged views, adjusted the terminology, and made revisions in the new Fig. 7. However, it is important to note that our STED imaging captures only a single layer snapshot without a "z" dimension. As a result, the relative positioning of BAI3 with vGlut1 and Homer1—defining what constitutes a "synapse"—can vary based on the imaging angle. In this approach, it is impossible to determine that a signal is not in a synapse – for this, confocal imaging or 3D-STED is required. Additionally, our quantification of “fraction” focused on the number of puncta rather than their area. The only purpose of the STED imaging was to ascertain that at least a fraction of BAI3 molecules is synaptic. They do not exclude the possibility that all are synaptic.

6a. Binding of Bai2 to C1qls
The authors assert that C1qls bind “weakly” to Bai2 (lines 129-130; 312-313) based on the size-exclusion chromatography, showing that the N-terminal domain (N-TSR4) of Bai2 and the globular domain of C1ql3 co-migrate slightly. However, the term "weakly" should not be used without assessing the Kd value. It is also not a fair comparison to use the full length of the extracellular domain for Bai1 and Bai3, but only a part of it for Bai2.

We agree that Kd measurements would have been better but feel that the data justify the conclusion that C1qls bind only weakly to BAI2. The term ‘weak’ does not imply any specific affinities, which were not measured. As described in our response to point 1 of reviewer #1, we have now addressed this important point by systematically analyzing the interactions of

all C1ql isoforms with all BAIs. In addition, we obtained a cryo-EM structure of the NTD of BAI3 in a complex with C1ql3 C1q-like domains, which is consistent with the present conclusions (Y. Miao et al., Comm. Biol. 2025).

6b. Given that Bai2 lacks a CUB domain, which is required for Bai1 or Bai3 to bind to C1qls, the authors must carefully examine the interaction between full-length C1qls (not only the globular domain) and Bai2.

Please note that BAI2 actually does have the same 'variant' CUB domain as Bai1 and Bai3. The reviewer may be misinformed by older reviews in concluding that BAI2 lacks a 'CUB' domain, but our structural data clearly show that all BAIs contain a very similar atypical CUB domain (Y. Miao et al., Comm. Biol. 2025). This domain includes additional loops that are absent from classical CUB domains. The BAI3 NTD residues that bind C1qls are lacking in BAI1, explaining why it doesn't bind C1qls, and are mostly present in BAI2, explaining why it weakly binds C1qls.

6c. In addition, the term "C1qls" is not accurate since only C1ql3 is utilized as a ligand, yet no other C1ql is subjected to testing.

Agreed. As described in our response to point 1 of reviewer #1, we have now addressed this point by systematically analyzing the interactions of all C1ql isoforms with all BAIs. The new data are shown in the new Extended Data Fig. 2.

6d. Furthermore, despite the authors' assertion that "It is unclear whether C1qls bind to other BAIs besides Bai3" (line 120), C1ql1 was previously demonstrated to lack binding affinity for Bai1 or Bai2 in cell-based binding assays (Matsuda et al, Neuron, 2016).

Again agreed. We have now changed the wording of this sentence, although the cell-based binding assays (that we replicate here) would miss the weak binding of BAI2 to C1ql3 that we demonstrate.

Minor points:
1. In Figures 3 and 6, the results of cumulative frequency plots of mEPSC and mIPSC demonstrate notable differences between WT and KO. However, these findings are not explicitly addressed in the text, which may lead to confusion among readers. For instance, Fig. 3 illustrates a significant difference in mEPSC amplitude between WT and Bai1 KO. Additionally, there is a significant difference in mIPSC frequency between WT and Bai1, Bai2 KO.

We have revised and expanded the text to avoid any confusion that might be caused by our electrophysiological recordings. Scientists not familiar with synaptic physiology may not always realize that the differences observed in cumulative plots, although always statistically significant, are of limited biological significance for assessing the absolute frequency and amplitude of synaptic events. The value of the cumulative plots is to reveal the possible

emergence of subsets of synapses, which is why they are being presented. In electrophysiological experiments, only the bar graphs are meaningful for measuring absolute amplitudes and frequencies (and should not be normalized, of course). These bar graphs unequivocally demonstrate in our manuscript, we believe, that there are major phenotypes in the mEPSC frequency induced by the BAI1 and BAI3 KOs but no other changes.

2. In Fig. 6, the size of evoked EPSCs reaches 4 nA, which is considerably larger than what would be expected in hippocampal dissociated culture. Please verify that there are no mistakes.

Evoked EPSC amplitudes of this size are commonly observed in our and other labs. Please see a few examples:

Broker-Lai J, Kollewe A, Schindeldecker B, Pohle J, Nguyen Chi V, Mathar I, Guzman R, Schwarz Y, Lai A, Weissgerber P et al (2017) Heteromeric channels formed by TRPC1, TRPC4 and TRPC5 define hippocampal synaptic transmission and working memory. EMBO J 36: 2770-2789

McCabe MP, Cullen ER, Barrows CM, Shore AN, Tooke KI, Laprade KA, Stafford JM, Weston MC (2020) Genetic inactivation of mTORC1 or mTORC2 in neurons reveals distinct functions in glutamatergic synaptic transmission. Elife 9

Sando, R., and Südhof, T.C. (2021) Latrophilin GPCR Signaling Mediates Synapse Formation. E-Life 10, e65717.

Dai, J., Patzke, C., Liakath-Ali, K., Seigneur, E., and Südhof, T.C. (2021) GluD1, A signal transduction machine disguised as an ionotropic receptor. Nature 595, 261-265.

3. Membrane capacitance is a sensitive measure of surface area, which is directly related to the elongation of dendrites and axons. However, the text states that "there is no difference in membrane capacitance or input resistance in BAI-deficient cells" (lines 189-192). Please verify the data.

The reviewer is correct that the capacitance measures the neuronal surface area but, unfortunately, voltage clamp methods cannot effectively clamp axons and dendrites. Voltage clamping only extends to the soma and possibly proximal dendrites. Thus, no change in capacitance is expected with changes in dendrite and axon lengths. The measurements are included as evidence that the soma size has not changed consistent with the morphological results and the passive membrane properties are not dramatically altered.

Reviewer #3

In this manuscript, Wang et al. utilize mouse hippocampal cultures to investigate the roles of BAI adhesion-GPCRs and their known ligand interactions in hippocampal neuronal development. In agreement with previous studies, the authors confirm that BAI1 and BAI3 restrict axonal and dendritic growth and promote excitatory synapse formation, whereas the authors do not detect a role for BAI2 in these processes. The authors also examine the ligand-binding properties of BAIs, verifying that secreted C1ql proteins interact with both BAI3 and BAI2 (but not BAI1), in contrast to GPI-linked RTN4Rs, which the group previously reported bind to BAI1 and BAI3 (but not BAI2). Using rescue experiments, the authors further show that RTN4R-binding to BAI3 is essential for both limiting axonal/dendritic arborization and enhancing synapse formation, while C1ql-binding to BAI3 is only required for regulating excitatory synapse organization/ function. Based on these findings, the authors conclude that a dual ligand-binding mechanisms controls BAI3's neurodevelopmental functions, with BAI3-RTN4R binding required for axonal/dendritic growth regulation and both RTN4R- and C1ql-binding essential for BAI3-mediated synapse assembly.

Overall, this is an interesting study that extends our understanding of the mechanisms by which BAI adhesion-GPCRs regulate neurodevelopment. The manuscript is well-written, and the data presented are clear, thorough, and generally convincing. While some of the results are confirming previously reported functions for BAIs and their ligands, this manuscript comprehensively assesses all three BAI family members in hippocampal neurons and compares the specific roles of BAI3's two known ligands. To further strengthen this manuscript, however, the following issues should be addressed.

We thank the reviewer for her/his comprehensive and constructive comments.

Major concerns:

*1. * In Figure S1, the authors compare the ability of BAI1, BAI2 and BAI3 to interact with C1ql3. Why was a different extracellular fragment of BAI2 used instead of the directly analogous fragment used for BAI1/3? The authors could also provide additional information in the figure legend, clarifying what is shown in the size-exclusion chromatography and Coomassie-stained SDS-PAGE gel in terms of BAI binding to C1ql3 (note shifts showing positive interactions).*

Agreed. We have now improved the figure legend. Moreover, we agree that it would have been better to use the same fragments in ED Figure 1. However, we were unfortunately unable to express and purify full-length BAI2 extracellular protein fragments or full-length C1qls in large quantities, forcing us to use the proteins that we could obtain in sufficient amounts. As described in our response to point 1 of reviewer #1, we have now validated the results using a uniform approach that analyzes all combinations of binding partners (new

Extended Data Fig. 2). In addition, in a separate paper published by our group (Miao et al., 2025), we reported the structure of the C1ql3-BAI3 complex. Sequence alignment of C1qls and BAIs indicate that the contact residues of the BAI3-C1ql3 complex are conserved in all C1ql homologs but are retained only in BAI2 and BAI3, whereas BAI1 lacks these residues.

*2. * Figure 3 is a bit confusing. While the summary bar graphs agree with the text, the cumulative fraction plots of the inter-event intervals and peak amplitudes report statistical differences that appear to contradict the text and the bar graphs. These are not mentioned in the text. Can this result be further explained? Is a distinction being drawn between mEPSC amplitude and peak amplitude, for example? What is being added by these histograms and why aren't they discussed in the text? What comparisons are being denoted in these graphs? This same issue applies to Figure 6.*

We agree. We have now discussed the cumulative plots in detail. In interpreting mEPSC measurements, we and others use the averages ± SEMs of the mEPSC frequency and amplitude as rigorous readouts of spontaneous synaptic activity that report primarily on the number and release probability of synapses (frequency) and on the presynaptic vesicular glutamate content and the postsynaptic AMPA-receptor content (amplitude). We use the cumulative plots, in turn, to assess possible heterogeneity between synapses and to detect potential subsets of synapses that would not be detectable in the assessment of the averages. Because the cumulative plots use individual events as statistical 'n's', they produce almost always statistically significant differences even if these differences are small and biologically meaningless. These statistical differences are therefore generally considered biologically not significant if the averages show no differences because the use of individual events as statistical n's artificially exaggerates the biological differences. The approach we are using is standard in electrophysiology, although some papers use the cumulative distributions to make conclusions that may not always be justified.

** In Figures 4/S4, the authors show that ligand-binding mutations in BAI3 reduce (but do not eliminate) BAI3's synaptic localization. However, based on the staining method used, it remains unclear whether the BAI3 mutations affect cell surface localization. An addition live-staining experiment with intact plasma membrane could clarify this issue.*

Agreed. This is an important point that is similar to point #3 raised by reviewer #1. We have now addressed this point as described in response to the comment of reviewer #1.

** The authors note that their results regarding the C1ql-BAI3 interaction are distinct from those previously reported by themselves and others (only required for synaptic function, not formation). Moreover, the authors do not identify a role for BAI2 in either axonal/dendritic growth or synapse development. However, the authors only performed their experiments in hippocampal mouse cultures. It is important to stress in the discussion*

that the findings in this paper may be specific for hippocampal neurons and that neurons in different brain regions may be differentially regulated by the BAI family members.

We completely agree and have emphasized the important point made by the reviewer even more in the revised discussion.

** While the authors acknowledge in the discussion that they did not distinguish between glial and neuronal BAIs, they previously argued that glial BAI1/3 restrict axonal/dendritic growth while neuronal BAI1/3 regulate excitatory synaptogenesis (and they allude to this in their model figure (Figure 8)). Though they show in Figure S4 that they can express BAI3 constructs in both neurons and astrocytes, it would be informative to provide some quantification of the extent of BAI1/2/3 loss and BAI3 rescue construct expression in astrocytes vs neurons in their hippocampal cultures. This issue could also be addressed by independently manipulating BAI levels in neurons vs astrocytes.*

Again, we agree that this is an important point. In the new Extended Data Fig. 4, we have quantified the transduction efficiency of Cre and ΔCre in both neurons and glia. Our data show that nearly all neurons express ΔCre and Cre, and a significant percentage of glial cells also express these constructs. The BAI3 rescue constructs were driven by the same synapsin promoter used for Cre and ΔCre.

As a minor note, please consider that our previous results showed that glial BAI's only regulate axon growth but not dendrite growth.

** On lines 198 to 202, the authors argue that "the total number of synapses in the BAI1- and BAI3-deficient neurons is likely not changed given that these neurons exhibit an increase in dendritic and axonal growth but a decrease in synapse density per dendritic length. In view of this synapse number constancy, the decrease in synaptic mEPSCs is thus remarkable, suggesting that the BAI1 and BAI3 deletions also impair synaptic strength and not only synapse density". If this is the case, why is mEPSP amplitude not affected? To show that synapse constancy is indeed achieved, the authors need to analyze different domains of the neurons (primary, secondary, tertiary dendrites). If this was done, the methods should make this clear.*

We have rephrased the sentences queried by the reviewer as suggested. As a side note, please recall that the mEPSC amplitude does NOT depend on synapse numbers and thus would not be expected to be changed.

** The authors infected hippocampal cultures from BAI3 cKO mice with lentiviruses expressing Cre on DIV4, infected rescue lentiviruses at DIV6, and sparsely transfected neurons with tdTomato at DIV9. Did the authors confirm that the neurons they analyzed were affected by all 3 manipulations? Please clarify?*

We agree that these three sequential manipulations could potentially induce artifacts by themselves. To control for this possibility, we have always compared results among conditions in which the cultures had always been subjected to all three sequential manipulations. It is possible that these manipulations might cause a partial stress response or some other cellular reaction that could conceivably influence neuronal properties but since we subjected the neurons to always the same manipulations, we are fully controlling for this possibility.

** Different KO models were used for BAI1, BAI2, and BAI3. The deletion of BAI1 is constitutive while the deletion of BAI2 and BAI3 are more acute. Could this contribute to the different results seen in punctal size analysis between BAI1 and BAI3 shown in Figure 2 (i.e. compensation in the BAI1 model)?*

We agree that this is a possibility and have now discussed this in the paper.

Minor concerns:

** Fig. 5 (page 8). If the decrease in excitatory synapses in the ΔN BAI3 mutant is "slight" and "statistically insignificant", then perhaps it is more accurate to say that there is no* decrease in excitatory synapses in this condition.

We have reworded the noted sentence.

REVIEWERS' COMMENTS

Reviewer #1 (Remarks to the Author):

The authors have addressed most of my concerns and the data presented are really interesting.
There is only one minor concern left regarding the potential presence of a truncated protein in the KO condition.
I agree with the authors that given the design of the BAI1 KO, one can be confident that no protein that could have a functional impact is produced. In the case of the BAI3 KO condition, it is less clear. The authors refer to the Kakegawa et al. (2015) paper. In that article, the authors document the KO via immunolabeling of tissue sections with a homemade anti-Bai3 antibody. I couldn't find any information on the characterization. of this antibody and immunolabeling does not test whether a truncated protein is produced. If a good antibody against BAI3 for immunoblotting is unavailable to the authors, then at least a qRT-PCR using a probe outside of the deleted region might be helpful to measure how much truncated mRNA is produced (the mRNA from the mutated allele might be subject to nonsense-mediated decay which would be good).
We thank the reviewer for this question. In Extended Data Fig. 3j, we further validated BAI3 deletion using qRT-PCR targeting exons outside the loxP-flanked regions in hippocampal and cortical cultures from BAI3 cKO mice.

Reviewer #3 (Remarks to the Author):

I am satisfied that the authors successfully addressed all my concerns. I believe it is a strong, interesting manuscript worthy of publication in Nature Communications.

We thank the reviewer for your positive comments.

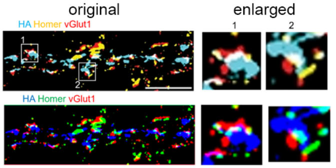
original
enlarged
HA Homer vGlut1
1
2
HA Homer vGlut1
1
2
